# Membrane-Active Cyclic Amphiphilic Peptides: Broad-Spectrum Antibacterial Activity Alone and in Combination with Antibiotics

**DOI:** 10.1021/acs.jmedchem.2c01469

**Published:** 2022-11-28

**Authors:** Eman H. M. Mohammed, Sandeep Lohan, Tarra Ghaffari, Shilpi Gupta, Rakesh K. Tiwari, Keykavous Parang

**Affiliations:** † Center for Targeted Drug Delivery, Department of Biomedical and Pharmaceutical Sciences, Chapman University School of Pharmacy, Harry and Diane Rinker Health Science Campus, Irvine, California92618, United States; ‡ Department of Chemistry, Faculty of Science, Menoufia University, Shebin El-Koam51132, Egypt; § AJK Biopharmaceutical, Irvine, California92617, United States

## Abstract

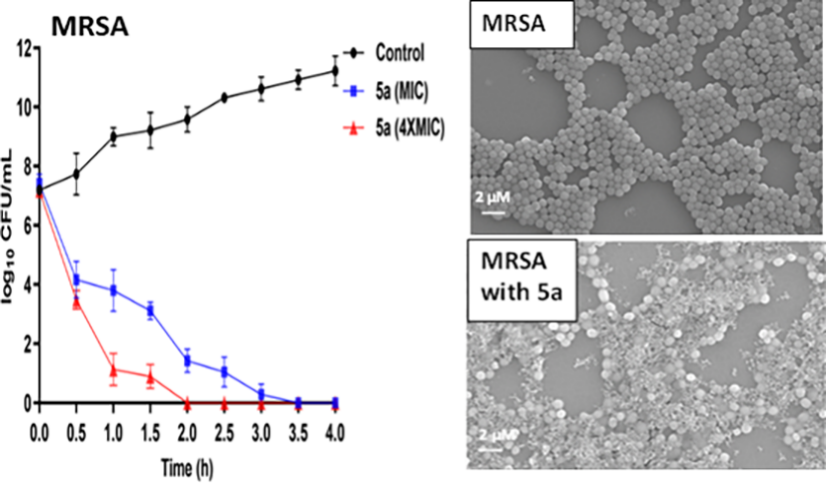

We designed a library of 24 cyclic peptides containing arginine (R) and tryptophan (W) residues in a sequential manner [R_*n*_W_*n*_] (*n* = 2–7) to study the impact of the hydrophilic/hydrophobic ratio, charge, and ring size on the antibacterial activity against Gram-positive and Gram-negative strains. Among peptides, **5a** and **6a** demonstrated the highest antimicrobial activity. In combination with 11 commercially available antibiotics, **5a** and **6a** showed remarkable synergism against a large panel of resistant pathogens. Hemolysis (HC_50_ = 340 μg/mL) and cell viability against mammalian cells demonstrated the selective lethal action of **5a** against bacteria over mammalian cells. Calcein dye leakage and scanning electron microscopy studies revealed the membranolytic effect of **5a**. Moreover, the stability in human plasma (*t*_1/2_ = 3 h) and the negligible ability of pathogens to develop resistance further reflect the potential of **5a** for further development as a peptide-based antibiotic.

## 1 Introduction

Antibiotics are valuable tools for fighting against bacterial infections. However, antibiotic resistance^1^ is a naturally occurring process. According to the CDC 2019 Antibiotic Resistance (AR) Threats Report, infections caused by antibiotic-resistant germs surpassed 2.8 million, with a mortality rate exceeding 35,000 in the U.S. each year.^1^ Many of these infections have no treatment options. Bacterial infections spread around us in the communities, food supplies, water, and soil. However, serious infections, especially with multidrug-resistant pathogens, are widely dispersed in healthcare facilities like hospitals, clinics, or nursing homes, called healthcare-associated infections (HAIs).^1,2^ Unfortunately, antibiotics innovation and development entered a dark age after the 1960s because of the rapid emergence of resistance.^3^ Consequently, the generation of novel medications to control and treat infections caused by multidrug-resistant pathogens has become a pressing priority for the scientific community.

In recent years, antimicrobial peptides (AMPs) have been considered the best alternative to overcome multidrug-resistant pathogen infections.^4−6^ The discovery of AMPs dates back to 1939, with gramicidin as the first recognized AMP.^7^ AMPs are identified from the innate immune system for multicellular organisms. They are produced as the first line of defense against invading microbial infections.^8,9^ As part of the innate immunity, AMPs directly kill bacteria by acting at the membrane^10^ or via inhibiting macromolecular functions, leading to bacterial cell death.^11^ Furthermore, AMPs can kill pathogens by directing cytokines to the infection area, causing increased immunological responses.^12^ AMPs provide several advantages over traditional antibiotics, such as poor induction of resistance; broad-spectrum activity against a wide variety of bacteria, fungi, protozoans, viruses, and surprisingly even cancerous cells; lower toxicity to the host cells; being less harmful to both the environment and consumers; synergistic effects on the antimicrobial activity of antibiotics; and rapid killing or inhibiting of the pathogens.^13−20^

Most of the AMPs are cationic and have net positive charges ranging from +2 to +9 with an amphipathic arrangement that involves separate hydrophobic and hydrophilic domains.^21,22^ The hydrophilic face consisting of polar cationic residues, such as arginine (R) and lysine (K), provides the source of electrostatic interactions between the peptide and the lipopolysaccharide (LPS) or lipoteichoic acid (LTA) that are the negatively charged elements of the microbial cell membrane.^23,24^ On the other hand, the hydrophobic face is composed of nonpolar residues, such as tryptophan (W), alanine (A), glycine (G), and leucine (L), providing lipophilicity that ultimately disturbs the membrane and creates pores across the membrane leading to cell death.^23,25,26^ Some AMPs cross the membrane without destroying it^27,28^ and act on intracellular targets, for instance, binding to RNA, DNA, or histones,^29−31^ stopping DNA-dependent enzymes,^32^ preventing the synthesis of essential outer membrane proteins,^33^ and binding to the ribosome and lipid II.^34,35^

Peptide-based antibiotics have been approved by the U.S. Food and Drug Administration (U.S. FDA) and are already on the market, and some others are in the clinical trial stage. For instance, vancomycin is one of the most important frontline antibiotics approved by the U.S. FDA in 1958 to treat infections caused by Gram-positive bacteria, especially methicillin-resistant *Staphylococcus aureus* (MRSA). It was isolated by a fungus named *Streptomyces orientalis* and contained impurities.^36^ Vancomycin has been used intravenously. Daptomycin was approved in 2003 to treat infections caused by Gram-positive bacteria.^37^ In addition to gramicidin, polymyxin, and bacitracin, three more peptide-based antibiotics, telavancin (2009),^38^ dalbavancin (2014),^39^ and oritavancin (2014),^39^ have been approved by the U.S. FDA for the treatment of *S. aureus* infections.

Meanwhile, pharmaceutical companies are expanding their efforts to use AMPs as commercially available medications.^40^ For example, NP339 (Novamycin) is being studied by Novabiotics Company in the preclinical stage.^41^ Another example is LTX-109 (Lytixar), developed for topical treatments by Lytix Biopharma Company, which is in the phase I/II trial for the treatment of *S. aureus* infection, including MRSA.^42^

Despite the many advantages of AMPs, they also suffer from some shortfalls, such as hemolytic activity toward human red blood cells, protease instability, salt and serum sensitivity, and high production cost.^9,43^ Therefore, developing more effective AMPs to overcome these limitations is necessary to enhance their therapeutic applications. For maximum efficacy *in vivo* and *in vitro*, many strategies have been employed to improve the overall properties of AMPs, such as optimization of sequences, truncation, modification of hybrid analogs, and redesign of parent peptide sequences via amino acid substitution.^44−47^ These simple strategies assist in overcoming barriers and speed up the development of the clinical application of AMPs.^27,48,49^

Many AMPs contain W and Residues. These residues are known to mediate membrane disruption and/or cell entry.^56,57^ R and W residues possess some important chemical features that make them suitable components of antimicrobial peptides. R side chains are always predominantly charged even when buried in a hydrophobic microenvironment that is because of the high equilibrium acid dissociation constant (p*K*_a_ value) of the arginine guanidinium group of 13.8 ± 0.1.^58^ The unusual ability of the R side chain to remain ionized, unlike the other ionizable amino acids such as lysine and even in microenvironments that are typically incompatible with charges, is attributed to three main reasons: (I) the positive charge delocalization of the guanidinium moiety over many atoms involved in a conjugated Y−π system,^59^ (II) the conformational flexibility of the R long side chain,^60^ and (III) its high intrinsic p*K*_a_ value.^58^

Tryptophan is hydrophobic due to its aromatic uncharged indole side chain, which possesses an extensive π–electron that gives rise to a significant quadrupole moment, resulting in negatively charged clouds enabling tryptophan to participate in cation−π interactions.^61^ The cationic guanidinium group from the R side chain can be bonded to the aromatic pi face of W through cation−π noncovalent forces to complement each other well for antimicrobial peptides.^55^

Several quantitative structure–activity relationships (QSAR) studies demonstrated the importance of amino acid composition to antimicrobial activity, especially the model peptides with R and W as repeating pharmacophore units.^62,63^ Moreover, QSAR studies indicated that RW motifs could retain activity despite a lack of defined secondary structure.^64,65^

**Figure 1:**
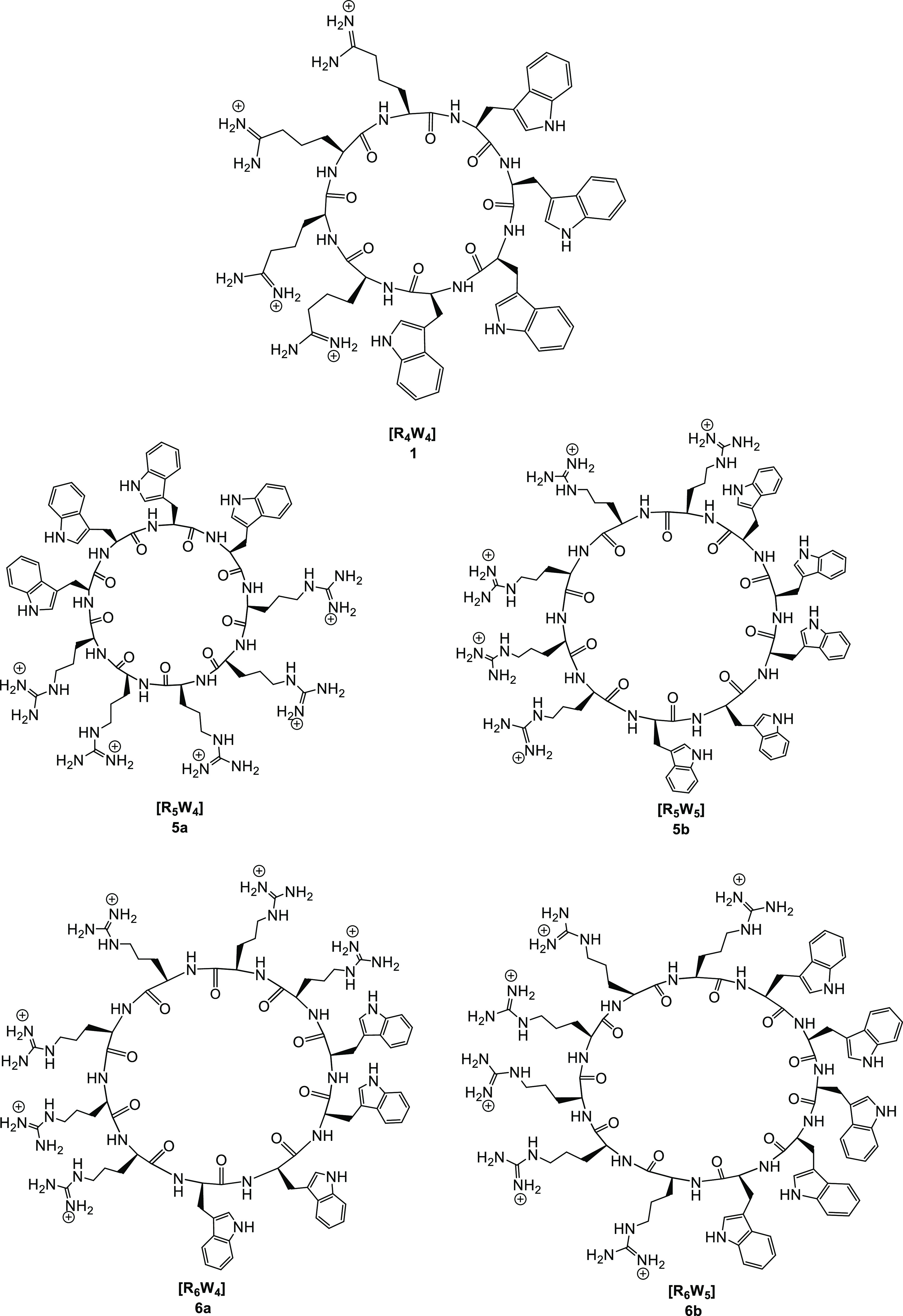
Representative chemical structures of cyclic peptides [R_4_W_4_] (**1**), [R_5_W_4_] (**5a**), [R_5_W_5_] (**5b**), [R_6_W_4_] (**6a**), and [R_6_W_5_] (**6b**).

Our group has previously discovered and reported that cyclic peptides [R_4_W_4_] (Figure 1) and [W_4_KR_5_] containing W and R residues assembled in an ordered manner to have antibacterial activity against multidrug-resistant pathogens, especially, against Gram-positive bacteria.^50,51^ Moreover, [R_4_W_4_] showed cell-penetrating properties in eukaryotic cells and bactericidal effects in the [R_4_W_4_]/tetracycline combination against MRSA and *E. coli*.^50^ Cyclic peptides showed higher antibacterial activities compared to the corresponding linear counterparts. Arginine and tryptophan with an l-configuration were required to generate optimal activity.^52^ Moreover, [R_4_W_4_] was used to develop covalent conjugates with antibiotics. However, the conjugation diminished the activity.^53^ The use of [R_4_W_4_], along with first-line antituberculosis medications, reduced the side effects of antibiotics and improved the immune responses to get rid of the active *Mycobacterium tuberculosis* (Edman strain) infection. The activity was determined against infected peripheral blood mononuclear cells (PBMCs) by quantification of the intracellular survival of the bacteria.^54^

In this study, a library of 24 cyclic peptides was rationally designed based on the [R_*n*_W_*n*_] sequence as the model scaffold to study the impact of the hydrophilic/hydrophobic ratio, charge, and ring size on antibacterial activity. This study assisted in establishing a structure–activity relationship (SAR) to understand further the role of R and W residues and the cationic/hydrophobic ratio on antibacterial activity and other essential properties. Designed peptides were successfully synthesized using Fmoc/tBu solid-phase peptide synthesis. All synthesized peptides were evaluated for their antibacterial efficacy against Gram-positive and Gram-negative bacteria, including multidrug-resistant strains. The salt effect and synergistic activity were also evaluated for a better understanding of the role of the cyclic peptide as a promising alternative antibacterial agent and the revival of traditional antibiotics. AMPs have been known to achieve their antibacterial effect in a number of different modes; however, an initial interaction with the bacterial membrane is necessary.^10,55^ The interactions with bacterial membranes were also evaluated using calcein dye leakage and scanning electron microscopy (SEM).

## 2 Results and Discussion

### 2.1 Chemistry

We designed a library of cyclic amphiphilic peptides using R and W residues in an ordered sequential manner [R_*n*_W_*n*_] (*n* = 2–7) by systematically changing the number of R and W residues to study the impact of the hydrophilic/hydrophobic ratio, charge, and ring size on antibacterial activity. Moreover, this study aimed to determine the optimal number of cationic and hydrophobic residues required to target bacteria effectively and to obtain a promising antibacterial agent. Figure 1 shows representative cyclic peptides in this class.

**Scheme 1:**
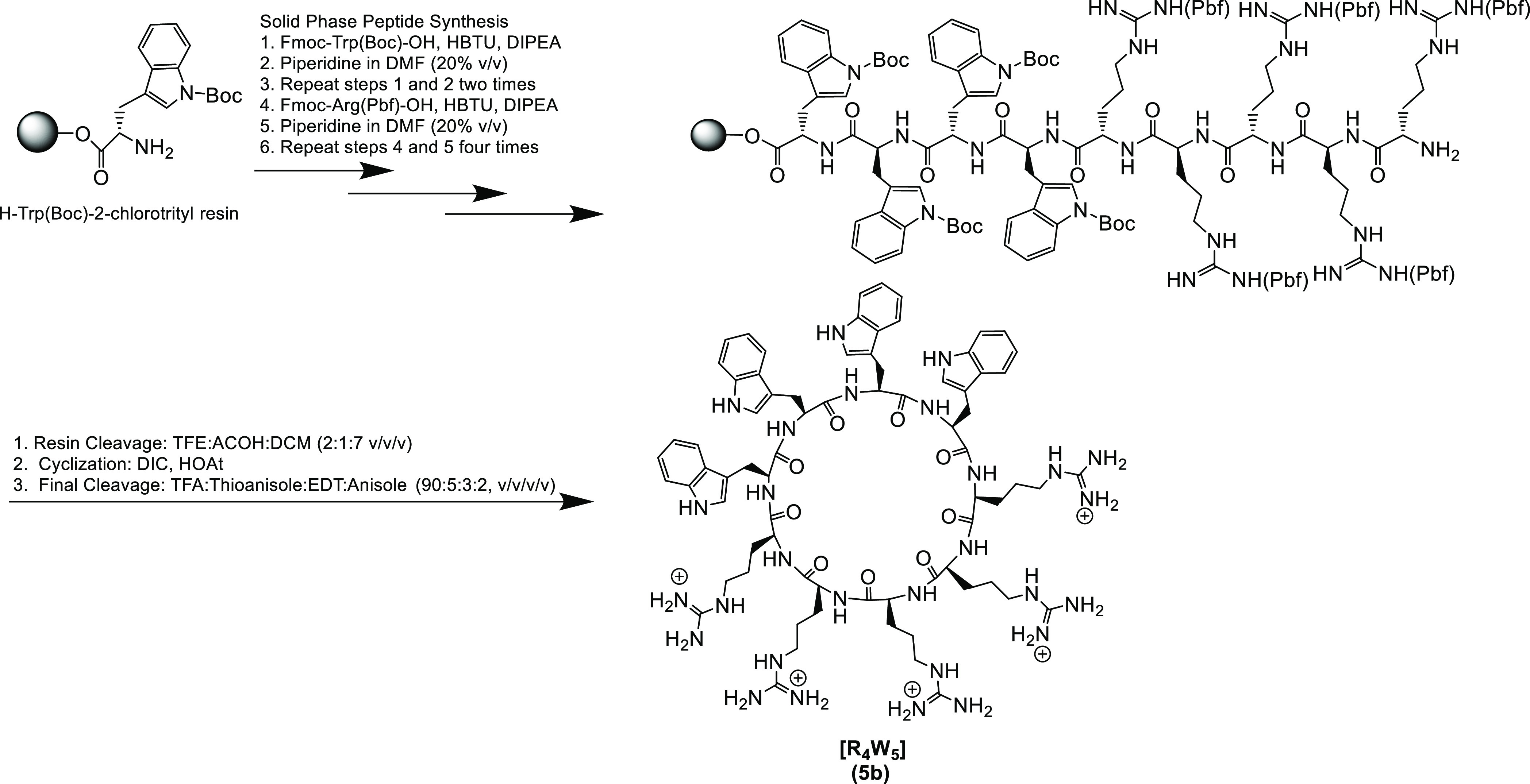
Solid-Phase Synthesis of [R_5_W_4_] (**5b**) as a Representative Example

A representative example of the synthetic process for the synthesis of compound **5b** is depicted in Scheme 1. The peptides were synthesized using Fmoc/tBu solid-phase peptide synthesis. The first step in the synthesis of peptides was assembling W and R on the tryptophan-preloaded H-Trp(Boc)-2-chlorotrityl resin as a solid support. Assembly was started with W residues followed by R residues using coupling and deprotecting reagents, as described in the Experimental Section.

Cyclic peptides were obtained after the cleavage of the assembled side chain-protected linear peptides from the resin followed by *N*- to *C*-cyclization in the solution phase. The assembled side chain-protected peptide was detached from the resin in the presence of trifluoroethanol (TFE)/acetic acid/dichloromethane (DCM) [2:1:7 (v/v/v)]. After evaporation of the solution, the resultant solid was dissolved in an anhydrous dimethylformamide (DMF)/DCM mixture and stirred with 1-hydroxy-7-azabenzotriazole (HOAt) and *N*,*N*-diisopropylcarbodiimide (DIC) under nitrogen overnight at room temperature to accomplish the cyclization. The final cleavage for the cyclic peptides was achieved in the presence of a freshly prepared cleavage cocktail R containing trifluoroacetic acid (TFA)/thioanisole/1,2-ethanedithiol (EDT)/anisole (90:5:3:2, v/v/v/v).

The purification process for synthesized cyclic peptides proceeded via reversed-phase high-performance liquid chromatography (RP-HPLC). The purity of compounds was found to be ≥95%. All synthesized peptides were characterized using high-resolution matrix-assisted laser desorption/ionization (MALDI) mass spectroscopy.

### 2.2 Antibacterial Activity

#### 2.2.1 MIC Determination

AMPs can play multiple roles in treating chronic infections and work as antimicrobial, antiattachment, and antibiofilm agents.^66^ Structural properties of AMPs, such as the size, sequence, cationic nature, and amphipathicity, help to possess many modes of action. In general, AMPs affect the transmembrane potential via increasing membrane permeation and causing cell lysis leading to cell death. Moreover, they can neutralize or disaggregate the lipopolysaccharide, the main endotoxin responsible for Gram-negative infections.^66,67^ The amphipathic structure is crucial for the bactericidal activity of AMPs.^68^

Herein, we developed a library of 24 cyclic peptides rich in R and W residues and evaluated them as AMPs based on the following rationale. The positively charged guanidine unit of R facilitates the initial binding of peptides to the membrane surfaces via electrostatic interactions. Bacterial membranes consist of negatively charged phospholipid head groups like phosphatidylglycerol, cardiolipin, or phosphatidylserine and display high affinity for cationic positively charged R residues.^69^ Additionally, R provides the hydrogen bonding geometry necessary for membrane translocation.^56^ Moreover, W demonstrates a crucial function in membrane association due to its substantial preference for the interfacial regions of lipid bilayers, leading to the enhancement of the antimicrobial properties of the peptides.^51,52,70^

The minimum inhibitory concentration (MIC) values for synthesized cyclic peptides were measured using a microbroth dilution assay to determine the antibacterial activity against Gram-positive and Gram-negative strains. Four bacterial strains, including two multidrug-resistant strains (Gram-positive methicillin-resistant *Staphylococcus aureus* (*S. aureus* BAA-1556, MRSA) and Gram-negative *Klebsiella pneumoniae* (*K. pneumoniae* BAA-1705)) and two nonresistant strains (Gram-negative *Pseudomonas aeruginosa* (*P. aeruginosa* 27883) and *Escherichia coli* (*E. coli* 25922)) were used for antibacterial screening (Table 1). Meropenem, vancomycin, and polymyxin B were used as antibiotic controls.

**Table 1:** Antibacterial Activity of Designed Cyclic Peptides

		MIC (μg/mL)^a^					
peptide code	peptide sequence	MRSA^b^ (ATCC BAA-1556)	*K*. *pneumoniae*^c^ (ATCC BAA-1705)	*P*. *aeruginosa*^d^ (ATCC 27883)	*E*. *coli*^d^ (ATCC 25922)	HC_50_^f^(μg/mL)	therapeutic index^i^
**1**	[R_4_W_4_]	4	32	64	16	175	43.75
**2a**	[R_2_W_3_]	32	256	256	256	120	3.75
**2b**	[R_2_W_4_]	64	256	256	256	85	1.33
**3a**	[R_3_W_3_]	16	64	128	32	155	9.69
**3b**	[R_3_W_4_]	8	64	256	32	105	13.13
**3c**	[R_3_W_5_]	128	128	64	128	80	0.63
**3d**	[R_3_W_6_]	64	128	256	64	65	1.02
**3e**	[R_3_W_7_]	256	64	256	256	60	0.23
**3f**	[dR_3_W_7_]	64	256	256	256	ND^e^	ND^e^
**4a**	[dR_4_W_4_]	8	64	64	16	110	13.75
**4b**	[R_4_W_5_]	8	128	128	64	100	12.50
**4c**	[R_4_W_6_]	128	256	128	128	75	0.59
**4d**	[R_4_W_7_]	256	256	>256	128	60	0.23
**5a**	[R_5_W_4_]	4	32	32	16	340	85.00
**5b**	[R_5_W_5_]	4	32	32	16	230	57.50
**5c**	[R_5_W_6_]	>256	>256	>256	>256	140	>0.54
**5d**	[R_5_W_7_]	>256	>256	>256	>256	115	>0.45
**6a**	[R_6_W_4_]	8	64	16	32	265	33.12
**6b**	[R_6_W_5_]	8	64	16	32	255	31.88
**6c**	[R_6_W_6_]	16	64	128	64	170	10.63
**6d**	[R_6_W_7_]	64	256	>256	128	85	1.33
**7a**	[R_7_W_4_]	16	64	32	32	190	11.88
**7b**	[R_7_W_5_]	8	ND^e^	ND^e^	64	175	21.88
**7c**	[R_7_W_6_]	32	ND^e^	ND^e^	64	170	5.31
**7d**	[R_7_W_7_]	16	ND^e^	ND^e^	32	140	8.75
**Mero**	meropenem	2	16	1	1	ND^e^	
**Vanco**	vancomycin	1	512	256	256	ND^e^	
**Poly B**	polymyxin B	64	1	1	2	ND^e^	

a The minimum inhibitory concentration (MIC) is the lowest concentration of the peptides that inhibited bacterial growth.

b Methicillin-resistant bacterial strain.

c Imipenem-resistant bacterial strain.

d Nonresistant strain.

e ND, not determined.

f HC_50_ is the concentration of a peptide in μg/mL at which 50% hemolysis was observed.

i The therapeutic index is calculated based on HC_50_/MIC against MRSA. The table presents the data of three independent experiments performed in triplicate.

Peptides [R_2_W_3_] **2a** and [R_2_W_4_] (**2b**) with two R residues exhibited a moderate activity against MRSA. The antimicrobial activity reduced from 32 μg/mL for peptide **2a** to 64 μg/mL for peptide **2b** with one extra W residue. Both peptides **2a** and **2b** showed negligible activity against all tested Gram-negative strains.

Peptides **3a**–**f** have three R residues. Peptides [R_3_W_3_] **3a** and [R_3_W_4_] (**3b**) showed a higher activity against MRSA compared to the corresponding peptides **2a** and **2b** composed of two R residues and showed MIC values of 16 and 8 μg/mL, respectively. The activity against Gram-negative bacteria was also slightly enhanced, especially against *E. coli* (25922), with an MIC value of 32 μg/mL for both **3a** and **3b**. However, the antimicrobial activity considerably decreased with increasing the number of W residues in peptides **3c**–**f** against all tested strains. The same pattern was also observed in peptides **5a**–**d**, **6a**–**d**, and **7a**–**d**, especially against MRSA (Table 1).

When l-Arg in [R_4_W_4_] (**1**) and [R_3_W_7_] (**3e**) was replaced with d-Arg in peptides [dR_3_W_7_] (**3f**) and [dR_4_W_4_] (**4a**), there was no difference in activity against *P. aeruginosa* and *E. coli*. However, [dR_4_W_4_] (**4a**) showed 2-fold less activity against MRSA and *K. pneumoniae* when compared with [R_4_W_4_] (**1**). [dR_3_W_7_] (**3e**) exhibited 4-fold more activity against MRSA but 4-fold less activity against *K. pneumoniae* when compared with [R_3_W_7_] (**3e**). These data indicated that the stereochemistry of the natural amino acids affects their activity in MRSA and *K. pneumoniae*.

Peptides [R_3_W_3_] (**3a**), [R_4_W_4_] (**1**), and [R_5_W_5_] (**5b**) that have an equal number of hydrophilic and hydrophobic residues showed an enhanced antimicrobial activity with increasing the recurring theme from 3 to 5 R/W (with a ring containing 6 to 10 amino acid residues). However, further expansion in the ring size (12–14 amino acid residues) in [R_6_W_6_] (**6c**) and [R_7_W_7_] (**7d**) led to a reduced antimicrobial activity.

Among all the peptides, [R_5_W_4_] (**5a**), [R_5_W_5_] (**5b**), [R_6_W_4_] (**6a**), and [R_6_W_5_] (**6b**) were found to have MIC values of 4–8, 32–64, 16–32, and 16–32 μg/mL against MRSA, *K. pneumoniae*, *P. aeruginosa*, and *E. coli*, respectively. Compound **5a** was two-fold more potent than [R_4_W_4_] (**1**) against *P. aeruginosa* (ATCC 27883) and had about two-fold higher HC_50_. Compounds **6a** and **6b** were 4-fold more potent than [R_4_W_4_] (**1**) against *P. aeruginosa* but two-fold less active against other bacteria. However, compounds **5a** and **5b** were 2-fold more potent than **1** against *P. aeruginosa* and had similar activity against other bacteria. In this case, increasing the number of R and W residues did not affect the antibacterial activity. However, the peptides with a lower number of W residues (**5a** vs **5b** and **6a** vs **6b**) showed less hemolytic activity. Among all the compounds, compound **5a** showed the highest therapeutic index (85.00) calculated for MRSA (Table 1)*.* Based on the highest therapeutic index values, we selected peptides **5a**, **5b**, **6a**, and **6b** for further studies.

#### 2.2.2 Broad-Spectrum ESKAPE Screening

ESKAPE pathogens (*Enterococcus faecium*, *Staphylococcus aureus*, *Klebsiella pneumoniae*, *Acinetobacter baumannii*, *Pseudomonas aeruginosa*, and *Enterobacter* species) are nosocomial pathogens that demonstrate multidrug resistance and virulence and are capable of escaping the action of antibiotics.^71^ The World Health Organization (WHO) has listed ESKAPE among the pathogens that urgently require new antibiotics.^72^ AMPs have broad-spectrum activity against a wide range of pathogens via physical damage of bacteria membranes, making it difficult for bacteria to develop resistance.^73^ Considering the priority of curing ESKAPE pathogens, AMPs could be a promising antibiotic alternative to fight against them. Numerous AMPs have been reported to exhibit promising antimicrobial potency against ESKAPE pathogens *in vitro* and *in vivo*.^74−77^

Based on the therapeutic index values of the peptide library (Table 1), we selected four promising peptides **5a**, **5b**, **6a**, and **6b** for screening against a broad range of antibiotic-resistant Gram-positive and Gram-negative bacteria representing the ESKAPE panel (Table 2). Daptomycin and polymyxin B were selected as positive controls against Gram-positive and Gram-negative bacteria, respectively. Generally, the results showed that the lead peptides demonstrated a promising antibacterial activity against Gram-positive bacteria and a moderate activity against Gram-negative strains.

**Table 2:** Minimum Inhibitory Concentrations of **5a**, **5b**, **6a**, and **6b** against ESKAPE Pathogens

	MIC (μg/mL)^a^					
bacterial strain	daptomycin	polymyxin B	**5a**	**5b**	**6a**	**6b**
*S*. *aureus* (ATCC 29213)^b^	1	ND^j^	4	4	8	8
*E*. *faecium* (ATCC 27270)^b^	4	ND	4	8	8	8
*E*. *faecium* (ATCC 700221)^b^^,^^c^	2	ND	4	8	4	16
*E*. *faecalis* (ATCC 29212)^b^	16	ND	8	16	16	16
*E*. *faecalis* (ATCC 51575)^b^^,^^d^	4	ND	16	16	32	16
*S*. *pneumonia* (ATCC 49619)^b^	4	ND	2	64	1	32
*S*. *pneumonia* (ATCC 51938)^b^^,^^e^	8	ND	2	64	2	32
*Bacillus subtilis* (ATCC-6633)^b^	0.5	ND	4	4	1	4
*Bacillus cereus* (ATCC-13061)^b^	2	ND	16	4	16	8
*E*. *coli* (ATCC BAA-2452)^f^^,^^g^	ND	1	16	16	16	32
*K*. *pneumoniae* (ATCC 13883)^f^	ND	1	32	64	64	64
*P*. *aeruginosa* (ATCC BAA-1744)^f^^,^^h^	ND	2	32	32	16	16
*P*. *aeruginosa* (ATCC 10145)^f^	ND	2	32	32	16	16
*Acinetobacter baumannii* (ATCCBAA-1605)^f^^,^^i^	ND	1	32	32	32	32

a The minimum inhibitory concentration (MIC) is the lowest concentration of the peptides that inhibited bacterial growth.

b Gram-positive strain.

c Vancomycin- and teicoplanin-resistant bacterial strain.

d Gentamicin-, streptomycin-, and vancomycin-resistant bacterial strain.

e Penicillin-, clindamycin-, cotrimoxazole-, and erythromycin-resistant bacterial strain.

f Gram-negative strain.

g Carbapenem, New Delhi metallo-beta-lactamase (NDM-1)-positive resistant bacterial strain (CRE (NDM-1)).

h Carbapenem-resistant bacterial strain.

i Resistant to ceftazidime, gentamicin, ticarcillin, piperacillin, aztreonam, cefepime, ciprofloxacin, imipenem, and meropenem.

j ND, not determined. The table presents the data of three independent experiments performed in triplicate.

Peptides **5a** and **6a** showed a significant antibacterial activity against Gram-positive multidrug-resistant strain *Streptococcus pneumoniae* (*S. pneumoniae*) (ATCC 51938) with an MIC value of 2 μg/mL, which was a 4-fold improvement when compared with daptomycin. Moreover, **5a** showed a significant antimicrobial activity against nonresistant strains *S. pneumoniae* (ATCC 49619) and *Enterococcus faecalis* (*E. faecalis*) (ATCC 29212) with MIC values of 2 and 8 μg/mL, respectively, which were 2-fold better than daptomycin. Furthermore, peptide **5a** exhibited a comparable activity against the nonresistant strain *E. faecium* (ATCC 27270) with an MIC value of 4 μg/mL when compared with daptomycin.

Peptide **6a** demonstrated an MIC value of 1 μg/mL and a 4-fold improvement against *S. pneumoniae* (ATCC 49619) when compared to daptomycin (MIC = 4 μg/mL). Compound **6a** also showed a promising antibacterial efficacy against *Bacillus subtilis* (ATCC 6633) with an MIC value of 1 μg/mL.

Peptides **5b**, **6a**, and **6b** showed a comparable antimicrobial activity with daptomycin against *E. faecalis* (ATCC 29212) with an MIC value of 16 μg/mL. However, peptides **5b** and **6b** showed a moderate antimicrobial activity against the multidrug-resistant strain *S. pneumoniae* (ATCC 51938) and nonresistant *S. pneumoniae* (49619) with MIC values of 64 and 32 μg/mL, respectively. Peptide **6a** displayed a moderate antimicrobial potency against multidrug-resistant *E. faecalis* (ATCC 51575) with an MIC of 32 μg/mL. Peptides **5b**, **6a**, and **6b** showed an antibacterial activity against the Gram-positive strains *S. aureus* (ATCC 29213), *E. faecium* (ATCC 27270), *E. faecium* (ATCC 700221), and *B. cereus* (ATCC 13061) with MICs of 4–16 μg/mL.

When evaluating the data against Gram-negative bacteria, peptides **5a**, **5b**, and **6a** displayed a promising antimicrobial activity with an MIC value of 16 μg/mL against the multidrug-resistant strain *E. coli* (ATCC BAA-2452). Furthermore, peptides **6a** and **6b** showed an MIC of 16 μg/mL against multidrug-resistant *P. aeruginosa* (ATCC BAA-1744) and the nonresistant strain *P. aeruginosa* (ATCC 10145). All tested peptides showed a moderate antibacterial activity against the rest of the tested Gram-negative bacterial strains (*K. pneumoniae* (ATCC 13883) and *Acinetobacter baumannii* (ATCC BAA-1605)) with MICs of 32–64 μg/mL, showing less activity than polymyxin B. Based on the broad-spectrum ESKAPE antibacterial screening results, lead peptides **5a** and **6a** were selected for further studies.

The activity of **5a** against additional ESKAPE strains, including clinical isolates, was examined by JMI Laboratories (North Liberty, IA) subcontracted by the National Institute of Health (Table 3). The 2021 SENTRY antimicrobial surveillance program provided the clinical isolates for the work conducted by JMI Laboratories (Table S2, Supporting Information). The data were consistent with those in Table 2. The activity of compound **5a** varied according to the bacterial species, with the highest activity detected against *S. aureus* and the lowest potency observed against *K. pneumoniae*. The *E. coli*, *P. aeruginosa*, and *S. aureus* sets were composed of 1 generally susceptible strain and 2 more resistant strains/isolates. For these small species sets, there was no evidence that the activity of the **5a** was impacted by the resistance phenotypes present.

**Table 3:** Minimum Inhibitory Concentration of **5a** against Additional ESKAPE Pathogens Including Clinical Isolates

	MIC (μg/mL)^a^	
bacterial strain	cefepime	**5a**
*S*. *aureus* (1193193)^b^	16	4
*S*. *aureus* (1195201)^c^	>32	4
*S*. *aureus* (ATCC 29213)^d^	2	4
*E*. *coli* (1191008)^e^	8	16
*E*. *coli* (ATCC 25922)^f^	0.06	16
*E*. *coli* (ATCC BAA-2452)^g^	32	16
*K*. *pneumoniae* (ATCC 700603)^h^	1	>64
*K*. *pneumoniae* (1188718)^i^	8	>64
*K*. *pneumoniae* (ATCC BAA 1705)^j^	32	>64
*P*. *aeruginosa* (1191191)^k^	32	32
*P*. *aeruginosa* (1188712)^k^	8	16
*P*. *aeruginosa* (ATCC 27853)^f^	1	32
*Acinetobacter baumannii* (NCTC^l^ 13304)^m^	32	16
*Acinetobacter baumannii-calcoaceticus* species complex (1188767)^n^	32	32
*Acinetobacter baumannii-calcoaceticus* species complex (1189854)^n^	32	16

a The minimum inhibitory concentration (MIC) is the lowest concentration of the peptides that inhibited bacterial growth.

b Methicillin-resistant *Staphylococcus aureus*.

c Multidrug-resistant *Staphylococcus aureus*.

d Methicillin-susceptible *Staphylococcus aureus*.

e Multidrug-resistant *E. coli*.

f Wild type.

g Carbapenem, New Delhi metallo-beta-lactamase (NDM-1)-positive resistant bacterial strain (CRE (NDM-1)).

h Extended-spectrum β-lactamase (ESBL) (*K. pneumoniae* ATCC 700603 produces SHV-18).

i Multidrug-resistant *K. pneumoniae*.

j Carbapenem-resistant Enterobacterales (KPC-2).

k Multidrug-resistant *P. aeruginosa*.

l National Collection of Type Cultures.

m Carbapenem-resistant *Acinetobacter baumannii* (CRAB) (OXA-27).

n Multidrug resistance.

#### 2.2.3 Minimum Bactericidal Concentration (MBC) Determination

For further determination of the antibacterial potential of lead peptides **5a**, **5b**, **6a**, and **6b**, we determined their minimum bactericidal concentration (MBC). MBC is the lowest concentration of an antibacterial agent required to kill 99.9% of the final inoculum after incubation over a fixed period (generally for 24 h) and is also known as the minimal lethal concentration (MLC).^78^ Four bacterial strains were used for MBC screening (Table 4) that include two multidrug-resistant strains (Gram-positive MRSA (ATCC BAA-1556) and Gram-negative *K. pneumoniae* (ATCC BAA-1705)) and two nonresistant strains (Gram-negative *P. aeruginosa* (ATCC 27883) and *E. coli* (ATCC 25922)). Meropenem, polymyxin B, daptomycin, and ciprofloxacin were used as control antibiotics. For better comparative analysis, we included conventional small-molecule antibiotics as well as standard lipopeptide antibiotics as positive controls.

**Table 4:** Minimum Bactericidal Concentrations (MBC) of Cyclic Peptides **5a**, **5b**, **6a**, and **6b**

		MBC (μg/mL)^a^			
peptide code	peptide sequence	MRSA^b^ (ATCC BAA-1556)	*K*. *pneumoniae*^c^ (ATCC BAA-1705)	*P*. *aeruginosa*^d^ (ATCC 27883)	*E*. *coli*^d^ (ATCC 25922)
**5a**	[R_5_W_4_]	8	64	32	16
**5b**	[R_5_W_5_]	16	32	32	64
**6a**	[R_6_W_4_]	16	128	16	32
**6b**	[R_6_W_5_]	16	64	32	128
**1**	[R_4_W_4_]	32	64	128	32
**Mero**	meropenem	8	64	4	2
**Dapto**	daptomycin	8	ND^e^	ND^e^	ND^e^
**Poly B**	polymyxin B	ND^e^	2	2	4
**Cipro**	ciprofloxacin	32	16	8	16

a The minimum bactericidal concentration (MBC) is the lowest concentration of the peptides that completely kill the bacteria*.*

b Methicillin-resistant bacterial strain.

c Imipenem-resistant bacterial strain.

d Nonresistant strain.

e ND = not determined. The table presents the data of three independent experiments performed in triplicate.

Peptide **5a** showed bactericidal activity with an MBC value of 8 μg/mL against MRSA (ATCC BAA-1556), comparable to meropenem. On the other hand, peptides **5a** and **6b** displayed an MBC value of 64 μg/mL against *K. pneumoniae* (ATCC BAA-1705) comparable to meropenem. Peptide **5b** killed bacteria at 32 μg/mL with a 2-fold improvement compared to meropenem against *K. pneumoniae* (ATCC BAA-1705). Peptides **5b**, **6a**, and **6b** demonstrated MBCs of 16 μg/mL against MRSA (ATCC BAA-1556), which was 2-fold better than the parent peptide [R_4_W_4_]. All tested peptides showed reduced MBC values against *P. aeruginosa* (ATCC 27883) with 4–8-fold enhancement in activity compared to [R_4_W_4_]. However, peptide **6a** showed the highest bactericidal potency against *P. aeruginosa* (ATCC 27883) with an 8-fold enhancement in activity compared to [R_4_W_4_]. Peptide **5a** demonstrated an MBC of 16 μg/mL against *E. coli* (ATCC 25922) with a 2-fold enhancement in activity compared to [R_4_W_4_]. In contrast, **6a** showed a bactericidal activity at 32 μg/mL, comparable to [R_4_W_4_] against *E. coli* (ATCC 25922). Based on these results, peptides **5a** and **6a** were considered as the lead peptides for further studies.

### 2.3 Salt Sensitivity

Prior studies have indicated that positive charges facilitate antimicrobial peptide binding to the negatively charged bacterial membranes through electrostatic interactions.^79^ However, the presence of other positively charged entities, like salts, can weaken this electrostatic interaction.^80^ Thus, the antibacterial activities of the lead peptides **5a** and **6a** were examined in the presence of different physiological salts at physiologic concentrations and fetal bovine serum compared with media only (Table S3, Supporting Information). Peptides **5a** and **6a** retained their antimicrobial potency in the presence of monovalent (Na^+^ and K^+^), divalent (Ca^2+^), and trivalent (Fe^3+^) at physiological concentrations, suggesting that the cationic character had no effect on the peptide’s antimicrobial activity, presumably due to the presence of the bulky side chain of W that could enhance the association of the AMP with the bacterial membrane,^80,81^ contributing to the antibacterial effect in the presence of salts. However, there was a slight increase in the antibacterial activity of **5a** and **6a** by 2-fold against all tested strains in the presence of NH_4_^+^ and Mg^2+^. Peptides **5a** and **6a** showed MICs of 2 and 4 μg/mL against MRSA, respectively, in the presence of NH_4_^+^ and Mg^2+^. Peptides **5a** and **6a** showed an antibacterial activity against Gram-negative strains *P. aeruginosa* and *E. coli* in the presence of NH_4_^+^ and Mg^2+^ with MICs of 8–16 μg/mL. However, **6a** exhibited a moderate activity against *K. pneumoniae* (ATCC BAA-1705) with an MIC value of 32 μg/mL. Moreover, peptide **5a** showed a 2-fold enhancement in antibacterial efficacy against *K. pneumoniae* (ATCC BAA-1705) with an MIC of 16 μg/mL in the presence of divalent Ca^2+^.

### 2.4 Combination Therapy

Multidrug-resistant bacteria are considered a major threat to hospitalized patients and have been associated with high mortality rates, especially those caused by Gram-negative pathogens*.* The spread and danger of multidrug-resistant infection force the medical community to rely on additional broad-spectrum antibiotics to cure these infections, leading to more resistance.^82^ This has become a concerning issue because of the limited options of antimicrobial agents currently available to fight against these pathogens.^83−85^ The lack of effective antibiotic options in the market is because of slow pace, high cost, and low selling prices of new antibiotics.^86^ Combination therapy can deal with and overcome antimicrobial resistance and repurpose existing antibiotics.^87^ Thus, designing antimicrobial combination therapies to identify novel synergistic drug interactions is a promising approach to fight against or delay bacterial resistance.

Recently, the scientific community has paid much attention to AMPs as promising alternatives to antibiotics, especially against multidrug-resistant bacteria due to their broad-spectrum antibacterial activity and unique nonspecific membrane rupture mechanism, which prevents or retards the ability of bacteria to develop resistance.^17^ Due to their potency as antimicrobial agents, many AMPs have been subjected to clinical trials.^88^

Multiple AMPs are released during immune responses *in vivo* to fight bacterial infections, suggesting that the combinations that have synergistic interactions could be more lethal. Moreover, previous reports indicated that using antibiotic combinations supports antibacterial efficacy and aids in preventing bacterial resistance.^89,90^ The use of AMPs along with commercially available antibiotics has been proposed as an alternative option leading to antibiotic revival. Prior studies indicated that AMPs act in synergy with traditional antibiotics against multidrug-resistant bacteria.^91−93^ Based on the promising antibacterial activities of **5a** and **6a** as AMPs, we investigated whether they can be used in combination with other antibiotics.

#### 2.4.1 Antibacterial Evaluation of Peptides and Antibiotic Physical Mixtures

The physical mixtures of a wide range of frontline antibiotics with lead peptides (**5a** and **6a**; 1:1 w/w) were screened against four bacterial strains. Notably, as compared to antibiotics alone, a significant increase in activity was observed for all the tested peptide antibiotic physical mixtures (Table S4, Supporting Information). Significant enhancement in activity was observed for the combination of **5a** with kanamycin showing MICs of 4 and 8 μg/mL against MRSA (ATCC BAA 1556) and *P. aeruginosa* (ATCC 27883), respectively, and demonstrating a 64- and 32-fold enhancement, respectively, when compared with kanamycin alone*.* A combination of **5a** with polymyxin B showed an MIC value of 2 μg/mL against MRSA with a 32-fold improvement versus polymyxin B alone.

Vancomycin and daptomycin are peptide-based antibiotics used for the treatment of Gram-positive infections. Vancomycin is a frontline glycopeptide, and daptomycin is a cyclic lipopeptide antibiotic. Gram-negative pathogens have an additional outer membrane that coats the cell surface. This membrane is highly resistant and impermeable to vancomycin and daptomycin, which limits their access to Gram-negative pathogens and makes them least susceptible to vancomycin and daptomycin compared with Gram-positive ones.^94,95^

Interestingly, although vancomycin and daptomycin are active against Gram-positive bacteria exclusively, the physical mixture of peptide **5a** with vancomycin and daptomycin showed a significant activity against Gram-negative bacteria. The vancomycin combination showed MICs of 4, 8, and 8 μg/mL against *K. pneumoniae* (ATCC BAA-1705), *P. aeruginosa* (ATCC 27883), and *E. coli* (ATCC 25922), respectively, while the daptomycin combination demonstrated MIC values of 4–16 μg/mL against the same strains. Both vancomycin and daptomycin in combination with **5a** displayed a 32–128-fold enhancement in the antibacterial efficacy against Gram-negative strains compared with antibiotics alone.

Another interesting result was observed for combining clindamycin with **5a**, which showed 256- and 128-fold enhancement in activity against *K. pneumoniae* (ATCC BAA-1705) and *P. aeruginosa* (ATCC 27883), respectively, with MIC values of 2–4 μg/mL. A combination of clindamycin with **5a** also showed a 16-fold enhancement in activity against *E. coli* (ATCC 25922) when compared with clindamycin alone.

Combinations of ciprofloxacin and metronidazole with **5a** showed a 32-fold enhancement in activity against *K. pneumoniae* (ATCC BAA-1705) with an MIC value of 8 μg/mL, while the metronidazole combination with **5a** displayed an MIC value of 2 μg/mL against MRSA, showing a 16-fold improvement when compared with metronidazole alone. Similarly, a 16-fold enhancement in activity was observed for a combination of tetracycline with **5a** (MIC value of 2 μg/mL) when compared with tetracycline alone against *P. aeruginosa* (ATCC 27883).

In combination with **5a**, kanamycin, metronidazole, ciprofloxacin, levofloxacin, and tetracycline showed a remarkable 8-fold enhancement in activity against *E. coli* (ATCC 25922) (MICs in the range of 1–16 μg/mL) as compared to the antibiotics alone. On the other hand, against *K. pneumoniae* (ATCC BAA-1705), tetracycline, levofloxacin, kanamycin, and meropenem demonstrated an 8-fold improvement in the antibacterial activity (MIC values of 2–8 μg/mL) in combination with **5a** versus the antibiotics alone. The combination of ciprofloxacin with **5a** displayed an 8- and 4-fold improvement, respectively, against MRSA and *P. aeruginosa* (ATCC 27883), when compared with the antibiotic alone, with MIC values of 2 and 0.125 μg/mL. A combination of meropenem and **5a** also showed an 8-fold enhancement in activity against *P. aeruginosa* when compared with meropenem. The rest of the combinations showed improvement in the antibacterial efficacy of the tested antibiotics with a 2–4-fold enhancement in activity against tested strains*.*

The physical mixture of peptide **6a** with antibiotics also showed enhancement in activity against all the tested strains (Table S5, Supporting Information). Significant enhancement in activity (64-fold) was observed for a combination of **6a** and clindamycin with an MIC value of 8 μg/mL against *K. pneumoniae* (BAA-1705) and *P. aeruginosa* (ATCC 27883), when compared with clindamycin alone. A remarkable enhancement in antibacterial activity was also observed for the combination of **6a** with kanamycin (64-fold against MRSA and *P. aeruginosa* (ATCC 27883) (MIC of 4 μg/mL)) as compared to kanamycin alone.

Similar to **5a**, the physical mixture of peptide **6a** with vancomycin and daptomycin showed significant improvement in activity against Gram-negative bacteria. The vancomycin combination showed MIC values of 8–16 μg/mL with a 16–32-fold improvement against *K. pneumoniae* (ATCC BAA-1705), *E. coli* (ATCC 25922), and *P. aeruginosa* (ATCC 27883) when compared with vancomycin alone. The daptomycin combination demonstrates MIC values of 8–16 μg/mL against the same strains with a 32–64-fold enhancement versus daptomycin alone.

A combination of **6a** with metronidazole or polymyxin B displayed a 16-fold improvement against MRSA with MICs of 2 and 4 μg/mL, respectively. Moreover, a combination of **6a** with meropenem demonstrated a 15-fold enhancement in activity against *E. coli* 25922 with an MIC value of 0.065 μg/mL when compared with meropenem alone. Furthermore, the metronidazole and ciprofloxacin physical mixture with peptide **6a** showed an MIC value of 16 μg/mL and a 16-fold improvement in activity against *K. pneumoniae* (ATCC BAA-1705) when compared with parent antibiotics alone.

In a similar trend, kanamycin, metronidazole, ciprofloxacin, levofloxacin, and clindamycin showed an 8-fold enhancement in activity compared to the antibiotics alone against *E. coli* (ATCC 25922) when combined with **6a** (MIC range of 4–16 μg/mL). On the other hand, the combination of tetracycline, metronidazole, and meropenem with **6a** demonstrated an 8-fold improvement in the antibacterial activity compared to the parent antibiotics alone, with MIC values of 4, 4, and 0.125 μg/mL, respectively, against *P. aeruginosa* (ATCC 27883). A combination of kanamycin with **6a** displayed an 8-fold improvement with MIC values of 8 μg/mL against *K. pneumoniae* (ATCC BAA-1705). The rest of the combinations showed improvement in the antibacterial efficacy of the tested antibiotics with a 2–4-fold enhancement in activity against tested strains.

The nature of improvement in activity in combination is not clear at this time. Considering the remarkable membrane disruption potential of lead cyclic peptides as indicated, it seems that the peptide compromises the membrane barrier, which eventually facilitates the translocation of the antibiotics inside the cell and exerts cell death. Moreover, a combination of the peptide and antibiotics might have generated a unique complex via various chemical interactions that could have a distinct antibacterial mechanism. Further studies are required to determine the nature of the antibacterial enhancement of the activity of a combination versus the parent analogs.

#### 2.4.2 Synergistic Studies

Based on the promising antibacterial enhancement results observed for the physical mixture of the lead peptides **5a** and **6a** in combination with various antibiotics, we conducted a more extensive synergistic study on the physical mixture combinations. The checkerboard assay was performed to study the synergistic interaction between lead peptides (**5a** and **6a**) and 11 commercially available antibiotics in combinations. This study aimed to compare the influence of synergistic combinations on the antibacterial efficacy against the individual peptide or antibiotic. The comparison is represented by the fractional inhibitory concentration index (FICI) value, which classifies the combined impact of the two tested compounds. Selected antibiotics were tested as 11 dose points of two-fold serial dilutions across the assay plate in combination with seven points of two-fold serial dilution of the peptides down the assay plate. Two-fold serial dilutions for antibiotics and peptides were performed individually to determine the MIC value for each tested compound.

Table 5 depicts the data obtained from the checkerboard assay for peptide **5a** along with 11 commercial antibiotics to determine the ideal concentration of both the peptide and antibiotics to afford an optimal antibacterial activity in combination. The concentrations of peptide **5a** started from its MIC and were then subjected to two-fold serial dilutions (a total of 7 points).

**Table 5:** The Synergistic Effect of the Peptide **5a**/Antibiotic Combination

		MIC (μg/mL)				
combination	bacterial strain	antibiotic in combination	antibiotic alone	FIC antibiotic	FICI^b^	integrative category
[R_5_W_4_] + tetracycline	*S*. *aureus* (ATCC BAA-1556)	0.0625	0.250	0.25	0.500	synergy
	*P*. *aeruginosa* (ATCC 27883)	4.00	32.0	0.125	0.375	synergy
	*E*. *coli* (ATCC 25922)	1.00	8.00	0.125	0.375	synergy
	*K*. *pneumoniae* (ATCC BAA-1705)	2.00	16.0	0.125	0.375	synergy
[R_5_W_4_] + tobramycin	*S*. *aureus* (ATCC BAA-1556)	0.125	0.500	0.250	0.500	synergy
	*P*. *aeruginosa* (ATCC 27883)	0.125	0.500	0.250	0.500	synergy
	*E*. *coli* (ATCC 25922)	2.00	8.00	0.250	0.500	synergy
	*K*. *pneumoniae* (ATCC BAA-1705)	2.00	16.0	0.125	0.375	synergy
[R_5_W_4_] + clindamycin	*S*. *aureus* (ATCC BAA-1556)	0.0312	0.125	0.249	0.499	synergy
	*P*. *aeruginosa* (ATCC 27883)	8.00	512	0.016	0.266	synergy
	*E*. *coli* (ATCC 25922)	4.00	64.0	0.063	0.313	synergy
	*K*. *pneumoniae* (ATCC BAA-1705)	2.00	512	0.0039	0.254	synergy
[R_5_W_4_] + kanamycin	*S*. *aureus* (ATCC BAA-1556)	ND^a^	256	ND^a^	ND^a^	ND^a^
	*P*. *aeruginosa* (ATCC 27883)	8.00	256	0.0313	0.281	synergy
	*E*. *coli* (ATCC 25922)	8.00	32.0	0.250	0.500	synergy
	*K*. *pneumoniae* (ATCC BAA-1705)	8.00	64.0	0.125	0.375	synergy
[R_5_W_4_] + levofloxacin	*S*. *aureus* (ATCC BAA-1556)	1.00	4.00	0.25	0.500	synergy
	*P*. *aeruginosa* (ATCC 27883)	0.125	1.00	0.125	0.375	synergy
	*E*. *coli* (ATCC 25922)	8.00	64.0	0.125	0.375	synergy
	*K*. *pneumoniae* (ATCC BAA-1705)	8.00	64.0	0.125	0.375	synergy
[R_5_W_4_] + ciprofloxacin	*S*. *aureus* (ATCC BAA-1556)	2.00	16.0	0.125	0.625^c^	partial synergy
	*P*. *aeruginosa* (ATCC 27883)	0.125	0.500	0.250	0.500	synergy
	*E*. *coli* (ATCC 25922)	ND^a^	64.0	ND^a^	ND^a^	ND^a^
	*K*. *pneumoniae* (ATCC BAA-1705)	16.0	256	0.0625	0.313	synergy
[R_5_W_4_] + polymyxin B	*S*. *aureus* (ATCC BAA-1556)	2.00	64.0	0.0313	0.531^c^	partial synergy
	*P*. *aeruginosa* (ATCC 27883)	0.125	1.00	0.125	0.375	synergy
	*E*. *coli* (ATCC 25922)	0.25	2.00	0.125	0.375	synergy
	*K*. *pneumoniae* (ATCC BAA-1705)	0.125	1.00	0.125	0.375	synergy
[R_5_W_4_] + metronidazole	*S*. *aureus* (ATCC BAA-1556)	2.00	32.0	0.063	0.563^c^	partial synergy
	*P*. *aeruginosa* (ATCC 27883)	8.00	32.0	0.250	0.500	synergy
	*E*. *coli* (ATCC 25922)	ND^a^	128	ND^a^	ND^a^	ND^a^
	*K*. *pneumoniae* (ATCC BAA-1705)	8.00	256	0.0313	0.281	synergy
[R_5_W_4_] + meropenem	*S*. *aureus* (ATCC BAA-1556)	ND^a^	2.00	ND^a^	ND^a^	ND^a^
	*P*. *aeruginosa* (ATCC 27883)	0.125	1.00	0.125	0.375	synergy
	*E*. *coli* (ATCC 25922)	ND^a^	1.00	ND^a^	ND^a^	ND^a^
	*K*. *pneumoniae* (ATCC BAA-1705)	4.00	16.0	0.250	0.500	synergy
[R_5_W_4_] + vancomycin	*S*. *aureus* (ATCC BAA-1556)	0.500	1.00	0.500	0.750	partial synergy
	*P*. *aeruginosa* (ATCC 27883)	16.0	256	0.0630	0.313	synergy
	*E*. *coli* (ATCC 25922)	8.00	256	0.0313	0.531^c^	partial synergy
	*K*. *pneumoniae* (ATCC BAA-1705)	32.0	512	0.0625	0.563^c^	partial synergy
[R_5_W_4_] + daptomycin	*S*. *aureus* (ATCC BAA-1556)	ND^a^	2.00	ND^a^	ND^a^	ND^a^
	*P*. *aeruginosa* (ATCC 27883)	32.0	512	0.0630	0.313	synergy
	*E*. *coli* (ATCC 25922)	ND^a^	>128	ND^a^	ND^a^	ND^a^
	*K*. *pneumoniae* (ATCC BAA-1705)	8.00	512	0.0156	0.516^c^	partial synergy

a ND, not determined.

b FIC of the peptide in combination is 0.25 (peptide concentration equivalent to one-fourth of its MIC).

c FIC of the peptide in combination is 0.5 (peptide concentration equivalent to one-half of its MIC); all experiments were performed in triplicate.

Tetracycline, tobramycin, clindamycin, kanamycin, levofloxacin, polymyxin B, metronidazole, and vancomycin had remarkable improvement in antimicrobial activity in combination with the **5a** concentration equivalent to one-fourth of its MIC (4-fold improvement, FIC_peptide_ = 0.25). A summary of the results is described here.

The clindamycin combination showed significant enhancement against tested Gram-negative strains. Clindamycin displayed 256-fold reduced MIC values compared to individual antibiotics against clinically resistant *K. pneumoniae* (ATCC BAA-1705) with an FICI value of 0.254. Clindamycin demonstrated a 64- and 16-fold improvement and FICI values of 0.266 and 0.313 against *P. aeruginosa* (27883) and *E. coli* (ATCC 25922), respectively. Clindamycin, ciprofloxacin, and metronidazole showed significant improvement against clinically resistant *K. pneumoniae* (ATCC BAA-1705) with FICI values of 0.254, 0.313, and 0.281, showing 256, 16, 32-fold improved MIC values compared to individual antibiotics, respectively. Moreover, kanamycin, vancomycin, and daptomycin displayed significant enhancement in activity against *P. aeruginosa* (ATCC 27883) with a 32-, 16-, and 16-fold enhancement in antibiotic activity and FICI values of 0.281, 0.313, and 0.313, respectively.

Tetracycline, tobramycin, kanamycin, levofloxacin, and polymyxin B showed an 8-fold enhancement in antibiotic activity with a FICI value of 0.375 against *K. pneumoniae* (ATCC BAA-1705), while meropenem showed a 4-fold improvement with an FICI of 0.5. Tetracycline, levofloxacin, and polymyxin showed an 8-fold improvement against *E. coli* (ATCC 25922) with an FICI of 0.375, while a 4-fold enhancement and an FICI of 0.5 were observed for tobramycin and kanamycin.

For the *P. aeruginosa* (ATCC 27883) strain, tetracycline, levofloxacin, polymyxin B, and meropenem demonstrated an 8-fold improvement with an FICI value of 0.375, while tobramycin, ciprofloxacin, and metronidazole showed a 4-fold enhancement. Furthermore, a 4-fold improvement was observed for each of tetracycline, tobramycin, clindamycin, and levofloxacin against multidrug-resistant Gram-positive MRSA strains.

Some antibiotics showed partial synergy due to the minor improvement for the peptide MIC in combination despite the remarkable improvement in some of the tested antibiotics’ efficacy (peptide concentration equivalent to one-half of its MIC with FICI = 0.5- and 2-fold improvement). For example, ciprofloxacin, polymyxin B, metronidazole, and vancomycin displayed a 2–32 improvement of antibiotic MICs and partial synergy in combination against MRSA. In the same way, despite the significant improvement in antibiotic activity with a 16–32 improvement in MICs, vancomycin demonstrated partial synergy against *E. coli* (ATCC 25922) and *K. pneumoniae* (ATCC BAA-1705) with FICI values of 0.531 and 0.563, respectively. Daptomycin showed partial synergistic interactions in combination with **5a** with an FICI value of 0.516 and 64-fold reduced MIC values compared to the parent antibiotic against *K. pneumoniae* (ATCC BAA-1705).

Checkerboard assay results for peptide **6a** in combination with the antibiotics are presented in Table 6. In combination with **6a** at a concentration equivalent to one-fourth of its MIC (4-fold improvement, FIC_peptide_ = 0.25), clindamycin, kanamycin, ciprofloxacin, polymyxin B, tetracycline, and levofloxacin exhibited significant improvement in the antimicrobial activity (Table 6). A summary of the results is described here.

**Table 6:** The Synergistic Effect of the Peptide **6a**/Antibiotic Combination

		MIC (μg/mL)				
combination	bacterial strain	antibiotic in combination	antibiotic alone	FIC antibiotic	FICI^b^	integrative category
[R_6_W_4_] + tetracycline	*S*. *aureus* (ATCC BAA-1556)	0.0625	0.250	0.250	0.500	synergy
	*P*. *aeruginosa* (ATCC 27883)	8.00	32.0	0.250	0.500	synergy
	*E*. *coli* (ATCC 25922)	0.500	8.00	0.063	0.313	synergy
	*K*. *pneumoniae* (ATCC BAA-1705)	2.00	16.0	0.125	0.375	synergy
[R_6_W_4_] + tobramycin	*S*. *aureus* (ATCC BAA-1556)	0.0625	0.500	0.125	0.375	synergy
	*P*. *aeruginosa* (ATCC 27883)	ND^a^	0.500	ND^a^	ND^a^	ND^a^
	*E*. *coli* (ATCC 25922)	ND^a^	8.00	ND^a^	ND^a^	ND^a^
	*K*. *pneumoniae* (ATCC BAA-1705)	4.00	16.0	0.250	0.500	synergy
[R_6_W_4_] + clindamycin	*S*. *aureus* (ATCC BAA-1556)	0.031	0.125	0.248	0.498	synergy
	*P*. *aeruginosa* (ATCC 27883)	8.00	512	0.0160	0.516^c^	synergy
	*E*. *coli* (ATCC 25922)	4.00	64.0	0.063	0.313	synergy
	*K*. *pneumoniae* (ATCC BAA-1705)	8.00	512	0.0160	0.266	synergy
[R_6_W_4_] + kanamycin	*S*. *aureus* (ATCC BAA-1556)	4.00	256	0.0156	0.516^c^	partial synergy
	*P*. *aeruginosa* (ATCC 27883)	4.00	256	0.0156	0.266	synergy
	*E*. *coli* (ATCC 25922)	4.00	32.0	0.125	0.375	synergy
	*K*. *pneumoniae* (ATCC BAA-1705)	16.0	64.0	0.250	0.500	synergy
[R_6_W_4_] + levofloxacin	*S*. *aureus* (ATCC BAA-1556)	ND^a^	4.00	ND^a^	ND^a^	ND^a^
	*P*. *aeruginosa* (ATCC 27883)	0.125	1.00	0.125	0.375	synergy
	*E*. *coli* (ATCC 25922)	8.00	64.0	0.125	0.375	synergy
	*K*. *pneumoniae* (ATCC BAA-1705)	8.00	64.0	0.125	0.375	synergy
[R_6_W_4_] + ciprofloxacin	*S*. *aureus* (ATCC BAA-1556)	4.00	16.0	0.250	0.750 ^*c*^	partial synergy
	*P*. *aeruginosa* (ATCC 27883)	0.125	0.500	0.250	0.500	synergy
	*E*. *coli* (ATCC 25922)	8.00	64.0	0.125	0.375	synergy
	*K*. *pneumoniae* (ATCC BAA-1705)	16.0	256	0.0625	0.313	synergy
[R_6_W_4_] + polymyxin B	*S*. *aureus* (ATCC BAA-1556)	4	64.0	0.063	0.563^c^	partial synergy
	*P*. *aeruginosa* (ATCC 27883)	ND^a^	1.00	ND^a^	ND^a^	ND^a^
	*E*. *coli* (ATCC 25922)	0.125	2.00	0.063	0.313	synergy
	*K*. *pneumoniae* (ATCC BAA-1705)	0.031	1.00	0.031	0.281	synergy
[R_6_W_4_] + metronidazole	*S*. *aureus* (ATCC BAA-1556)	2.00	32.0	0.063	0.563^c^	partial synergy
	*P*. *aeruginosa* (ATCC 27883)	4.00	32.0	0.125	0.375	synergy
	*E*. *coli* (ATCC 25922)	16.0	128	0.125	0.625^c^	partial synergy
	*K*. *pneumoniae* (ATCC BAA-1705)	16.0	256	0.063	0.313	synergy
[R_6_W_4_] + meropenem	*S*. *aureus* (ATCC BAA-1556)	ND^a^	2.00	ND^a^	ND^a^	ND^a^
	*P*. *aeruginosa* (ATCC 27883)	0.250	1.00	0.250	0.500	synergy
	*E*. *coli* (ATCC 25922)	0.125	1.00	0.125	0.375	synergy
	*K*. *pneumoniae* (ATCC BAA-1705)	ND^a^	16.0	ND^a^	ND^a^	ND^a^
[R_6_W_4_] + vancomycin	*S*. *aureus* (ATCC BAA-1556)	0.250	1.00	0.250	0.500	synergy
	*P*. *aeruginosa* (ATCC 27883)	8.00	256	0.0313	0.531^c^	partial synergy
	*E*. *coli* (ATCC 25922)	16.0	256	0.0630	0.563^c^	partial synergy
	*K*. *pneumoniae* (ATCC BAA-1705)	32.0	512	0.0625	0.313	synergy
[R_6_W_4_] + daptomycin	*S*. *aureus* (ATCC BAA-1556)	ND^a^	2.00	ND^a^	ND^a^	ND^a^
	*P*. *aeruginosa* (ATCC 27883)	8.00	512	0.0156	0.516^c^	partial synergy
	*E*. *coli* (ATCC 25922)	ND^a^	>128	ND^a^	ND^a^	ND^a^
	*K*. *pneumoniae* (ATCC BAA-1705)	64.0	512	0.125	0.375	synergy

a ND, not determined.

b FIC of the peptide in combination is 0.25 (peptide concentration equivalent to one-fourth of its MIC).

c FIC of the peptide in combination is 0.5 (peptide concentration equivalent to one-half of its MIC); all experiments were performed in triplicate.

The clindamycin combination with **6a** showed significant enhancement in activity against *E. coli* (ATCC 25922) and *K. pneumoniae* (ATCC BAA-1705). Clindamycin demonstrated a 64-fold improvement in MIC values when combined with **6a** compared to antibiotics alone against clinically resistant *K. pneumoniae* (ATCC BAA-1705) with an FICI value of 0.266 while displaying a 16-fold MIC improvement versus clindamycin alone with an FICI value of 0.313 against *E. coli* (ATCC 25922).

Moreover, for polymyxin B in combination with **6a**, a 32-fold (FICI = 0.281) and 16-fold (FICI = 0.313) improvement in MIC against *K. pneumoniae* (ATCC BAA-1705) and *E. coli* (ATCC 25922), respectively, was observed when compared with the antibiotic alone. Kanamycin displayed a 64-fold MIC improvement with an FICI value of 0.266 against *P. aeruginosa* (ATCC 27883). Ciprofloxacin, metronidazole, and vancomycin showed remarkable improvement when combined with **6a** against clinically resistant *K. pneumoniae* (ATCC BAA-1705) with a 16-fold improvement in MIC values compared to the parent antibiotics and FICI values of 0.313. Tetracycline showed a 16-fold enhancement in activity against *E. coli* (ATCC 25922) with an FICI value of 0.313. Tobramycin displayed an 8-fold improvement in antibiotic activity compared with the antibiotic alone, with an FICI value of 0.375 against the clinically resistant Gram-positive MRSA strain.

Ciprofloxacin, kanamycin, levofloxacin, and meropenem showed an 8-fold enhancement in antibiotic activity when compared with the parent antibiotic and with an FICI value of 0.375 against *E. coli* (ATCC 25922). Tetracycline, levofloxacin, and daptomycin in combination with **6a** showed an 8-fold improvement against *K. pneumoniae* (ATCC BAA-1705) compared with the antibiotics alone with an FICI of 0.375, while a 4-fold enhancement in activity and an FICI of 0.5 were observed for tobramycin and kanamycin.

For *P. aeruginosa* (ATCC 27883), levofloxacin and metronidazole demonstrated an 8-fold improvement versus antibiotics alone with an FICI value of 0.375, while tetracycline, ciprofloxacin, and meropenem showed a 4-fold enhancement in activity and an FICI of 0.5. An about 4-fold improvement was observed for tetracycline, clindamycin, and vancomycin against the multidrug-resistant Gram-positive MRSA strain compared with the antibiotics alone.

Partial synergy was observed for some of the tested antibiotics in combination with **6a** at one-half of the peptide MIC concentration with FIC = 0.5- and 2-fold improvement. Some antibiotics displayed a 4–64-fold enhancement in activity of antibiotic MICs and partial synergy in combination. Kanamycin, ciprofloxacin, polymyxin B, and metronidazole showed partial synergistic interactions with peptide **6a** in combination with FICI values of 0.516, 0.750, 0.563, and 0.563 against MRSA, respectively.

Despite the significant improvement in antibiotic activity with a 32–64 improvement in antibiotics’ MICs of clindamycin, vancomycin, and daptomycin, they demonstrated partial synergy against *P. aeruginosa* (ATCC 27883) with FICI values of 0.516, 0.531, and 0.516, respectively. Vancomycin and metronidazole showed partial synergistic interactions with **6a** against *E. coli* (ATCC 25922) with FICI values of 0.563 and 0.625 and 8–16-fold reduced MIC values compared to individual antibiotics.

### 2.5 Cytotoxicity Studies

#### 2.5.1 Hemolytic Activity

The cytotoxicity of all the synthesized cyclic peptides (**2a**–**7d**) was examined by conducting a hemolytic assay using human red blood cells (hRBCs). In general, peptides having a higher number of W residues as compared to R residues displayed high toxicity, as summarized in Table 1. For instance, **2b**, composed of three W, was found to be more toxic than **2a**, which has two W. Similarly, among the peptides composed of three (**3a**–**3e**), four (**4a**–**4c**), five (**5a**–**5e**), six (**6a**–**6d**), and seven (**7a**–**7d**) R and a varying number of W, the maximum toxicity was observed for peptides having a low cationic charge/hydrophobic ratio (**2a**, **2b**, **3b**, **3c**, **4a**–**4c**, **5c**, **5d**, **6c**, and **6d**). Thus, it seems that as the hydrophobic bulk increases, the ability of the peptide to discriminate between the anionic bacterial surface and the zwitterionic mammalian membrane decreases. These outcomes are in agreement with many previous reports,^96,97^ including ours. Furthermore, we calculated the therapeutic index for MRSA cells by dividing HC_50_ values with the MICs against MRSA (Table 1). The maximum therapeutic index was observed for **5a**, **5b**, **6a**, and **6b** with values of 85.0, 57.5, 33.1, and 31.9, respectively.

#### 2.5.2 Cell Viability Assay

**Figure 2:**
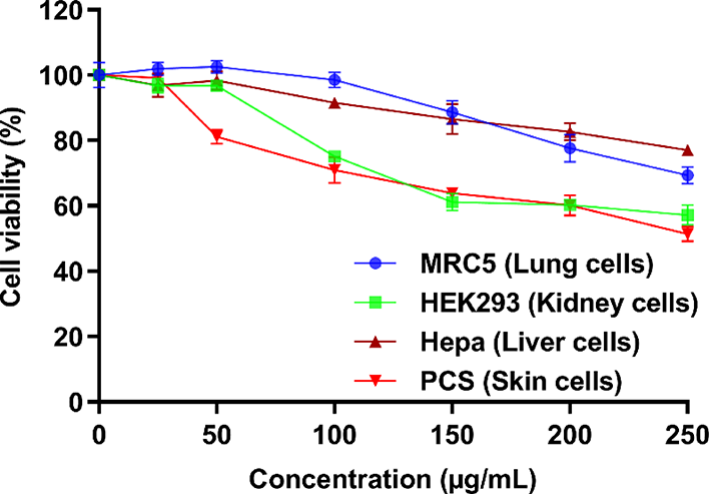
Cytotoxicity assay of the lead cyclic peptide **5a**. The results represent the data obtained from the experiments performed in triplicate (incubation for 24 h). DMSO (30%) was used as a positive control. The cells treated with DMSO (30%) showed 7–11% cell viability compared to nontreated (NT) cells having 100% cell viability.

To further examine the toxicity of the lead peptide **5a**, we conducted cytotoxicity screening against normal lung (MRC-5), kidney (HEK-293), liver (HepaRG), and human skin fibroblast cells (HeKa) cells (Figure 2). At a 100 μg/mL concentration, **5a** showed negligible cytotoxicity against lung (98% cell viability) and liver cells (91% cell viability). However, comparatively higher cytotoxicity was observed against kidney and skin cells with 75 and 70% cell viability, respectively. Quite a similar trend of cytotoxicity was observed at the highest experimental concentration of 250 μg/mL with minimal cytotoxicity against liver (77% cell viability) and lung cells (69% cell viability) and comparatively low cell viability for kidney (57% cell viability) and skin cells (51% cell viability). The therapeutic index was calculated based on HC_50_/MIC against MRSA in Table 1. If we calculate the therapeutic index based on the cytotoxicity assay in skin cells versus MIC against MRSA, the TI will be approximately 63. However, the therapeutic index values will be much higher for lung, kidney, and liver cells since cell viability is much higher even at 250 μg/mL. Overall, like hemolytic data, the cytotoxicity results also revealed a good selectivity profile of **5a** since minimal cytotoxicity was observed at MIC values.

### 2.6 Bactericidal Kinetic Assay

**Figure 3:**
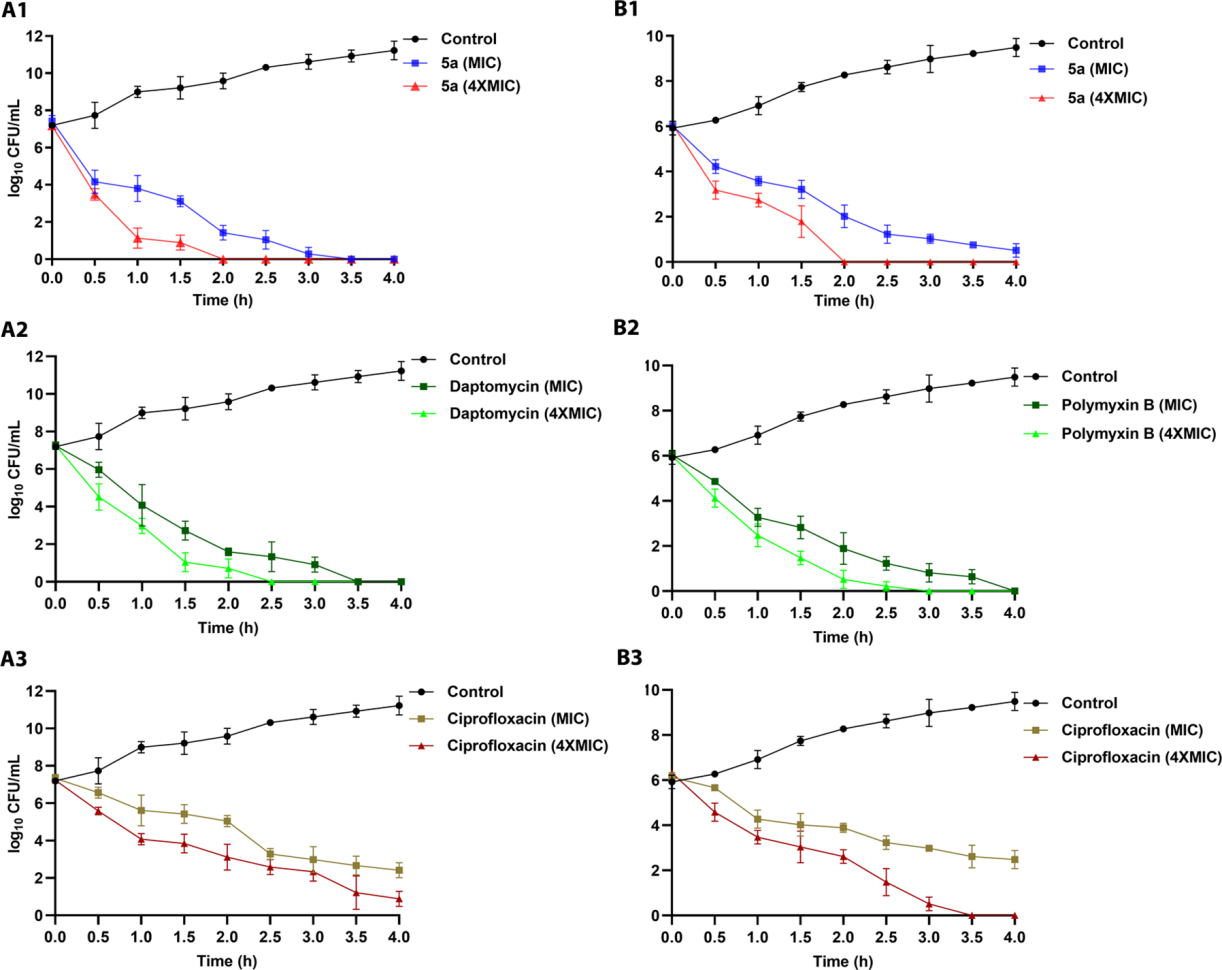
Bactericidal kinetics of the lead cyclic peptide (**5a**) and standard antibiotics (daptomycin, polymyxin B, and ciprofloxacin) against MRSA (A1–A3) and *E. coli* (B1–B3) at the MIC and 4× the MIC. The data obtained are from the experiments performed in triplicate.

As compared to conventional antibiotics, AMPs are known to exert bacterial killing at a rapid pace. To examine whether this ability is also inherent to the lead peptide **5a**, the viability of exponentially growing antibiotic-resistant strains of *S. aureus* and *E. coli* was determined by conducting a 4 h time-kill assay (Figure 3).

Peptide **5a** exerted a time-dependent killing action against MRSA and eliminated almost all bacterial cells in 3.5 h at the MIC and in 2 h at 4× the MIC. On the other hand, peptide **5a** at the MIC displayed comparatively less rapid action against *E. coli* as complete eradication of the bacteria was not achieved even after a 4 h treatment. However, at 4× the MIC, a rapid killing action, comparable to MRSA, was observed with complete eradication of *E. coli* cells after a 2 h treatment. Noticeably, against MRSA and *E. coli*, the kinetics of bactericidal action of peptide **5a** was closely matched with the peptide-based antibiotics daptomycin and polymyxin B. However, it was found to be superior to the conventional antibiotic ciprofloxacin. Overall, bactericidal kinetic study results revealed the rapid killing action of peptide **5a** against both MRSA and *E. coli*. Many previous reports on AMPs with rapid bactericidal action demonstrated membrane disruption as their preferred mode of action. Thus, the rapid bacterial killing effect of peptide **5a** suggests that AMP antibacterial action might be mediated through membrane perturbation.

### 2.7 Membrane Disruption Action

#### 2.7.1 Calcein Dye Leakage Assay

**Figure 4:**
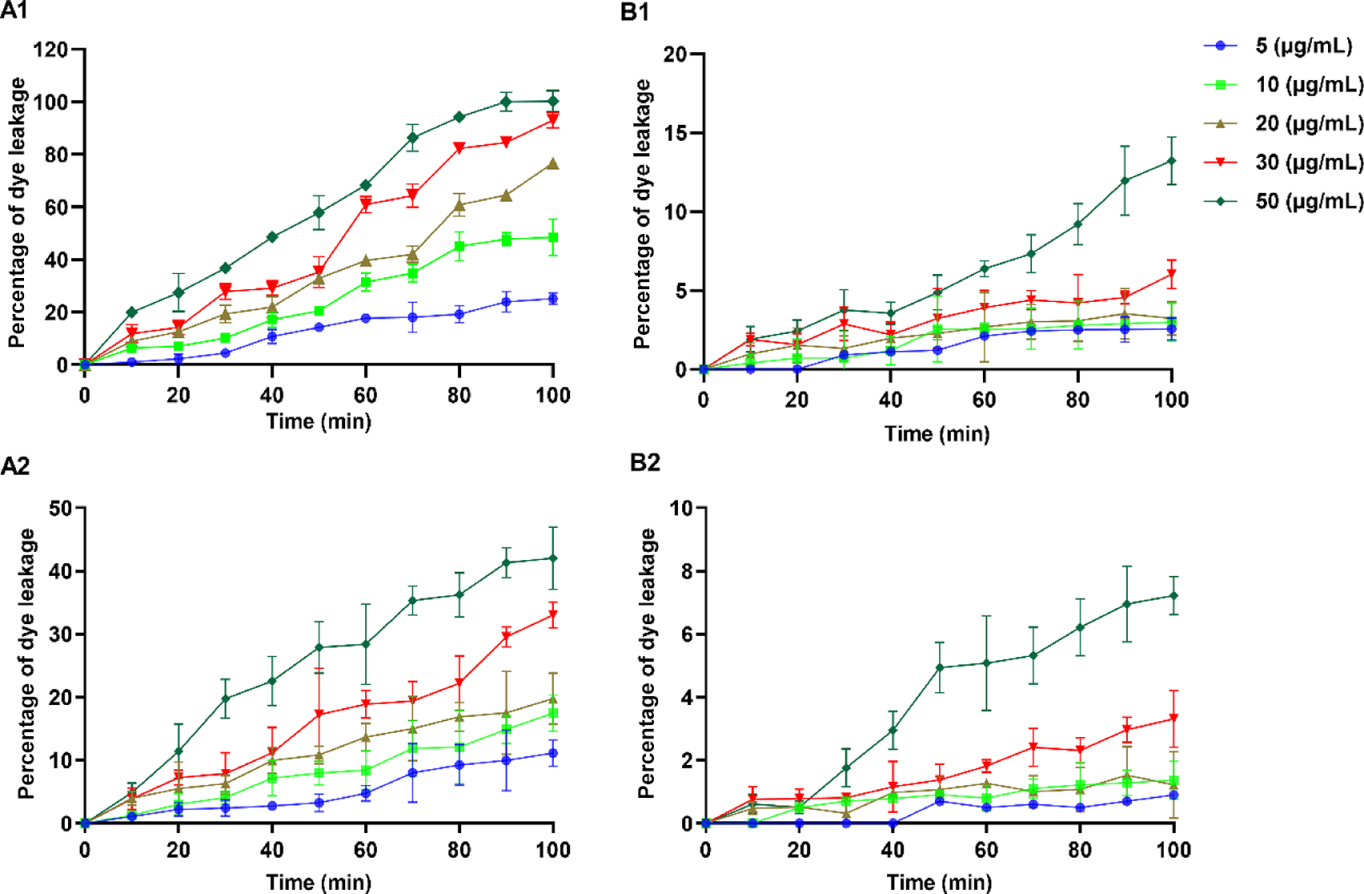
Concentration-dependent leakage of the calcein dye from bacterial membrane-mimicking (A1,A2) and mammalian membrane-mimicking (B1,B2) liposomes. **5a** (A1,B1) and daptomycin (A2,B2). The data obtained are from the experiments performed in triplicate.

Membrane disruption is considered a preferred mode of action for most naturally occurring and synthetic AMPs. To study the membranolytic action of the lead cyclic peptide **5a**, we performed a calcein dye leakage experiment by employing negatively charged and zwitterionic calcein-encapsulated lipid vesicles, which mimic the outer surface of the bacterial and mammalian cell membrane, respectively. Peptide **5a** induced calcein dye leakage that was measured at various concentration levels ranging from 5 to 50 μg/mL at different time intervals (Figure 4). The results demonstrate a concentration-dependent leakage of the dye upon treatment of calcein-encapsulated bacterial membrane-mimicking liposomes with peptide **5a**. At 5 μg/mL, peptide **5a** induced around 25% of dye release after 100 min of incubation. With the increase in the concentration of peptide **5a**, a sharp increase in the amount of dye leakage was observed. At the highest experimental concentration (50 μg/mL), peptide **5a** exerted 100% dye leakage after 90 min of incubation.

Interestingly, peptide **5a** induced a nonsignificant amount of dye leakage when incubated with liposomes mimicking the mammalian membrane, as indicated by around 13% dye leakage after 100 min. Compared to peptide **5a**, daptomycin at 50 μg/mL resulted in mild dye leakage when incubated with liposomes mimicking the bacterial membrane as suggested by only 42% of dye release observed after 100 min of incubation. These results agree with the previous reports on the lipid membrane interaction of daptomycin. A negligible amount of dye leakage (around 7%) was observed for daptomycin (50 μg/mL) when incubated with mammalian membrane-mimicking liposomes for 100 min. The calcein dye leakage experiments indicated that, like most native AMPs, the antibacterial activity of peptide **5a** depends on its ability to destabilize the target bacterial membrane.

#### 2.7.2 Field-Emission Scanning Electron Microscopy (FE-SEM)

**Figure 5:**
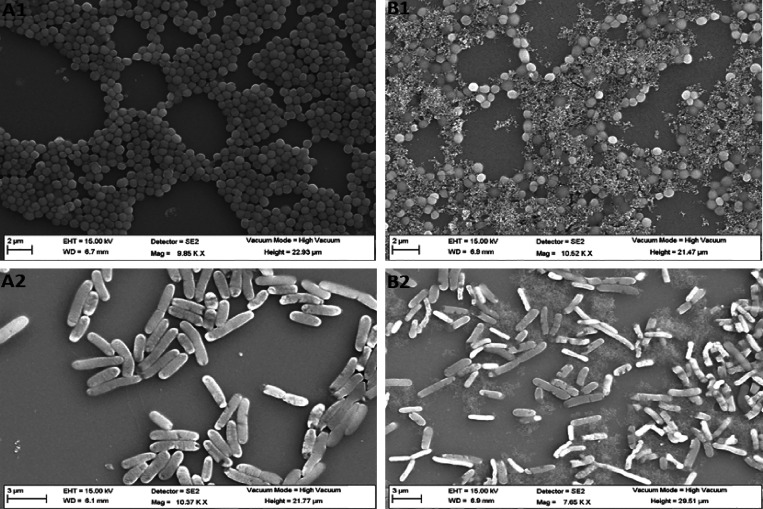
FE-SEM images of MRSA (A1,B1) and *E. coli* (A2,B2). Mid-logarithmic-phase bacterial cells were incubated with **5a** (A2,B2) at a final concentration of 4× the MIC for 1 h. The control (A1,A2) was done without peptides.

To further confirm the membranolytic action of the lead peptide **5a**, we examined the treated MRSA and *E. coli* cells using FE-SEM. The SEM micrographs explicitly indicate the membrane-damaging properties of peptide **5a** against both MRSA and *E. coli*. The untreated cells exhibit a regular size and shape with bright and smooth surfaces (Figure 5A1,B1). Treatment of bacterial cells with peptide **5a** at 4× the MIC exerted intense cell-specific morphological changes. While the loss of membrane fluidity is quite evident for both MRSA and *E. coli*, strong membrane atrophy was observed for *E. coli*. These data point toward the different modes of membrane disruption of peptide **5a** against MRSA and *E. coli*.

### 2.8 Resistance Development Study

**Figure 6:**
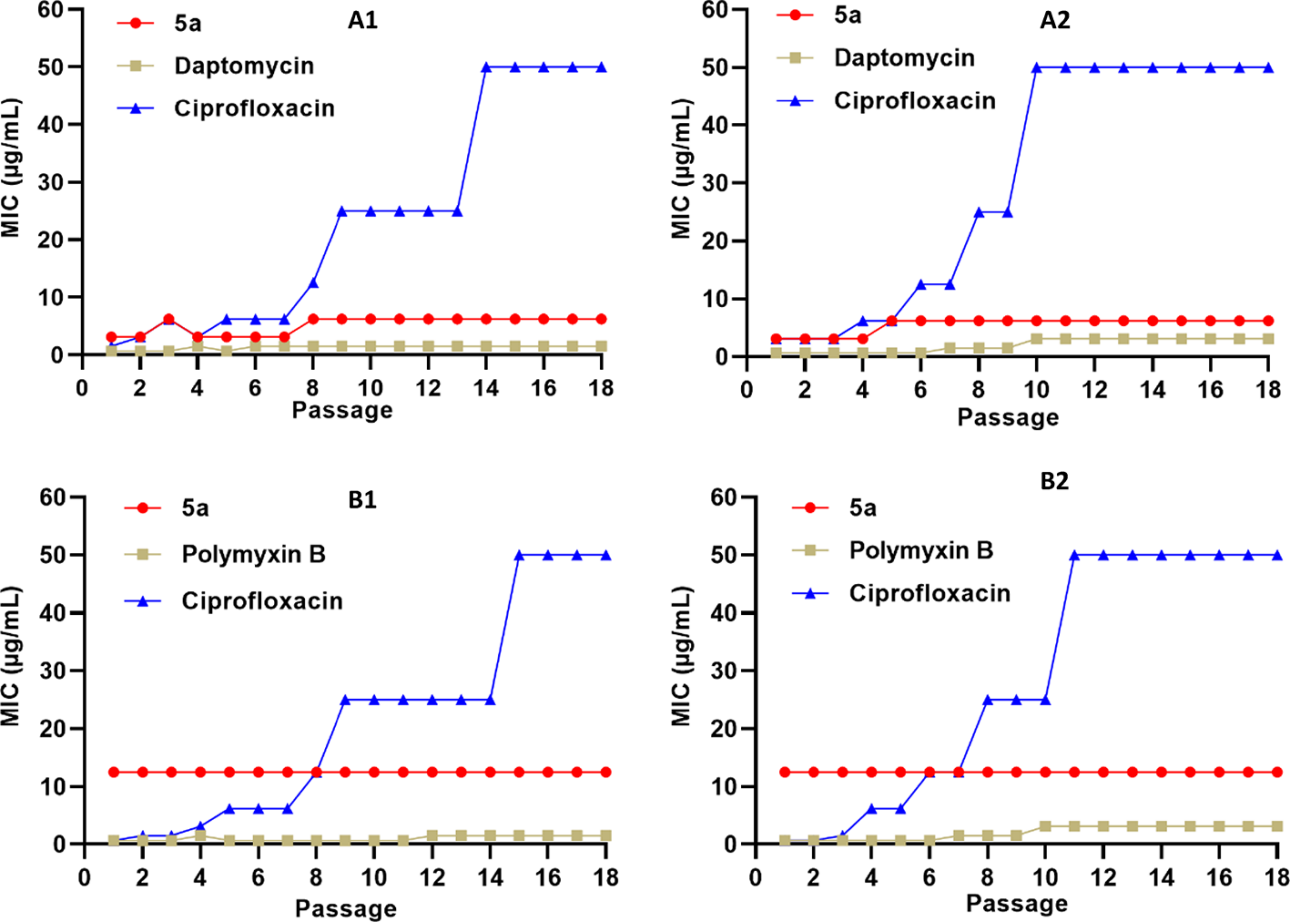
Resistance induction after 18 repeated times of exposure of the lead cyclic peptide (**5a**) and standard antibiotics (daptomycin, polymyxin B, and ciprofloxacin) against (A1) *S. aureus* (ATCC 29213), (A2) MRSA (ATCC BAA-1556), (B1) *E. coli* (ATCC 25922), and (B2) *E. coli* (ATCC BAA-2452). The data represent the experiments performed in triplicate.

Due to the extraordinary ability of bacteria to develop resistance, the effectiveness of conventional antibiotics in treating infections caused by resistant pathogens is continuously diminishing. Therefore, resistance development is considered one of the potential menaces associated with the clinical use of conventional antibiotics. Considering the membrane disruption effect of peptide **5a**, as indicated by the results of the calcein dye leakage experiment and SEM images, we anticipated that, similar to other peptide-based antibacterial agents, it will be difficult for bacteria to develop resistance. To examine the ability of bacteria to develop resistance against peptide **5a**, we performed resistance acquisition studies using resistant and susceptible strains of *S. aureus* and *E. coli.* To make a comparative analysis, *S. aureus* strains were treated with daptomycin and ciprofloxacin, and *E. coli* strains were treated with polymyxin B and ciprofloxacin. After each successive exposure of bacteria with different test specimens, a new MIC was determined as an indicator of the resistance development (Figure 6). Interestingly, similar to the peptide-based antibiotics (daptomycin and polymyxin B), negligible changes in the MICs were observed for peptide **5a** against all the tested bacterial strains. However, contrary to that, a sharp increase in the MICs of ciprofloxacin was observed against all the tested bacterial strains. Taken together, these results indicate that peptide **5a** holds a remarkable therapeutic potential to treat infections caused by resistant strains and therefore demand further investigation.

### 2.9 Plasma Stability

**Figure 7:**
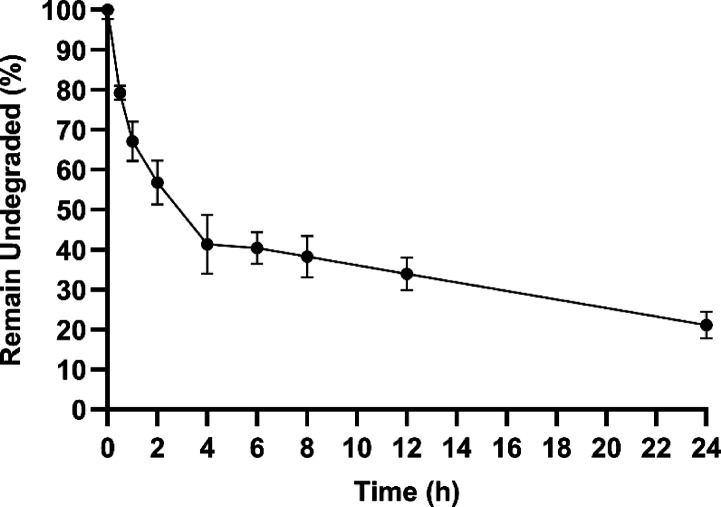
*In vitro* enzymatic stability assay of the lead cyclic peptides **5a** in human plasma. The data represent the percentage of undegraded peptides measured using Q-TOF LC/MS as the area under the curve in the extracted ion chromatogram. The data obtained are from three independent experiments.

Most of the naturally occurring AMPs are structurally linear and relatively large molecules. They, therefore, have a number of scissile amide bonds, which makes them an easy target for many peptidases. Consequently, the inherent enzymatic instability renders these potent antimicrobial molecules inactive in the intended biological environment. Therefore, this issue needs to be resolved for the success of peptide-based therapeutics in clinical settings. In this respect, we examined the stability of peptide **5a** in human blood plasma for up to 24 h (Figure 7). After 30 min of incubation, around 79% of the intact peptide **5a** was observed. Moreover, after 2 h of incubation, around 56% of the peptide remains undegraded. Around 25% of the intact peptide **5a** was observed after 24 h of incubation. The stability study results indicate that peptide **5a** has a half-life (*t*_1/2_) of approximately 3 h. It appears that head-to-tail cyclization of the peptides imparts extra plasma stability.

## 3 Conclusions

A library of cyclic amphiphilic peptides was designed and synthesized via solid-phase peptide synthesis. The synthesized library consisted of R and W residues as a recurring sequence and an ordered manner [R_*n*_W_*n*_] (*n* = 2–7) by systematically changing the number of R and W residues to study the impact of the hydrophilic/hydrophobic ratio, charge, and ring size on antibacterial activity. The minimum inhibitory concentration (MIC) values for all synthesized peptides were measured against multidrug-resistant and nonresistant Gram-positive and Gram-negative bacterial strains. The antibacterial activity was determined against various bacterial strains, including ESKAPE pathogens, and the minimum bactericidal concentration (MBC) was investigated for the most promising peptides. Among all the synthesized peptides, **5a** showed significant antibacterial activity alone and in combination with commercial antibiotics. Peptide **5a** showed remarkable improvement in a physical mixture combination (1:1 w/w) for antibiotic efficacy against all tested strains. Furthermore, **5a** displayed synergistic interactions with significant improvement when combined with tetracycline, tobramycin, clindamycin, kanamycin, levofloxacin, polymyxin B, metronidazole, and vancomycin. Hemolysis and cytotoxicity data demonstrated the selectivity of **5a** against bacteria versus mammalian cells. The membranolytic effect of **5a** was shown using a calcein dye leakage experiment and SEM. We concluded that cyclic peptide **5a** showed promising antibacterial potential to be further developed as an AMP with a reduced chance of resistance development.

## 4 Experimental Section

### 4.1 Materials

Tryptophan-loaded H-Trp(Boc)-2-chlorotrityl resin and Fmoc-amino acid building blocks, Fmoc-Arg(Pbf)-OH and Fmoc-Trp(Boc)-OH, were obtained from AAPPTec (Louisville, KY, USA). Chemical reagents and solvents were purchased from MilliporeSigma (Milwaukee, WI, USA) and used without further purification. Final products were purified using a reversed-phase high-performance liquid chromatography (RP-HPLC) system (LC-20AP) from Shimadzu (Canby, OR, USA), with a gradient system of acetonitrile and water with 0.1% TFA (v/v), and a reversed-phase preparative column (Waters XBridge, BEH130, 10 μm, 110 Å, 21.2 × 250 mm), with a flow rate of 8 mL/min and detection at 214 nm. The purity analysis of the peptides was conducted on an RP-HPLC system (Shimadzu; LC-20ADXR) by using an analytical Phenomenex (Luna) C18 column (4 μm, C18, 150 × 4.6 mm) with a flow rate of 1 mL/min and detection at 214 nm. The chemical structures of the final products were elucidated using a high-resolution MALDI-TOF instrument (model no. GT 0264 from Bruker, Inc., Fremont, CA, USA) with α-cyano-4-hydroxycinnamic acid as a matrix in the positive mode.

For bacterial strains, multidrug-resistant strains *Pseudomonas aeruginosa* (ATCC BAA-1744), *Klebsiella pneumoniae* (ATCC BAA-1705), *Escherichia coli* (ATCC BAA-2452), *Acinetobacter baumannii* (ATCC BAA-1605), and methicillin-resistant *Staphylococcus aureus* (*S. aureus* BAA-1556) and nonresistant strains *Staphylococcus aureus* (*S. aureus* 29213), *Enterococcus faecium* (ATCC 27270), *Enterococcus faecium* (ATCC 700221), *Enterococcus faecalis* (ATCC 29212), *Enterococcus faecalis* (ATCC 51575), *Staphylococcus pneumoniae* (ATCC 49619), *Staphylococcus pneumoniae* (ATCC 51938), *Bacillus subtilis* (ATCC-6633), *Bacillus cereus* (ATCC-13061), *Pseudomonas aeruginosa* (ATCC 27883), *Pseudomonas aeruginosa* (ATCC 10145), *Klebsiella pneumoniae* (ATCC 13883), and *Escherichia coli* (ATCC 25922) were obtained from American Type Culture Collection (ATCC; USA). The media for bacterial experiments were purchased from Hardy Diagnostics (Santa Maria, CA, USA) and are shown in Table S1 (Supporting Information). Clinical isolates are shown in Table S2 (Supporting Information). Ultrapure water was from a Milli-Q system (Temecula, CA, USA). An MTS assay kit (98%) was purchased from Promega (Madison, WI, USA). Single donor human plasma K2 EDTA was purchased from Innov-Research (Novi, MI, USA). All phospholipids and cholesterol were purchased from Avanti Polar Lipids (Alabaster, USA). A calcein dye was obtained from Sigma. All the mammalian cell culture supplies were purchased from Corning (Christiansburg, VA, USA) and Fisher Scientific (Waltham, MA, USA). All the mammalian cell and bacterial experiments were carried out under a laminar flow hood from Labconco (Kansas City, MO, USA). Cell culture was carried out at 37 °C with 5% CO_2_ in a Forma incubator using a T-75 flask. All cells were maintained in a 5% CO_2_ incubator (37 °C). The human lung fibroblast cells (MRC-5, ATCC CCL-171), human embryonic kidney cells (HEK293, ATCC CRL 1573), human hepatoma HepaRG cells (Gibco, HPRGC10), and the human skin fibroblast cell line (HeKa, ATCC PCS-200-011) were purchased from ATCC (USA). All cells were maintained in a 5% CO_2_ incubator (37 °C). Human serum was purchased from Sigma-Aldrich. All bacterial strains employed in this study were procured from VWR, USA, and propagated as per the recommendation of ATCC.

### 4.2 Peptide Synthesis

The preloaded amino acid on resin, H-Trp(Boc)-2-chlorotrityl resin, and Fmoc-amino acid building blocks were used for synthesis on a scale of 0.3 mmol. *O*-(Benzotriazole-1-yl)-*N*,*N*,*N′*,*N*′-tetramethyluroniumhexafluorophosphate (HBTU) was used as a coupling agent, and *N*,*N*-diisopropylethylamine (DIPEA) was used as an activating reagent. Fmoc deprotection was achieved in the presence of piperidine in DMF (20% v/v). The side chain-protected peptides were assembled on the tryptophan-preloaded H-Trp(Boc)-2-chlorotrityl resin as described above on a scale of 0.3 mmol. After assembling the peptides on resin, the side chain-protected peptides were subjected to a TFE/acetic acid/DCM [2:1:7 (v/v/v)] cocktail for detaching the protected peptides from the resin and were then subjected to cyclization using 1-hydroxy-7-azabenzotriazole (HOAt) (136 mg, 1 mmol) and 1,3-diisopropylcarbodiimide (DIC) (207 μL, 1.33 mmol) in the mixture of anhydrous DMF/DCM (5:1 v/v, 300 mL). The cyclization reaction occurred overnight under an inert condition using nitrogen. All the protecting groups were removed by stirring at room temperature in the presence of the cleavage cocktail of the freshly prepared reagent R containing TFA/thioanisole/1,2-ethanedithiol (EDT)/anisole (90:5:3:2, v/v/v/v) for 3 h. The crude product was precipitated by the addition of cold diethyl ether. Peptides were dissolved in an acetonitrile/water mixture to a final concentration of 20 mg/mL. Following filtration through a 0.45 μm Millipore filter, the peptides were purified using RP-HPLC with a gradient of 0–90% acetonitrile (0.1% TFA) and water (0.1% TFA) over 60 min with a C-18 column. The purified peptides were lyophilized to yield a white powder (around 100 mg). The purity of synthesized cyclic peptides was determined using reversed-phase analytical HPLC (Shimadzu; LC-20ADXR) and found to be ≥95%.

**1** [R_4_W_4_]: HR-MS (MALDI-TOF) (*m*/*z*) [C_68_H_88_N_24_O_8_]: calcd, 1368.7422; found, 1369.7590 [M + H]^+^; **2a** [R_2_W_3_]: HR-MS (MALDI-TOF) (*m*/*z*) [C_45_H_54_N_14_O_5_]: calcd, 870.4402; found, 871.1137 [M + H]^+^; **2b** [R_2_W_4_]: HR-MS (MALDI-TOF) (*m*/*z*) [C_56_H_64_N_16_O_6_]: calcd, 1056.5195; found, 1057.3387 [M + H]^+^; **3a** [R_3_W_3_]: HR-MS (MALDI-TOF) (*m*/*z*) [C_51_H_66_N_18_O_6_]: calcd, 1026.5518; found, 1026.5966 [M]^+^; **3b** [R_3_W_4_]: HR-MS (MALDI-TOF) (*m*/*z*) [C_62_H_76_N_20_O_7_]: calcd, 1212.6206; found, 1213.1192 [M + H]^+^; **3c** [R_3_W_5_]: HR-MS (MALDI-TOF) (*m*/*z*) [C_73_H_86_N_22_O_8_]: calcd, 1398.6444; found, 1399.9105 [M + H]^+^; **3d** [R_3_W_6_]: HR-MS (MALDI-TOF) (*m*/*z*) [C_84_H_96_N_24_O_9_] calcd, 1584.7792; found, 1584.7702 [M]^+^; **3e** [R_3_W_7_]: HR-MS (MALDI-TOF) (*m*/*z*) [C_95_H_106_N_26_O_10_]: calcd, 1770.8585; found, 1771.6164 [M + H]^+^; **4a** [dR_4_W_4_]: HR-MS (MALDI-TOF) (*m*/*z*) C_68_H_88_N_24_O_8_: calcd, 1368.7217; found, 1369.0454 [M + H]^+^; **4b** [R_4_W_5_]: HR-MS (MALDI-TOF) (*m*/*z*) [C_79_H_98_N_26_O_9_]: calcd, 1554.8320; found, 1555.1560 [M + H]^+^, 1577.1408 [M + Na]^+^; **4c** [R_4_W_6_]: HR-MS (MALDI-TOF) (*m*/*z*) [C_90_H_108_N_28_O_10_]: calcd, 1740.8908; found, 1741.8152 [M + H]^+^; **4d** [R_4_W_7_]: HR-MS (MALDI-TOF) (*m*/*z*) [C_101_H_118_N_30_O_11_]: calcd, 1926.9701; found, 1927.9072 [M + H]^+^; **5a** [R_5_W_4_]: HR-MS (MALDI-TOF) (*m*/*z*) [C_74_H_100_N_28_O_9_]: calcd, 1524.8433; found, 1525.6209 [M + H]^+^; **5b** [R_5_W_5_]: HR-MS (MALDI-TOF) (*m*/*z*) [C_85_H_110_N_30_O_10_]: calcd, 1710.9126; found, 1710.4793 [M]^+^; **5c** [R_5_W_6_]: HR-MS (MALDI-TOF) (*m*/*z*) [C_96_H_122_N_32_O_12_]: calcd, 1897.1260; found, 1898.4210 [M + H]^+^, 1920.4116 [M + Na]^+^; **5d** [R_5_W_7_]: HR-MS (MALDI-TOF) (*m*/*z*) [C_107_H_130_N_34_O_12_]: calcd, 2083.0607; found, 2084.2055 [M + H]^+^; **6a** [R_6_W_4_]: HR-MS (MALDI-TOF) (*m*/*z*) [C_80_H_112_N_32_O_10_]: calcd, 1680.9344; found, 1680.0997 [M]^+^; **6b** [R_6_W_5_]: HR-MS (MALDI-TOF) (*m*/*z*) [C_91_H_122_N_34_O_10_]: calcd, 1867.0237; found, 1866.5769 [M]^+^; **6c** [R_6_W_6_]: HR-MS (MALDI-TOF) (*m*/*z*) [C_102_H_132_N_36_O_12_]: calcd, 2053.0825; found, 2054.1105 [M + H]^+^; **6d** [R_6_W_7_]: HR-MS (MALDI-TOF) (*m*/*z*) [C_113_H_142_N_38_O_13_]: calcd, 2239.1723; found, 2240.3233 [M + H]^+^; **7a** [R_7_W_4_]: HR-MS (MALDI-TOF) (*m*/*z*) [C_86_H_124_N_36_O_11_]: calcd, 1837.0455; found, 1839.3871 [M + 2H]^+^; **7b** [R_7_W_5_]: HR-MS (MALDI-TOF) (*m*/*z*) [C_97_H_134_N_38_O_12_]: calcd, 2023.1043; found, 2023.9510 [M + H]^+^; **7c** [R_7_W_6_]: HR-MS (MALDI-TOF) (*m*/*z*) [C_108_H_144_N_40_O_13_]: calcd, 2209.1837; found, 2210.1507 [M + H]^+^; **7d** [R_7_W_7_]: HR-MS (MALDI-TOF) (*m*/*z*) [C_119_H_154_N_42_O_14_]: calcd, 2395.2630; found, 2396.2736 [M + H]^+^.

### 4.3 Antimicrobial Assays

#### 4.3.1 Minimum Inhibitory Concentration (MIC) Determination

The antibacterial activities of all synthesized compounds were evaluated against Gram-positive and Gram-negative bacteria. The selected bacterial strains were cultured according to the guidelines of the Clinical Laboratory Standards Institute (CLSI) guidelines. The minimum inhibitory concentration (MIC) was determined by microbroth dilution. The minimal concentration was determined to be at a concentration in wells in which no visible bacterial growth was present. An aliquot of an overnight culture of bacteria was grown in suitable media (Table S1, Supporting Information) diluted in 1 mL of normal saline to achieve 0.5 McFarland turbidity (1.5 × 10^8^ bacterial cell CFU/mL). A volume of 60 μL of the 0.5 McFarland solution was added to 8940 μL of MH media (this was a 1/150 dilution). A concentration of 512 μg/mL of the tested peptides was prepared from a stock solution of the samples for testing in Müller–Hinton broth MH media. An amount of 100 μL of MH media was pipetted into the sterile 96-well plate except for the first well. An amount of 200 μL of 512 μg/mL compound samples was added by a pipette into the first well and serially diluted with the MH media along sterile 96 wells using a multitip pipette except the last well. An amount of 100 μL of aliquot of bacterial solution was added to each well, and the plate was incubated at 37 °C for 24 h. All experiments were conducted in triplicate.

#### 4.3.2 Minimum Bactericidal Concentration (MBC) Determination

MBC is the lowest concentration of an antibacterial agent required to kill a bacterium over a fixed period, such as 24 h, under a specific set of conditions. We determined the MBC of the promising peptides (**5a**, **5b**, **6a**, and **6b**) from the broth dilution of MIC tests by subculturing them to agar plates and applying 24 h of incubation at 37 °C. The MBC was identified by determining the lowest concentration of the antibacterial agent that reduced the viability (concentration of peptides necessary to achieve a bactericidal effect) of the initial bacterial inoculum by ≥99.9%.

#### 4.3.3 The Synergistic Checkerboard Assay

A checkerboard assay was performed in a sterile 96-well plate using a multichannel pipette (Tables S6 and S7, Supporting Information). An aliquot of an overnight culture of bacteria was grown in Luria broth (LB) diluted in 1 mL of normal saline to achieve 0.5 McFarland turbidity (1.5 × 10^8^ bacterial cell CFU/mL). A volume of 60 μL of the 0.5 McFarland solution was added to 8940 μL of MH media (this was a 1/150 dilution). The concentrations of the peptide started from its respective MICs and were then subjected to two-fold serial dilutions (a total of 7 points). The antibiotics’ concentration started from 128 μg/mL (11 points). Antibiotics were tested as 11 points, two-fold serial dilutions across the assay plate (from 1 to 11 columns) in combination with a seven (A–G)-point and two-fold serial dilution of the peptides down the assay plate. To determine the MIC value for each test compound, two-fold serial dilution in row H (from 1 to 11) for antibiotics alone was performed. In column 12 (A–G) down the assay plate, a two-fold serial dilution of the peptide alone was performed. Assay plates were inoculated with 100 μL of bacterial suspensions and incubated at 37 °C for 24 h.

The following equation was applied to calculate the FICI: (A)/MIC_A_ + (B)/MIC_B_ = FIC_A_ + FIC_B_ = FICI, where (A) and (B) are the MIC of (antibiotic + [peptide]) in combination (in a single well) and MIC_A_ and MIC_B_ are the MIC of each compound individually. Based on the calculated FICI value, the interaction between combinations was categorized into four prospects synergy, partial synergy, additive or indifference, and antagonism. FICI was interpreted as follows: FICI ≤ 0.5, synergy; 0.5 < FICI ≤ 1, partial synergy; 1 ≤ FICI < 4, additive effect or indifference; 4 ≤ FICI antagonism. All experiments were performed in triplicate.

#### 4.3.4 Time-Kill Kinetics Assay

The time course of bacterial killing was studied by exposure of overnight grown cultures of MRSA (ATCC BAA-1556) and *E. coli* (ATCC BAA-2452) to the lead cyclic peptide **5a** at the MIC and 4× the MIC in Müller–Hinton media with overnight grown bacterial culture (1.5 × 10^8^ CFU/mL) and incubation at 37 °C. Aliquots were sampled at 0, 0.5, 1, 2, 4, 8, 12, 18, and 24 h time points, then diluted up to 10^8^, and plated on the Müller–Hinton agar plate. After 24 h of incubation at 37 °C, the CFU count was performed using a standard formula. Untreated bacterial culture was used as a control. Data were obtained from two independent experiments performed in triplicate.

#### 4.3.5 Serial Passage Resistance Development Studies

We determined the potential of susceptible and antibiotic-resistant strains of *S. aureus* and *E. coli* to develop resistance against the lead peptide **5a**. For comparative purposes, we also included different standard antibiotics such as daptomycin, polymyxin B, and ciprofloxacin in the study. The initial MICs of peptide **5a** and standard antibiotics were determined using the protocol mentioned in Section 4.3.1 of the manuscript. The assays were performed in 96-well microtiter plates using 2-fold serial dilutions of all the test specimens. From an inoculated microtiter plate, an aliquot of the well with the highest concentration permitting growth was taken and diluted in fresh media to achieve the optimum bacterial load identified by comparing the turbidity with the 0.5 McFarland standard. The bacterial suspension was then further diluted and used for successive passage with a resulting final concentration of 5 × 10^5^ CFU/mL. MICs were recorded, and the next inoculum was prepared from the well containing the highest concentration of the drug that allowed growth. Eighteen repeated passages were performed in an identical fashion to that described above.

### 4.4 Hemolysis Assay

The hemolytic activity of all the cyclic peptides was determined using human red blood cells (hRBC) purchased from BioIVT, USA. The hRBC were centrifuged for 15 min to remove the buffy coat and washed three times with phosphate-buffered saline (PBS, 100 mM NaCl, 80 mM Na_2_HPO_4_, and 20 mM NaH_2_PO_4_, pH 7.4). Peptide-induced hemolysis was tested in triplicate by mixing 75 μL of the peptide solution in PBS (two-fold serial dilution) with 75 μL of a 4% (v/v) hRBC suspension in phosphate-buffered saline. The plates were incubated for 2 h at 37 °C without agitation and centrifuged at 1000*g* for 10 min. Aliquots (100 μL) of the supernatant were transferred to 96-well plates, where hemoglobin release was measured spectrophotometrically at 567 nm. Percent hemolysis was calculated by the following formula:
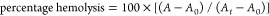
where *A* represents the absorbance of the peptide sample at 567 nm and *A*_0_ and *A_t_* represent zero percent and 100% hemolysis determined in phosphate-buffered saline and 1% Triton X-100, respectively.

### 4.5 Cell Viability Assay

The *in vitro* cytotoxicity of the lead cyclic peptide **5a** was evaluated using human lung fibroblast cells, human embryonic kidney cells, human hepatoma HepaRG cells, and the human skin fibroblast cell line. Cells were seeded at 10,000 per well in 0.1 mL of media in 96-well plates 24 h prior to the experiment. Lung and kidney cells were seeded in DMEM medium containing FBS (10%). Liver cells were seeded in William’s E medium with a GlutaMAX supplement. The peptides were added to each well in triplicates at a variable concentration of 12.5–250 μg/mL and incubated for 24 h at 37 °C in a humidified atmosphere of 5% CO_2_. After the incubation period, MTS solution (20 μL) was added to each well. Then, the cells were incubated for 2 h at 37 °C, and cell viability was determined by measuring the absorbance at 490 nm using a SpectraMax M2 microplate spectrophotometer. The percentage of cell survival was calculated as [(OD value of cells treated with the test mixture of compounds) – (OD value of culture medium)]/[(OD value of control cells) – (OD value of culture medium)] × 100%.

### 4.6 Calcein Dye Leakage Assay

#### 4.6.1 Preparation of Calcein-Encapsulated Liposomes

Large unilamellar vesicles (LUVs) of 1,2-dioleoyl-*sn*-glycero-3-phosphocholine (DOPC) and 1,2-dioleoyl-*sn*-glycero-3-phosphoglycerol (DOPG) were prepared to mimic the bacterial membrane (DOPC:DOPG, 7:3, w/w) or the mammalian membrane (DOPC/Chol, 10:1, w/w) as described previously.^98^ Briefly, a defined amount of the lipid mixture DOPC:DOPG (7:3, w/w) was dissolved in 2 mL of the chloroform/methanol solvent mixture in a 100 mL round-bottom flask. The solvents were removed under a stream of nitrogen, and the lipid film obtained was lyophilized overnight to remove any trace of solvent. The thin lipid film was rehydrated with calcein-containing buffer comprising 70 mM calcein, 150 mM NaCl, and 0.1 mM EDTA and adjusted to pH 7.4 by the addition of a few drops of sodium hydroxide solution (1 M). The liposome suspension obtained after rehydration was frozen and thawed for five cycles and extruded 15 times through two stacked polycarbonate filters (100 nm pore size). The free calcein was removed by passing the liposome suspension through a Sephadex G-50 column and eluting it with a buffer containing 10 mM Tris–HCl (150 mM NaCl, 0.1 mM EDTA). After passing the liposome through the Sephadex G-50, the liposome diameter was measured by dynamic light scattering using a Zetasizer Nano ZS (Malvern Instruments, USA). The average diameter of LUVs was found to be in the range of 115–120 nm.

#### 4.6.2 Measurement of Dye Leakage

A calcein dye leakage assay was conducted by using calcein encapsulated in large unilamellar vesicles (LUVs). Lead peptide **5a**-induced leakage of calcein from the LUVs was monitored by measuring the fluorescence intensity at an excitation wavelength of 490 nm and an emission wavelength of 520 nm on a SpectraMax M5 multimode microplate reader. To achieve a final lipid concentration of 40 μM, the liposome suspension was diluted in phosphate-buffered saline. The assay was conducted in triplicate by mixing 50 μL of peptide solution at various concentrations (5, 10, 20, 30, and 50 μg/mL) with 50 μL of the liposome suspension. Calcein release from LUVs was assessed for 100 min, and the measurement was taken at an interval of 10 min. The fluorescence intensity corresponding to 100% calcein release was determined by adding a 10% solution (w/v) of Triton X-100. The apparent percentage of dye leakage was calculated using the following formula:
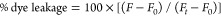
where *F* is the intensity measured at a given peptide concentration, *F*_0_ is the background intensity of the liposome sample, and *F_t_* is the intensity after lysis induced by Triton X-100.

### 4.7 SEM Analysis

Overnight grown bacterial cultures of MRSA (ATCC BAA-1556) and *E. coli* (ATCC BAA-2452) were suspended at 10^7^ CFU/mL in 10 mM PBS, pH 7.4. Aliquots of 250 μL of the bacterial suspension were mixed with 30 μL of peptide **5a** at a final concentration of 4× the MIC and were incubated at 37 °C for 1 h. Controls were run without the peptides. After 30 min, the cells were fixed with an equal volume of 4% glutaraldehyde in 0.2 M Na-cacodylate buffer, pH 7.4, for 3 h at 4 °C followed by dehydration with a graded series of ethanol and dried the sample in HMDS (hexamethyl disilazane). Coating was done with Au/Pd at approximately 20 nm thicknesses and observed under a scanning electron microscope (Zeiss Sigma 300).

### 4.8 Plasma Stability Study

An *in vitro* stability study of the lead cyclic peptide **5a** was performed by using LC–MS as described previously. For accurate quantification, we employed a mass spectrometer (Q-TOF LC/MS) with a high resolving power. To remove the background interference, the data were processed by using a narrower (±0.1 *m*/*z*) extracted ion chromatogram (EIC) window. The test peptide (100 μM) was incubated in human plasma at 37 °C in a water bath. Aliquots (10 μL) were taken at different time points (0, 0.5, 1, 2, 4, 6, 8, 12, and 24 h), diluted by the addition of 6 M guanidine hydrochloride (10 μL, pH 2), and incubated at room temperature for 30 min. Plasma proteins were precipitated by the addition of cold ethanol (200 μL) and incubated for 30 min at −20 °C. The samples were centrifuged at 9000*g* for 15 min, and the supernatant was transferred to a new tube and concentrated under a vacuum. The samples were dissolved in water and analyzed on a Delta-Pak (Waters) C18 column (100 Å, 5 μm, 3.9 mm × 150 mm) at a flow rate of 0.3 mL/min using a linear gradient of aqueous acetonitrile in the presence of formic acid (FA; 0.1%, v/v) as an ion pair reagent. Peptides were detected by recording the absorbance at 220 nm and quantified by their peak areas of EIC. The percentage digestion of the test peptides was calculated relative to the peak areas at 0 min.

## Supporting Information Available

Mass spectra (Figures S1–S24), analytical HPLC data of the synthesized compounds (Figures S25–S48), media used for growth of bacteria (Table S1), strains and clinical isolates tested in the study (Table S2), MIC values of **5a** and **6a** in the presence of salts and serum (Table S3), MIC values of the physical mixture of **5a** and **6a** with commercially available antibiotics (Tables S4 and S5), representative checkerboard assay (Tables S6 and S7), and plasma stability (Figure S49) (PDF)
Molecular formula strings (CSV)

## ABBREVIATIONS

AMP: antimicrobial peptide
CFU: colony-forming units
DIC: *N*,*N*-diisopropylcarbodiimide
DIPEA: *N*,*N*-diisopropylethylamine
DMF: *N*,*N*-dimethylformamide
HOAt: 1-hydroxy-7-azabenzotriazole
HOBt: 1-hydroxybenzotriazole
HPLC: high-performance liquid chromatography
MeCN: acetonitrile
MHB: Müller–Hinton broth
TFA: trifluoroacetic acid
TFE: 2,2,2-trifluoroethanol
