# Supplementary material for: Membrane-Active Cyclic Amphiphilic Peptides: Broad-Spectrum Antibacterial Activity Alone and in Combination with Antibiotics
Source: J Med Chem. 2022 Nov 28;65(23):15819–39. doi: 10.1021/acs.jmedchem.2c01469 (PMC9743092; doi:10.1021/acs.jmedchem.2c01469)
Supplement: Supplementary file 1 — jm2c01469_si_001.pdf [file jm2c01469_si_001.pdf]

## Supporting Information

# Membrane-Active Cyclic Amphiphilic Peptides: Broad-Spectrum Antibacterial Activity Alone and in Combination with Antibiotics

Eman H. M. Mohammed,[§,$,†,#] Sandeep Lohan,[§,†,#] Tarra Ghaffari,[§] Shilpi Gupta,[§] Rakesh K. Tiwari,[§,]* Keykavous Parang[§,]*

[§]Center for Targeted Drug Delivery, Department of Biomedical and Pharmaceutical Sciences, Chapman University School of Pharmacy, Harry and Diane Rinker Health Science Campus, Irvine, California 92618, United States

[$]Department of Chemistry, Faculty of Science, Menoufia University, Shebin El-Koam, 51132, Egypt

[†]AJK Biopharmaceutical, Irvine, California 92617, United States

[#]These authors contributed equally.

**Corresponding Authors**

Keykavous Parang − Center for Targeted Drug Delivery, Department of Biomedical and Pharmaceutical Sciences, Chapman University School of Pharmacy, Irvine, California92618, United States; orcid.org/0000-0001-8600-0893; Phone: +1-714-516-5489; Email: parang@chapman.edu; Fax: +1-714-516-5481.

Rakesh K. Tiwari − Center for Targeted Drug Delivery, Department of Biomedical and Pharmaceutical Sciences, Chapman University School of Pharmacy, Irvine, California 92618, United States; orcid.org/0000-0003-3701-4548; Phone: +1-714-516-5483; Email: tiwari@chapman.edu; Fax: +1-714-516-5481

[#]These authors contributed equally.

**Table of Contents** **Page**


| | |
|---|---|
| MALDI-TOF Mass Data of Synthesized Peptides (Figures S1-S24) | S3 |
| HPLC Analysis of Peptides (Figures S25-S44) | S26 |
| Additional HPLC Analysis of Lead Peptides (Figures S45-48) | S37 |
| **Tables S1.** Media Required for Growth of the Bacterial Strains | S40 |
| **Table S2.** Bacterial Strains and clinical isolates tested in the study | S42 |
| **Table S3.** MIC values of peptides **5a** and **6a** in the presence of salts and serum | S43 |
| **Table S4.** MIC values of physical mixture of **5a** with commercially available antibiotics (1:1 w/w) | S44 |
| **Table S5.** MIC values of physical mixture of **6a** with commercially available antibiotics (1:1 w/w) | S45 |
| **Table S6.** Checkerboard assay of [$R_5W_4$] + clindamycin using *K. pneumoniae* (ATCC BAA-1705) | S46 |
| **Table S7.** Results and data analysis of checkerboard Assay of [$R_5W_4$] + Clindamycin using *K. pneumoniae* (ATCC BAA-1705) | S46 |
| Plasma stability of **5a** conducted in triplicate study | S47 |

**MALDI-TOF Mass Data of Synthesized Peptides**

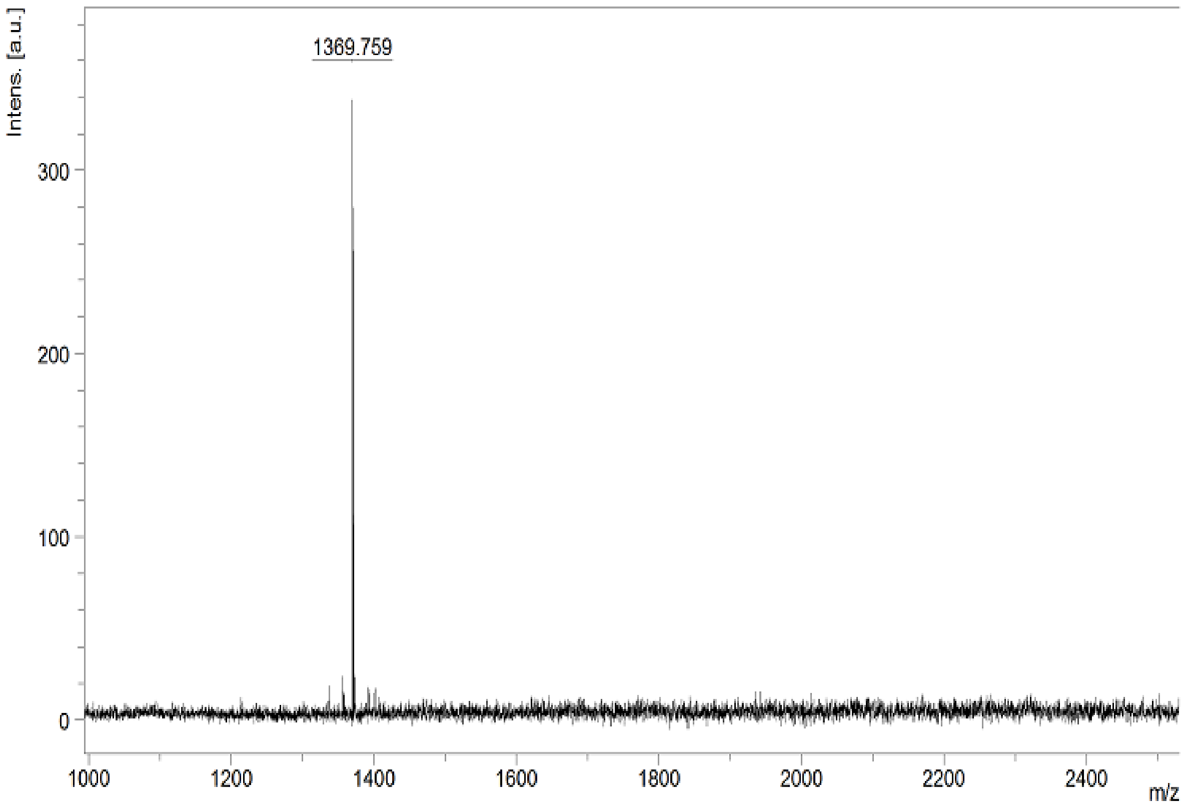


**Figure S1.** MALDI-TOF (m/z) for [$R_4W_4$] **(1)**.

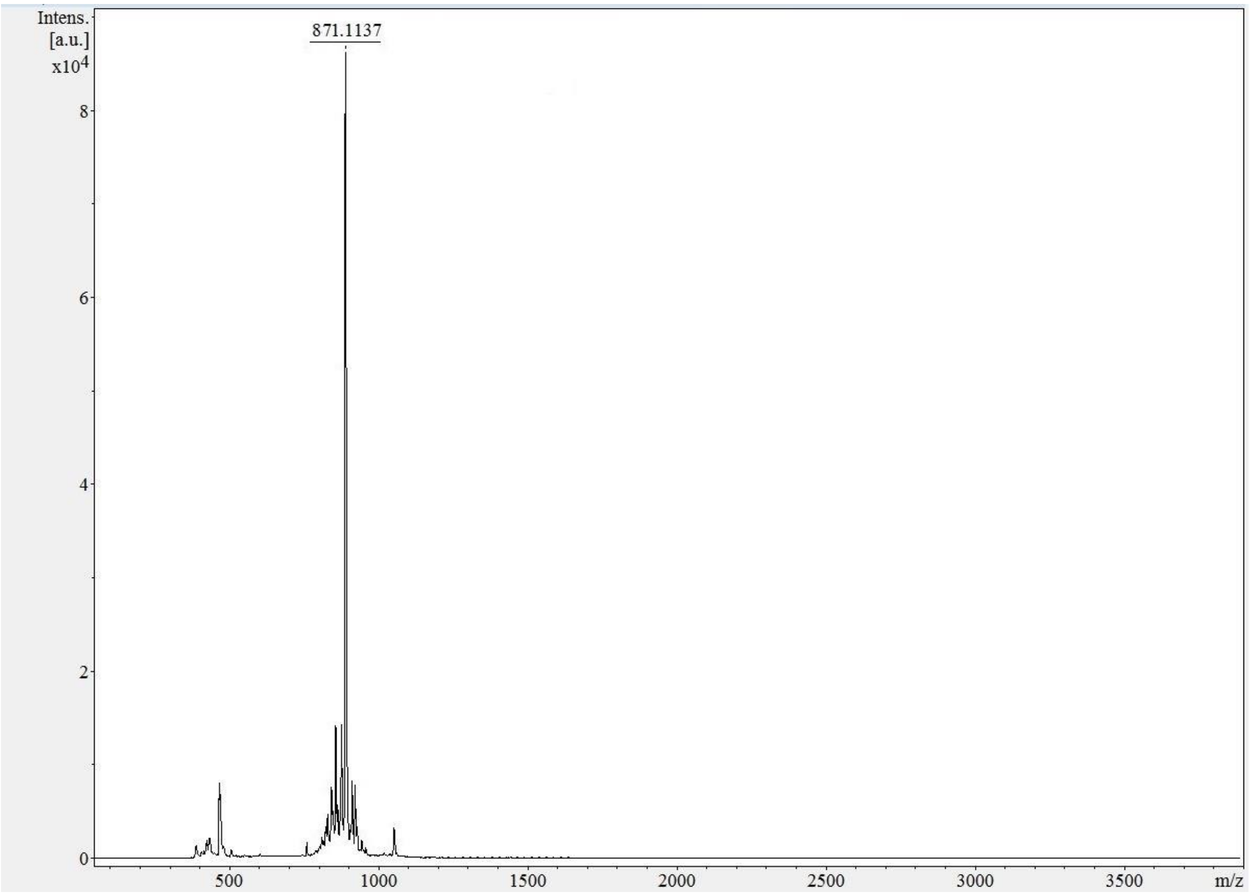


**Figure S2.** MALDI-TOF (m/z) for $[R_2W_3]$ **(2a)**.

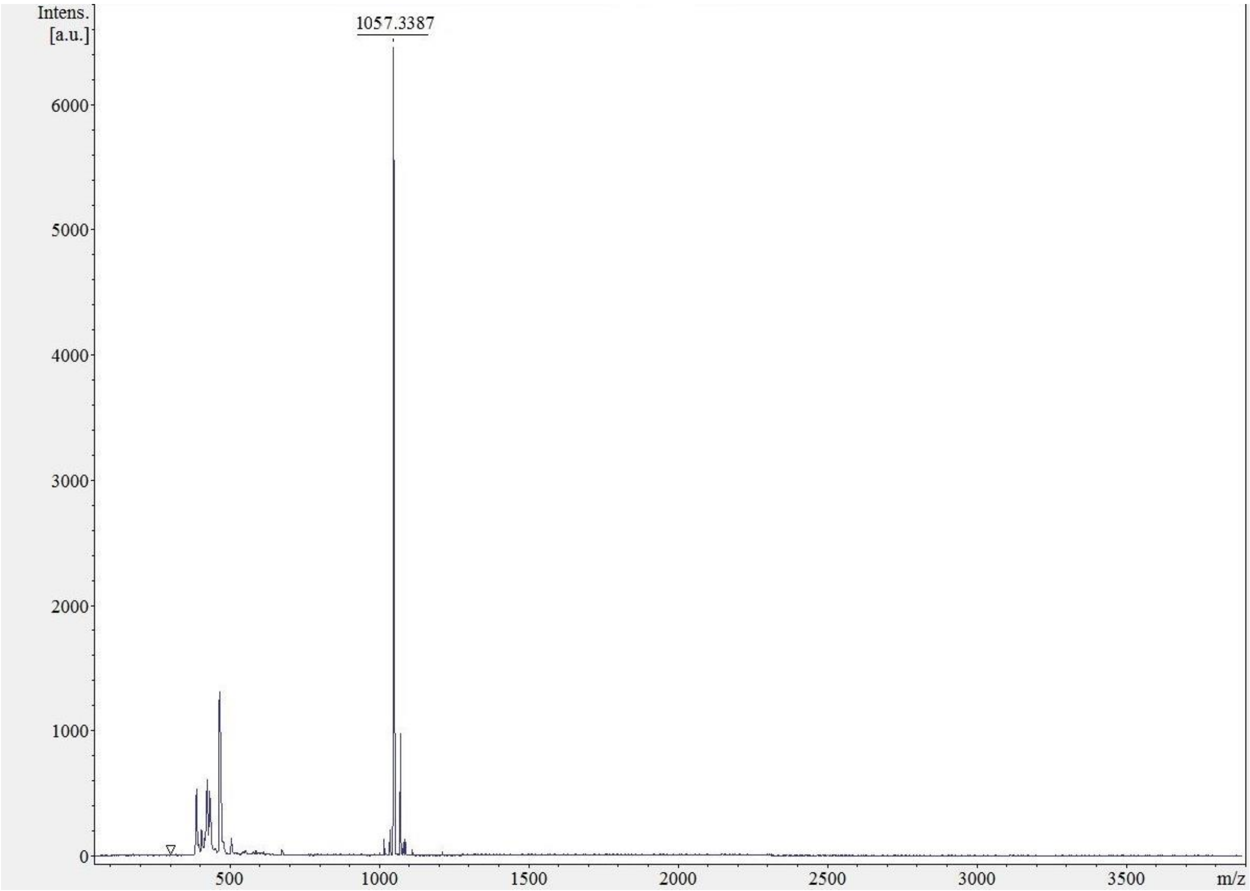


**Figure S3.** MALDI-TOF (m/z) for [$R_2W_4$] **(2b)**.

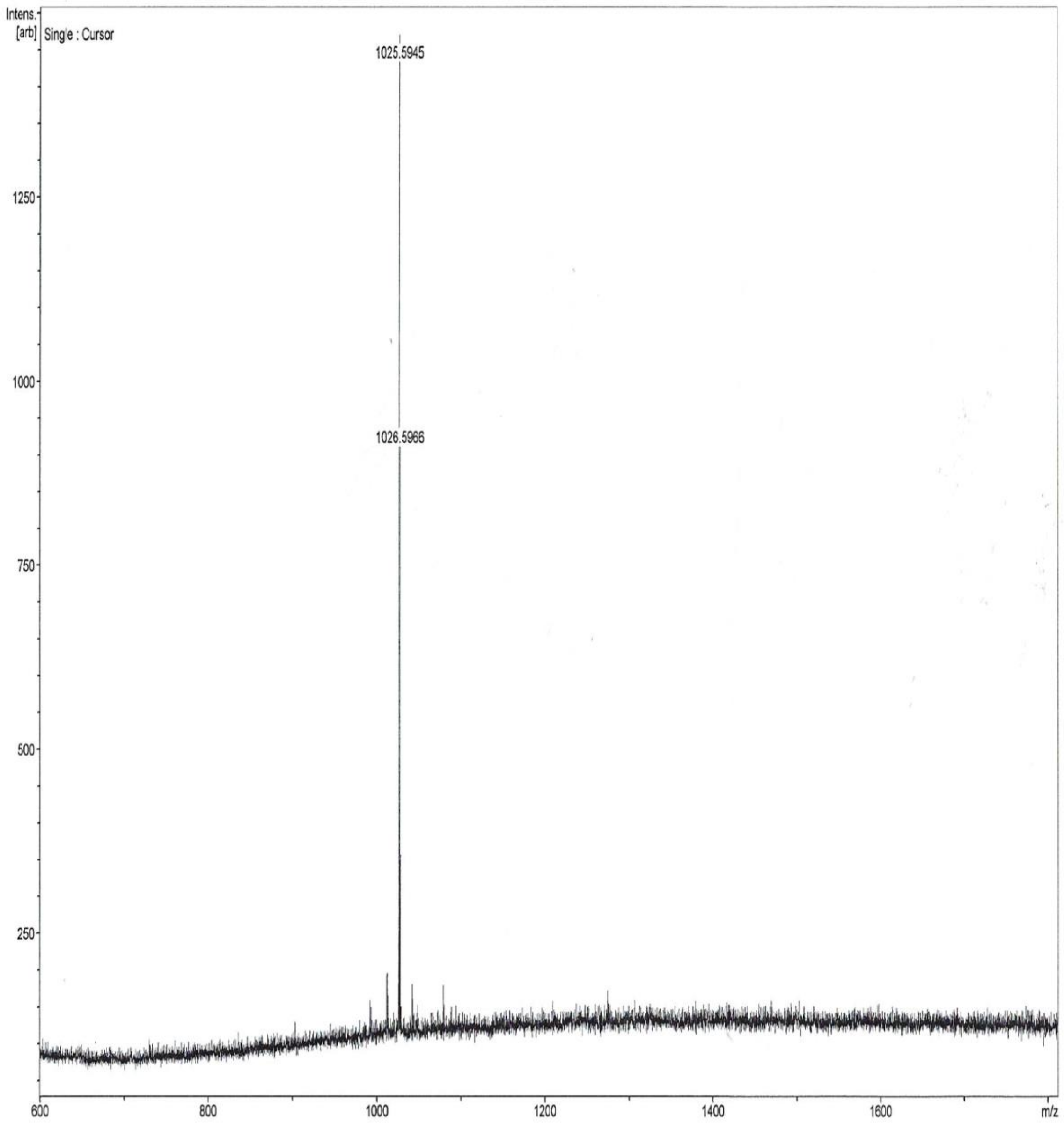


**Figure S4.** MALDI-TOF (m/z) for [$R_3W_3$] **(3a)**.

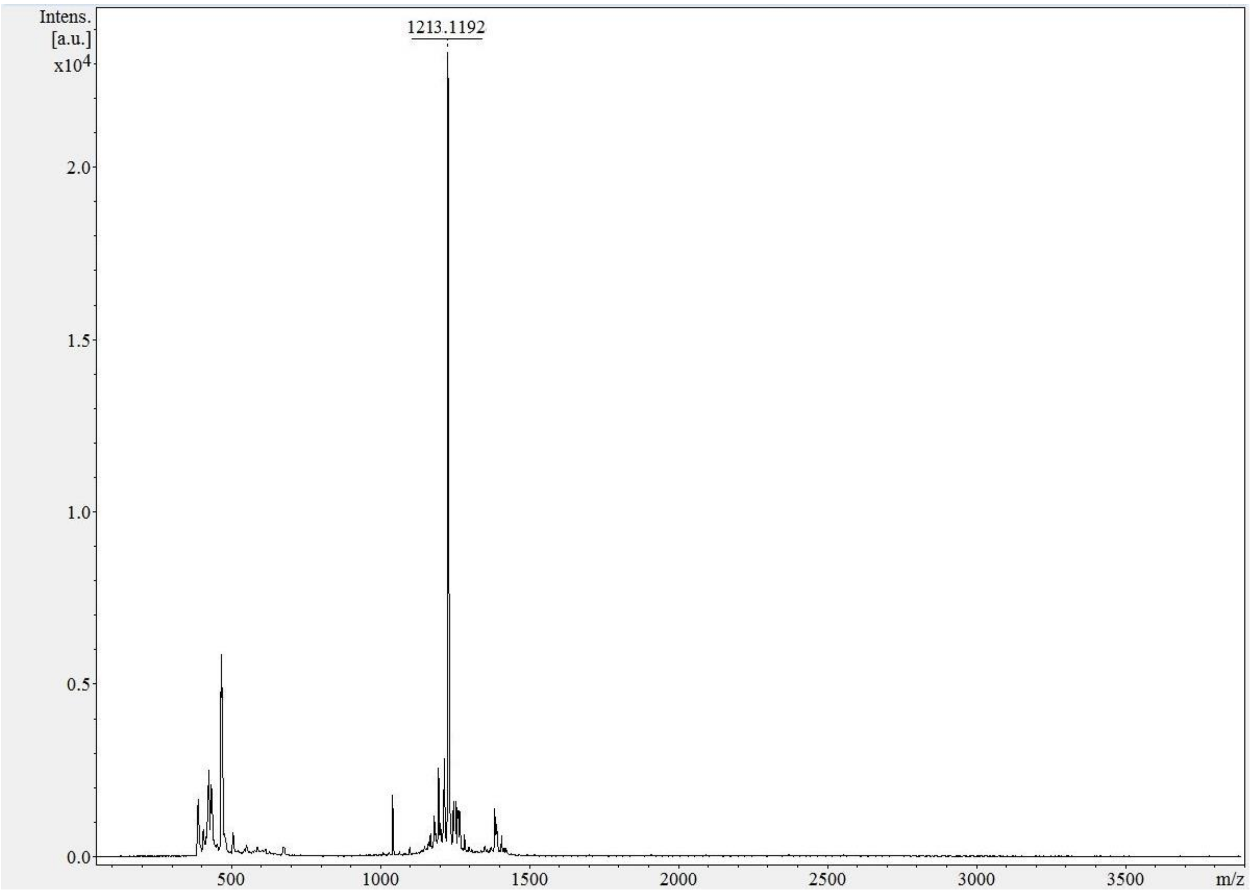


**Figure S5.** MALDI-TOF (m/z) for [$R_3W_4$] **(3b)**.

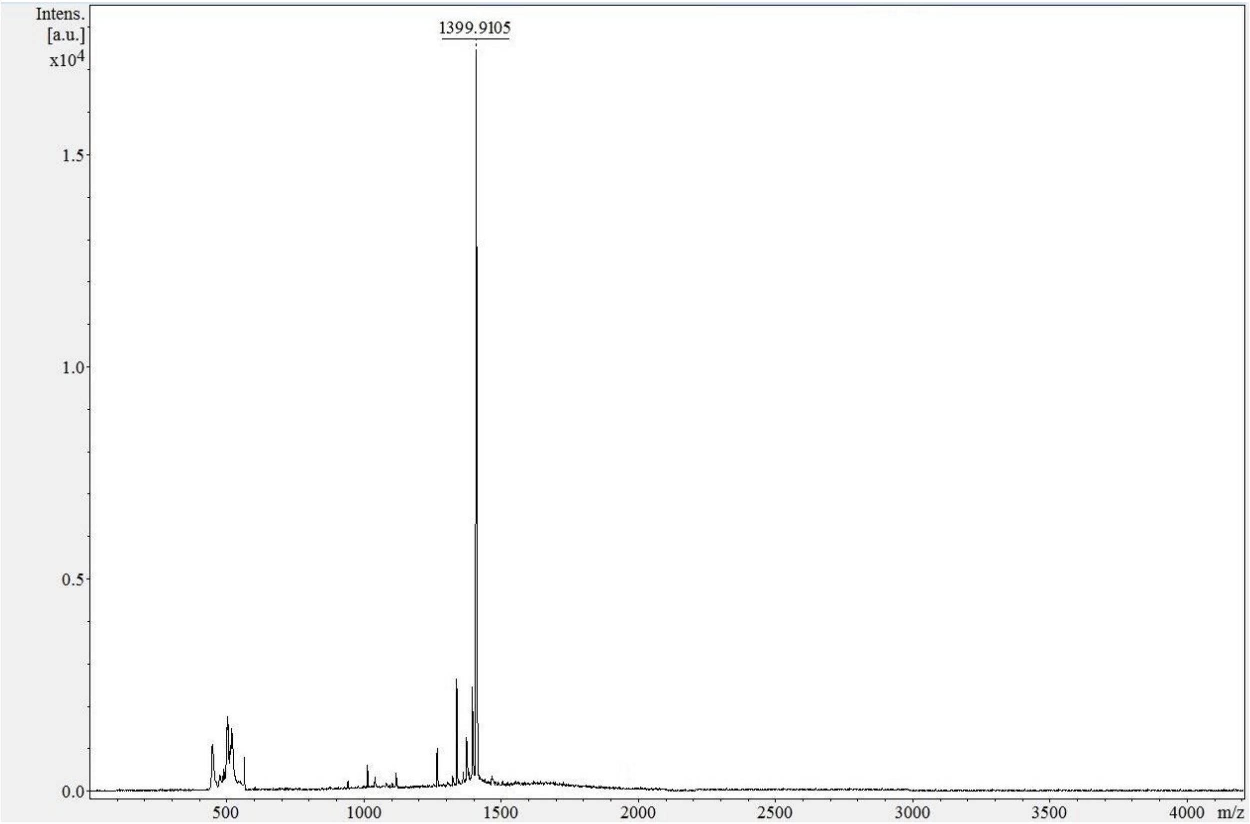


**Figure S6.** MALDI-TOF (m/z) for [$R_3W_5$] (**3c**).

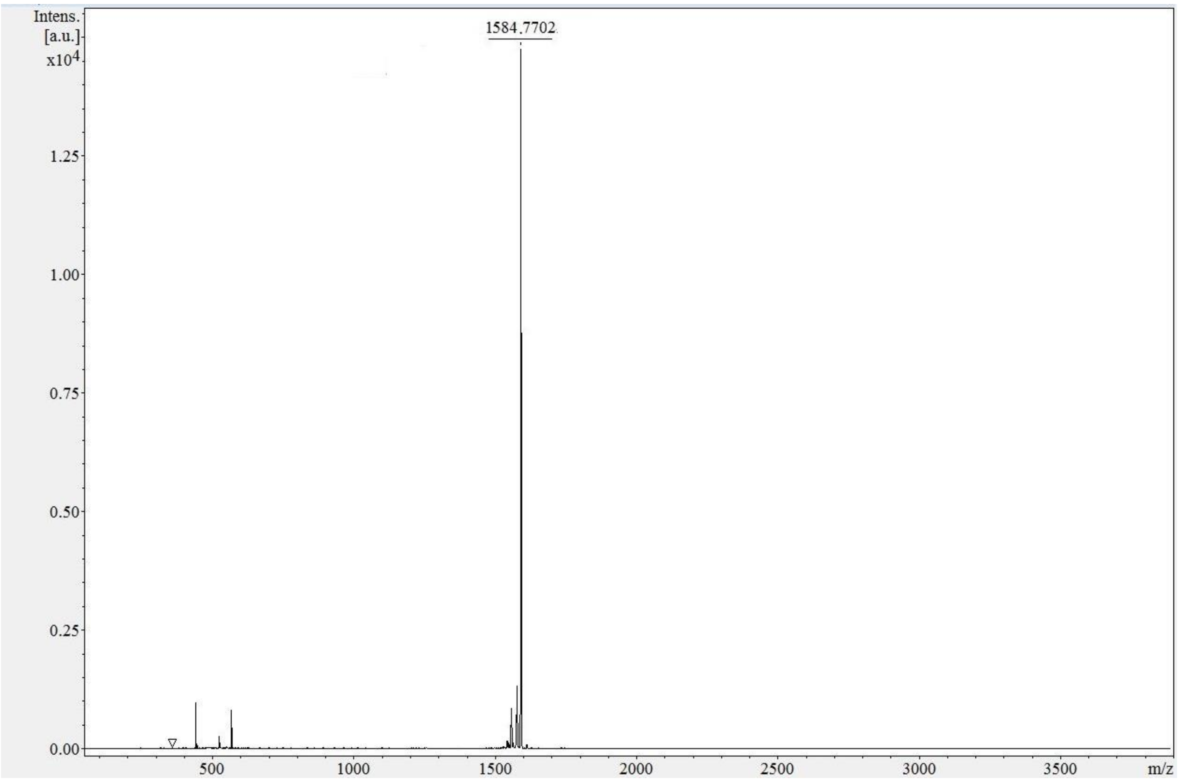


**Figure S7.** MALDI-TOF (m/z) for [$R_3W_6$] **(3d)**.

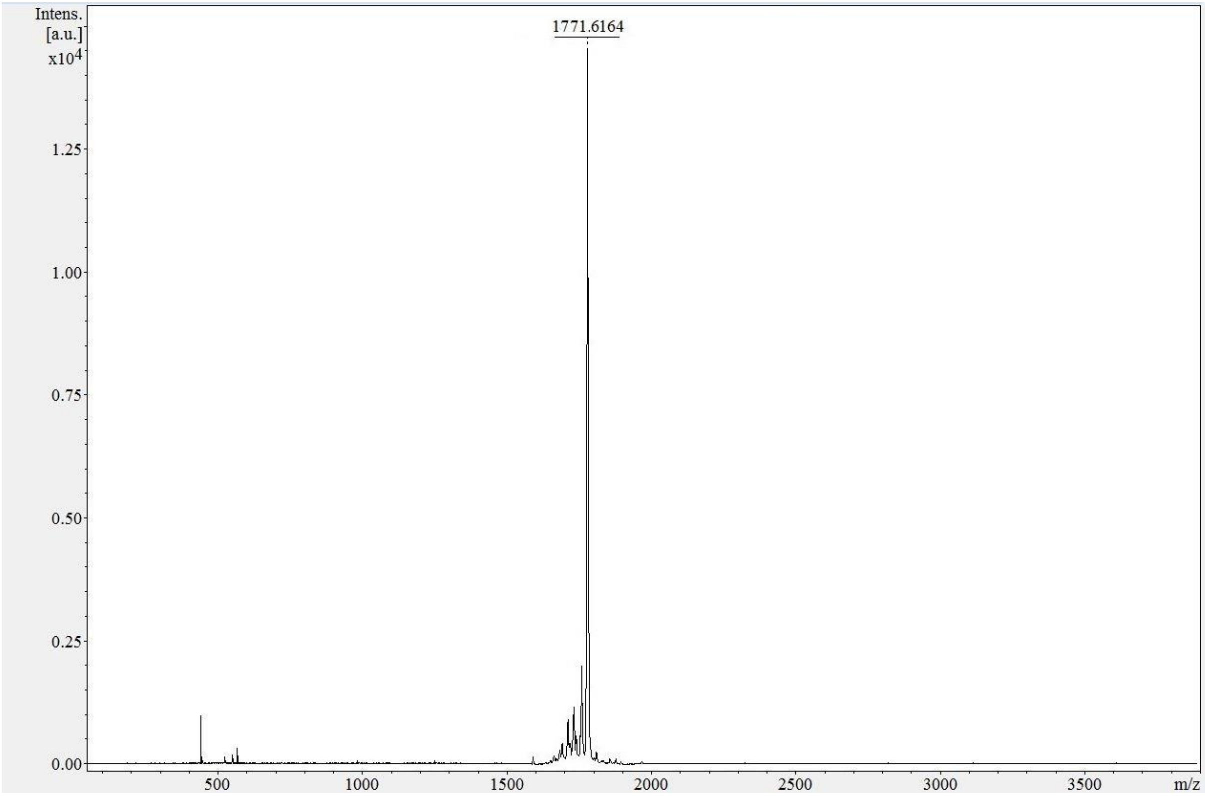


**Figure S8.** MALDI-TOF (m/z) for [$R_3W_7$] **(3e)**.

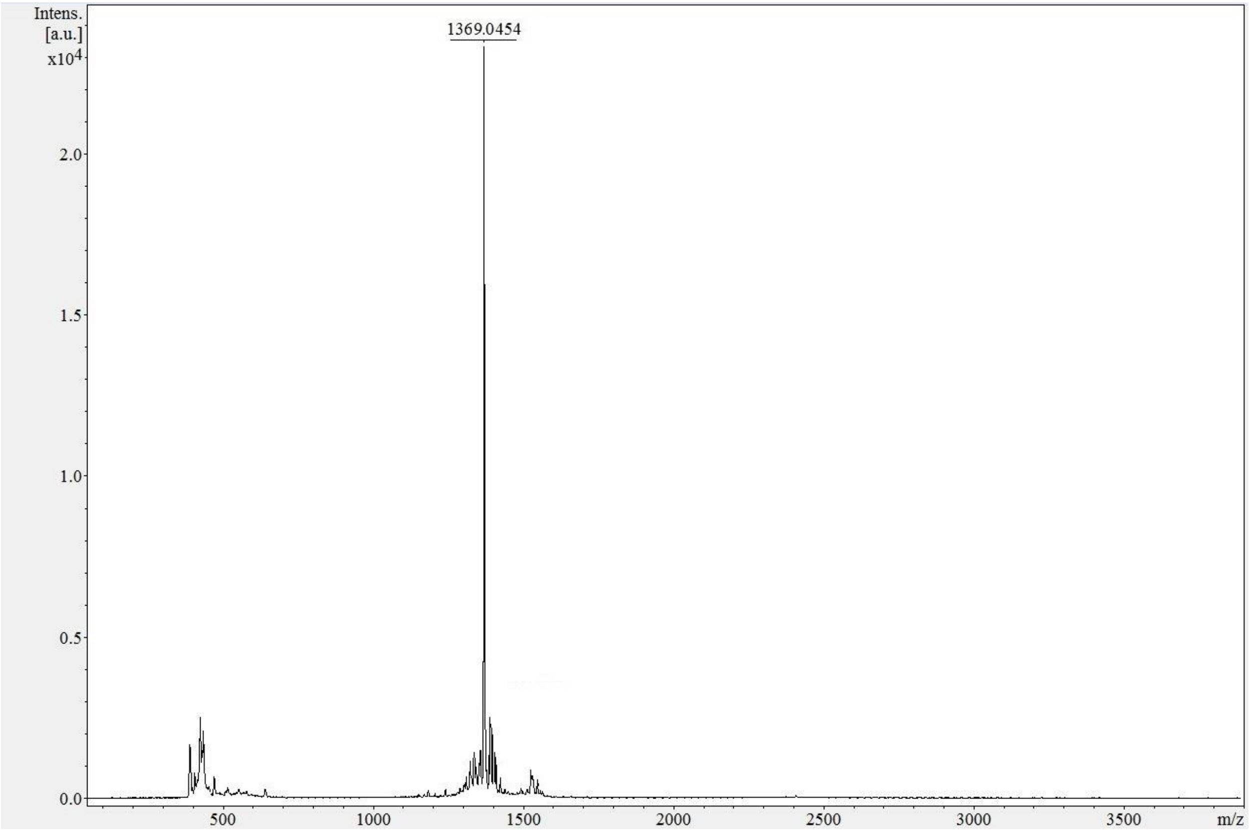


**Figure S9.** MALDI-TOF (m/z) for [$dR_4W_4$] **(4a)**.

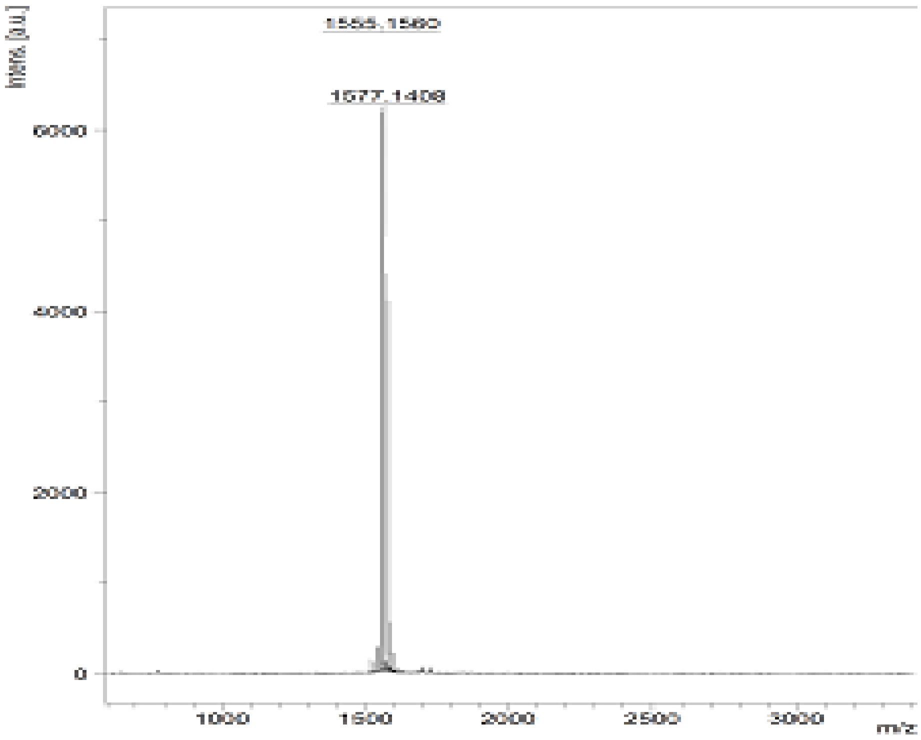


**Figure S10.** MALDI-TOF (m/z) for [$R_4W_5$] **(4b)**.

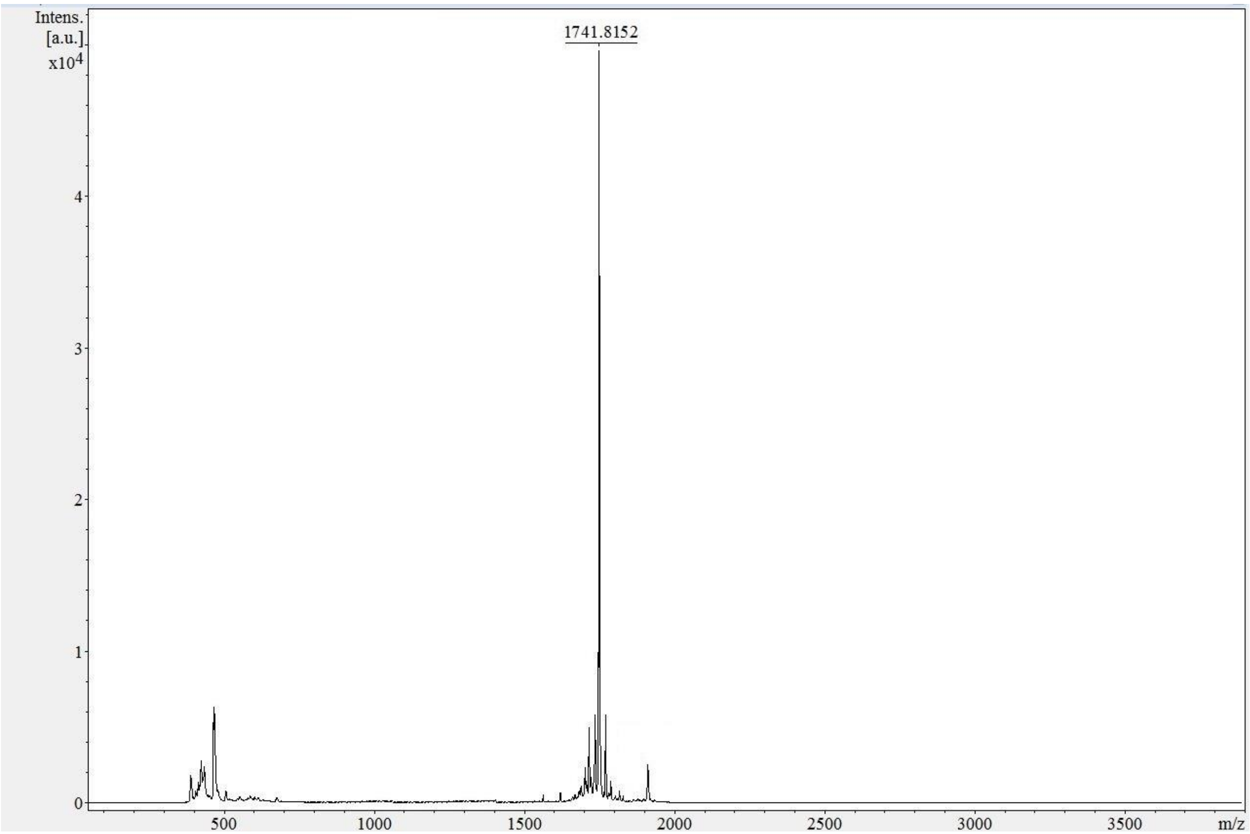


**Figure S11.** MALDI-TOF (m/z) for [$R_4W_6$] **(4c)**.

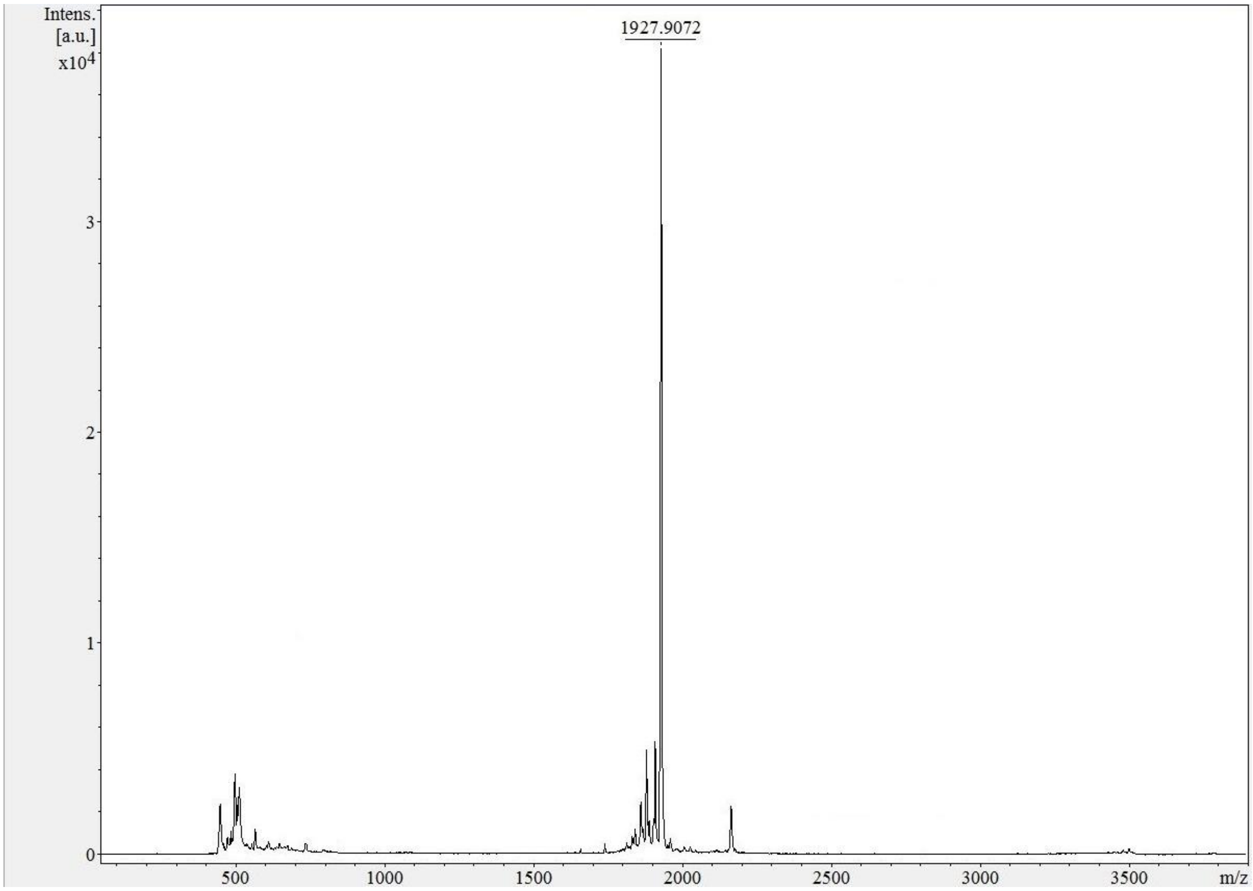


**Figure S12.** MALDI-TOF (m/z) for [$R_4W_7$] **(4d)**.

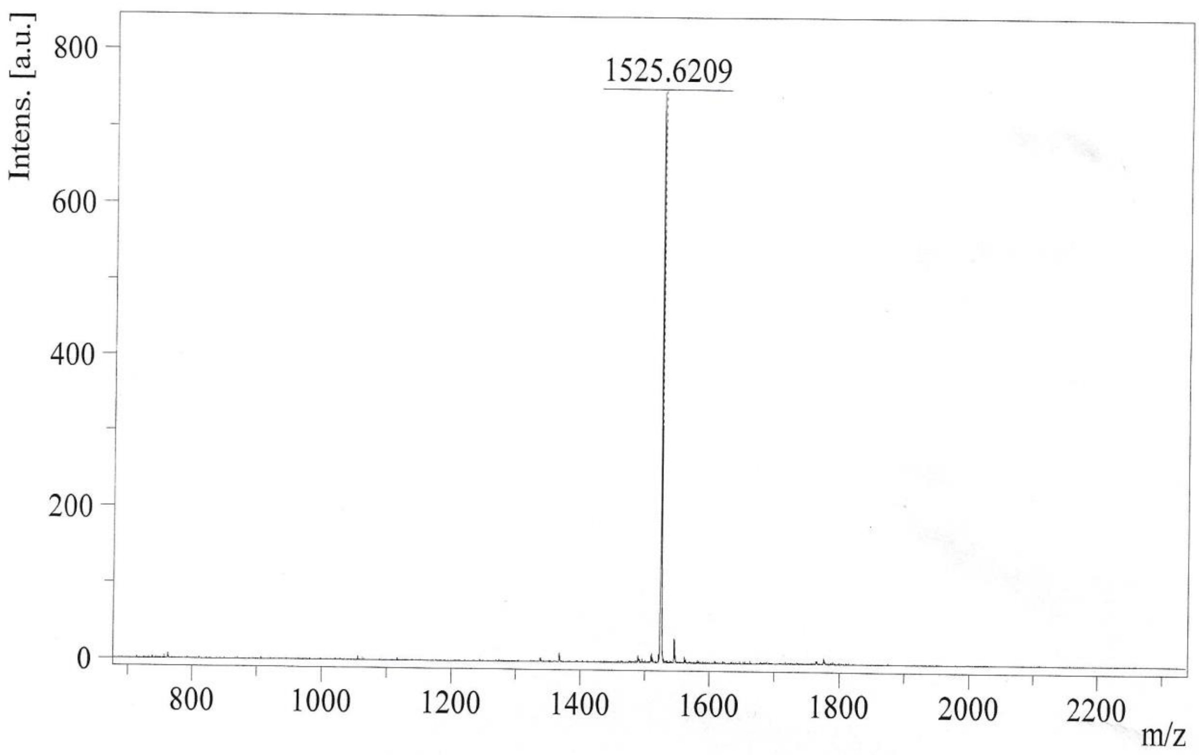


**Figure S13.** MALDI-TOF (m/z) for [$R_5W_4$] **(5a)**.

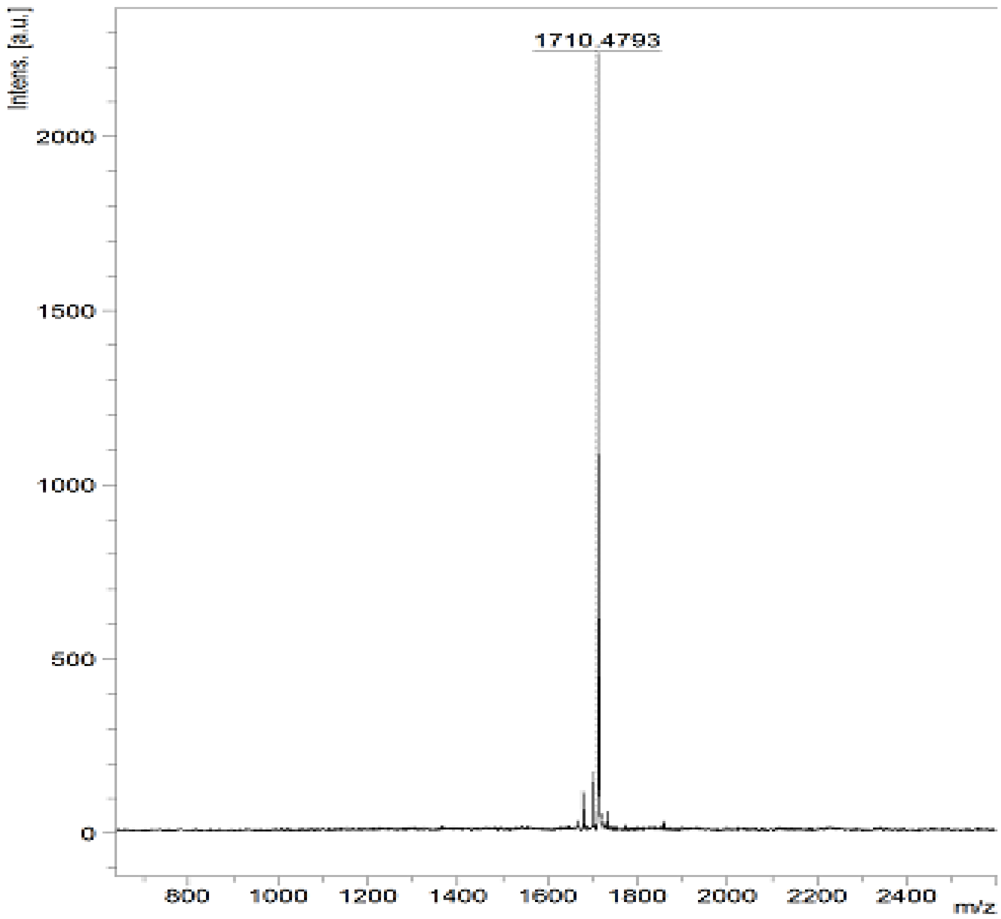


**Figure S14.** MALDI-TOF (m/z) for [$R_5W_5$] **(5b)**.

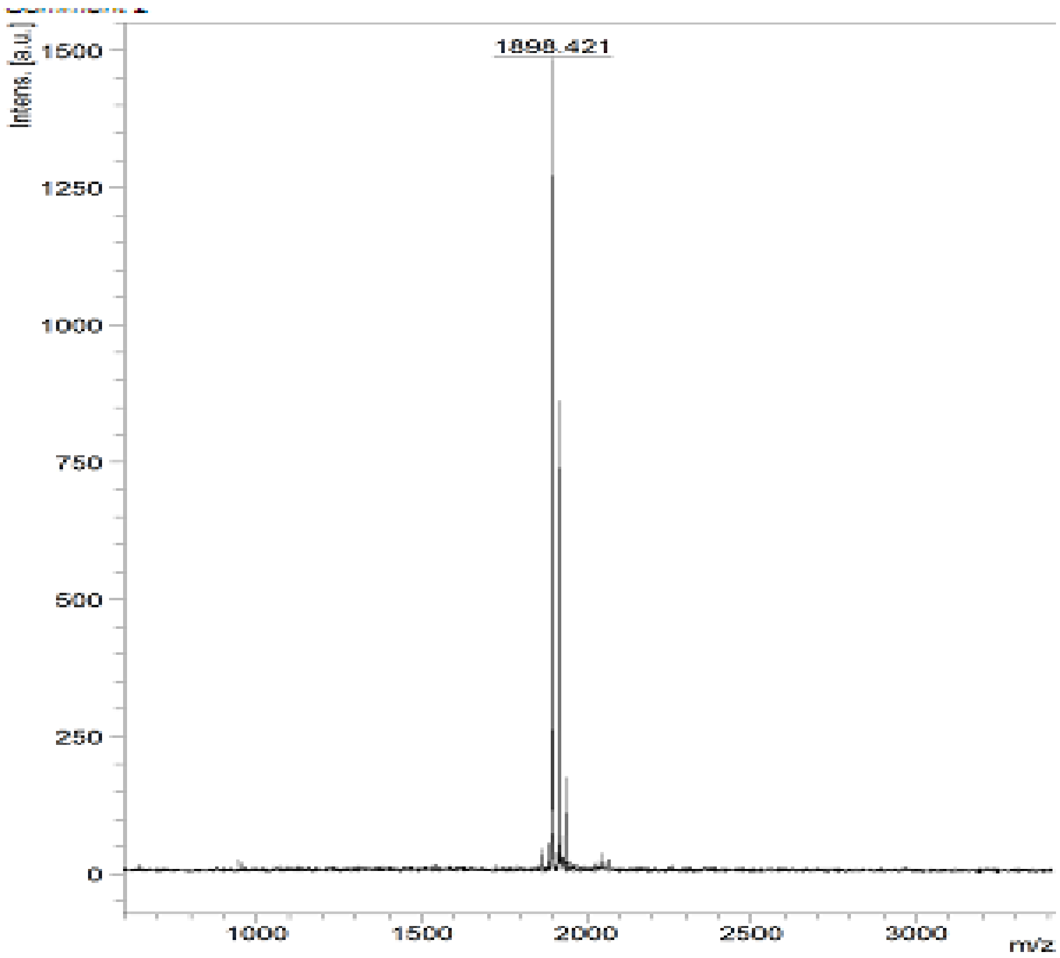


**Figure S15.** MALDI-TOF (m/z) for [$R_5W_6$] (**5c**).

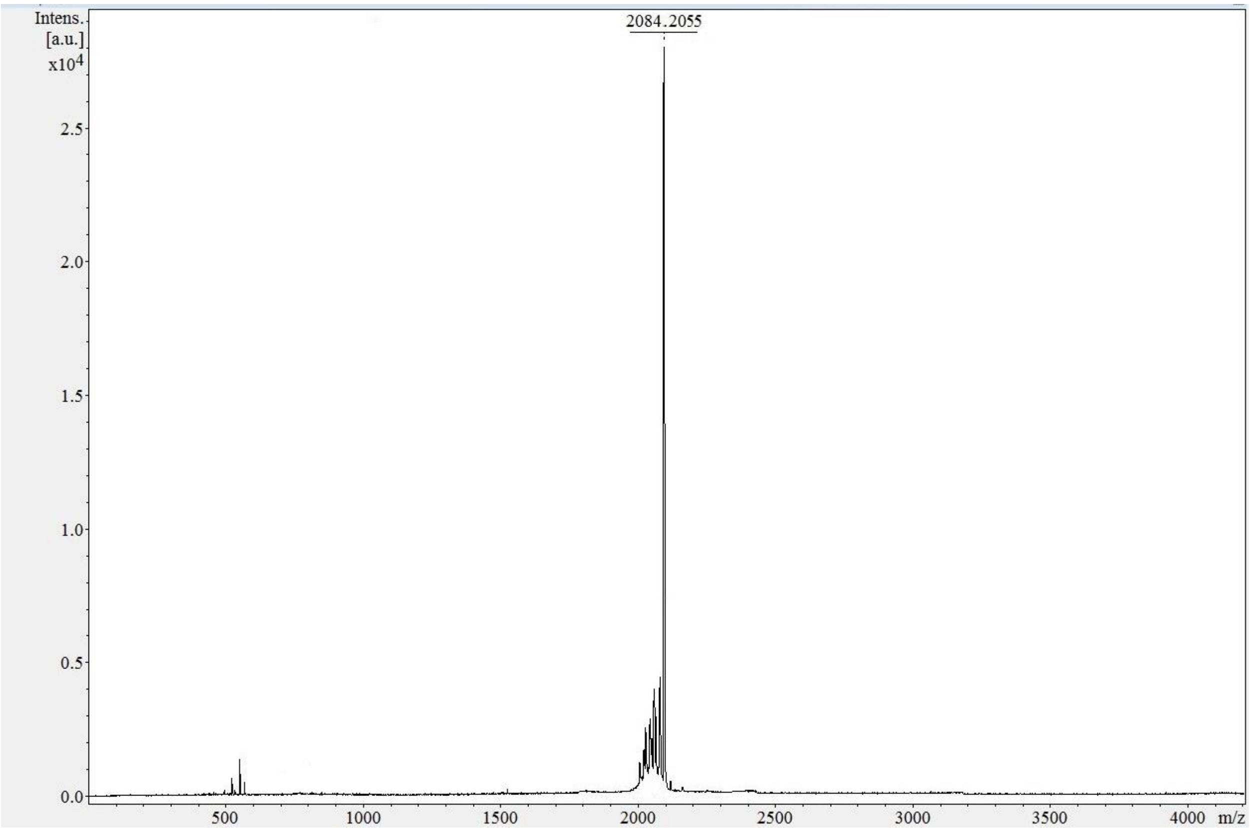


**Figure S16.** MALDI-TOF (m/z) for [$R_5W_7$] **(5d)**.

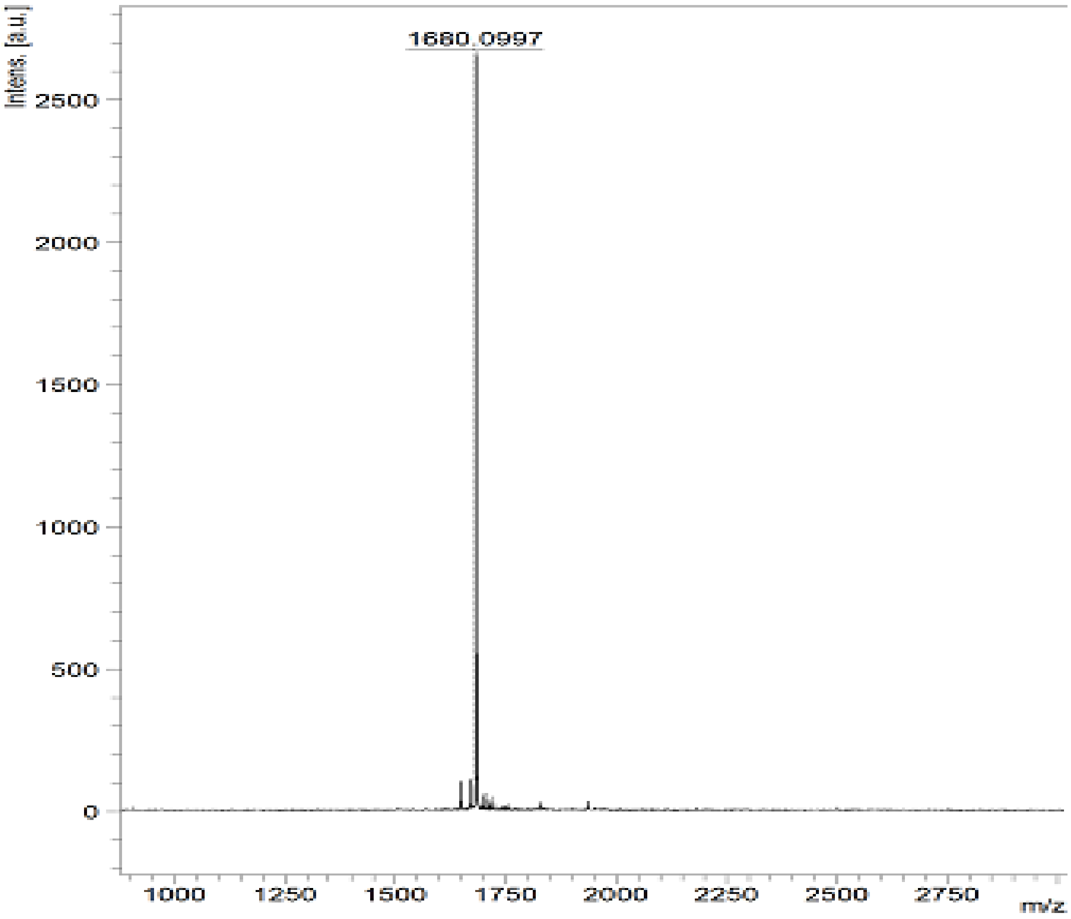


**Figure S17.** MALDI-TOF (m/z) for [$R_6W_4$] (**6a**).

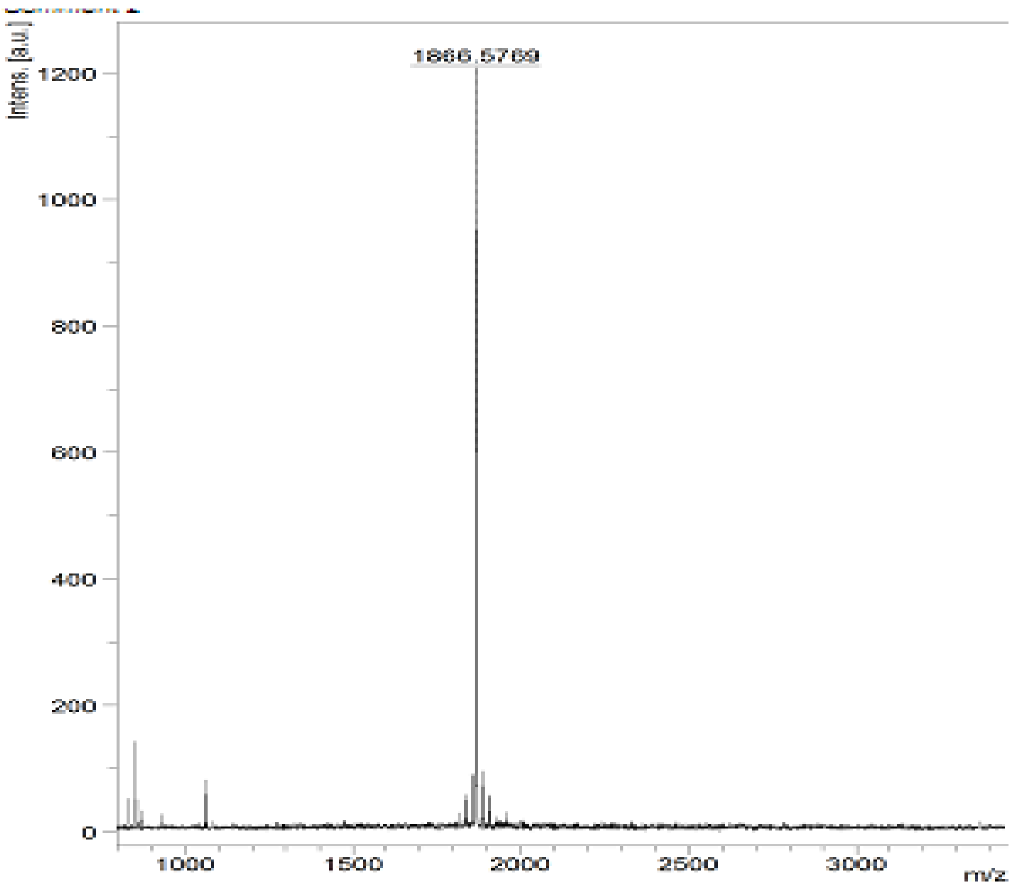


**Figure S18.** MALDI-TOF (m/z) for [$R_6W_5$] (**6b)**.

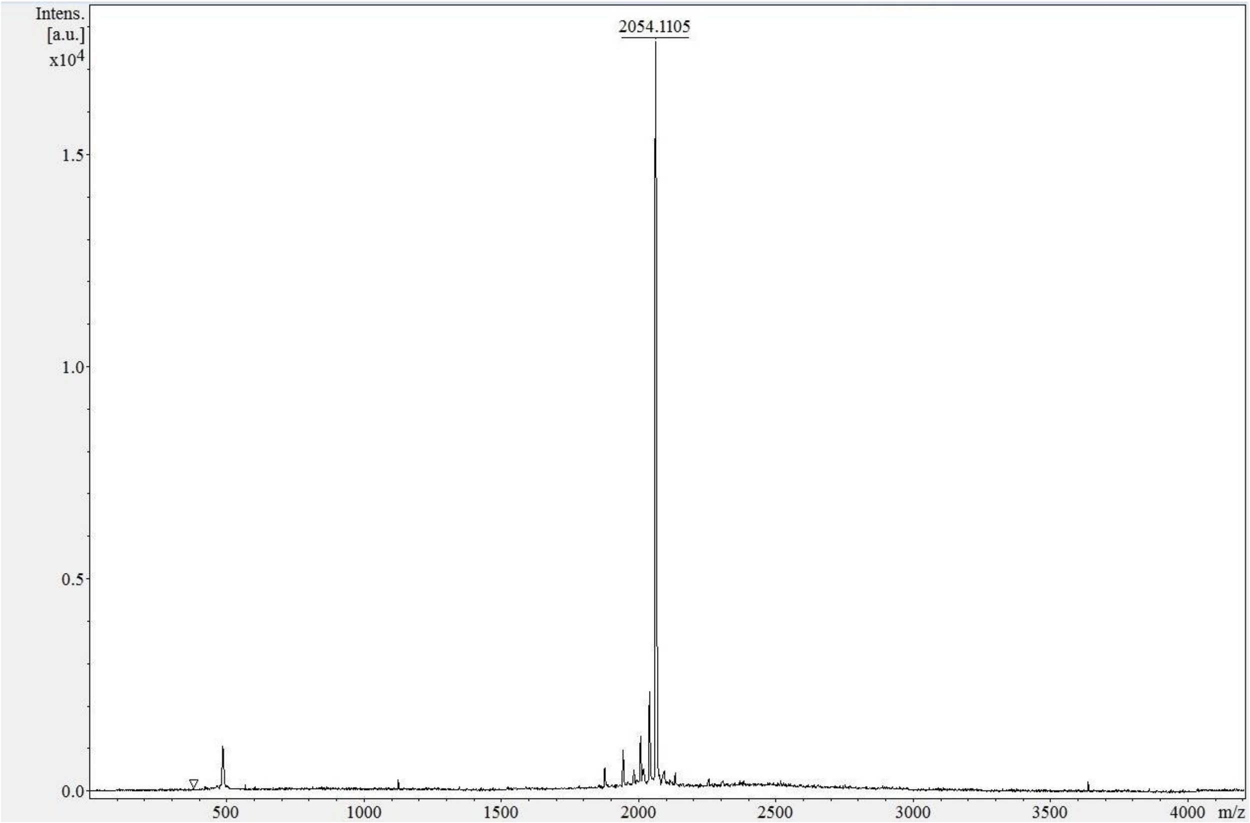


**Figure S19.** MALDI-TOF (m/z) for [$R_6W_6$] **(6c)**.

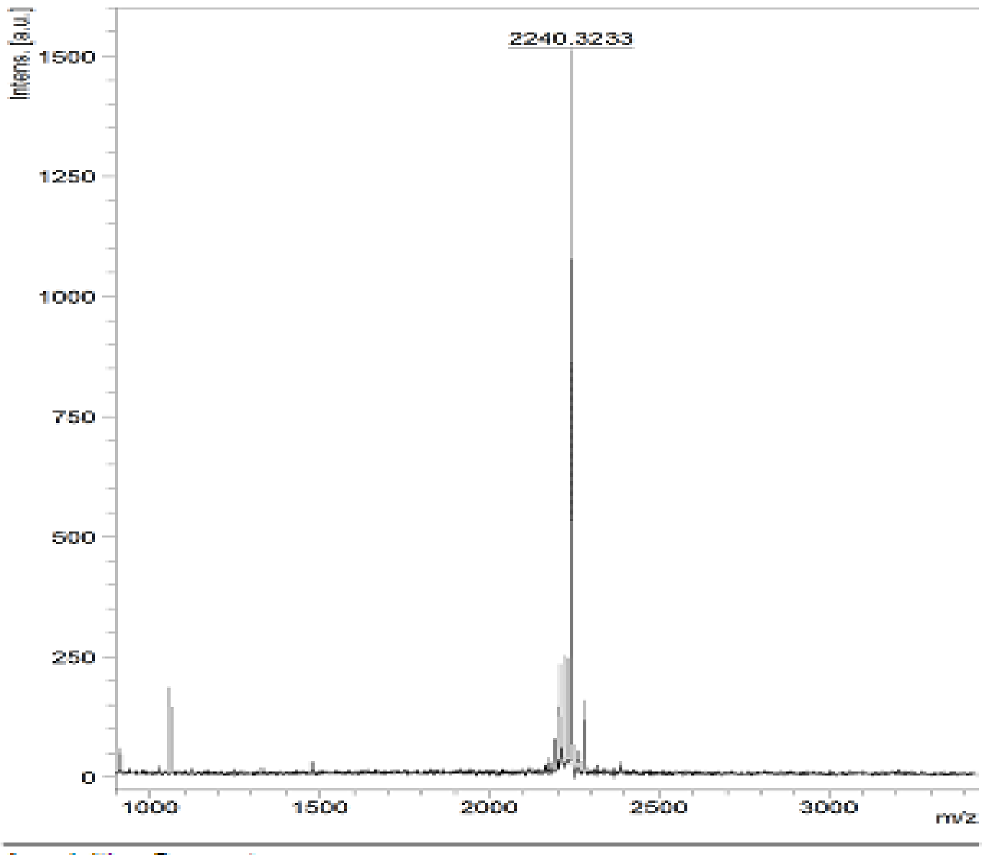


**Figure S20.** MALDI-TOF (m/z) for [$R_6W_7$] **(6d)**.

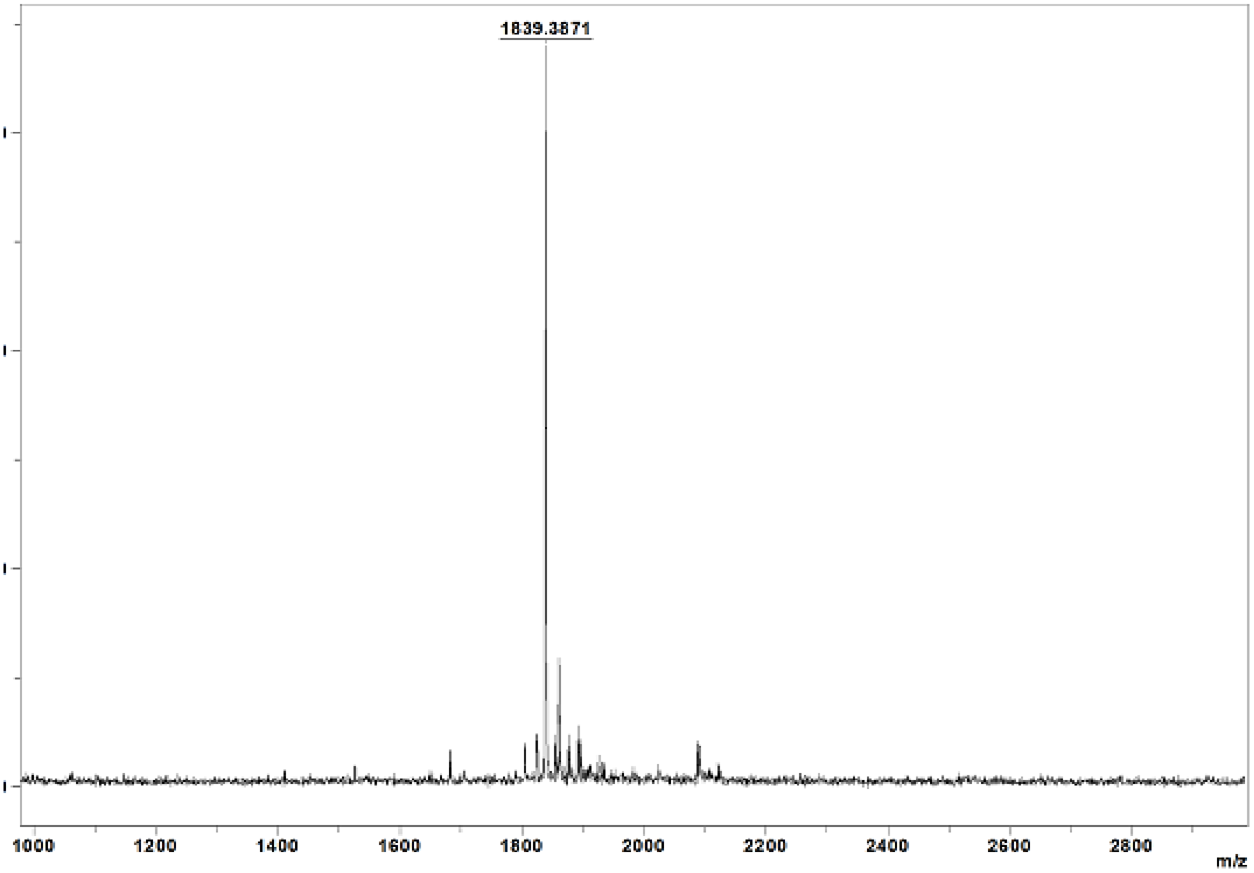


**Figure S21.** MALDI-TOF (m/z) for [$R_7W_4$] **(7a)**.

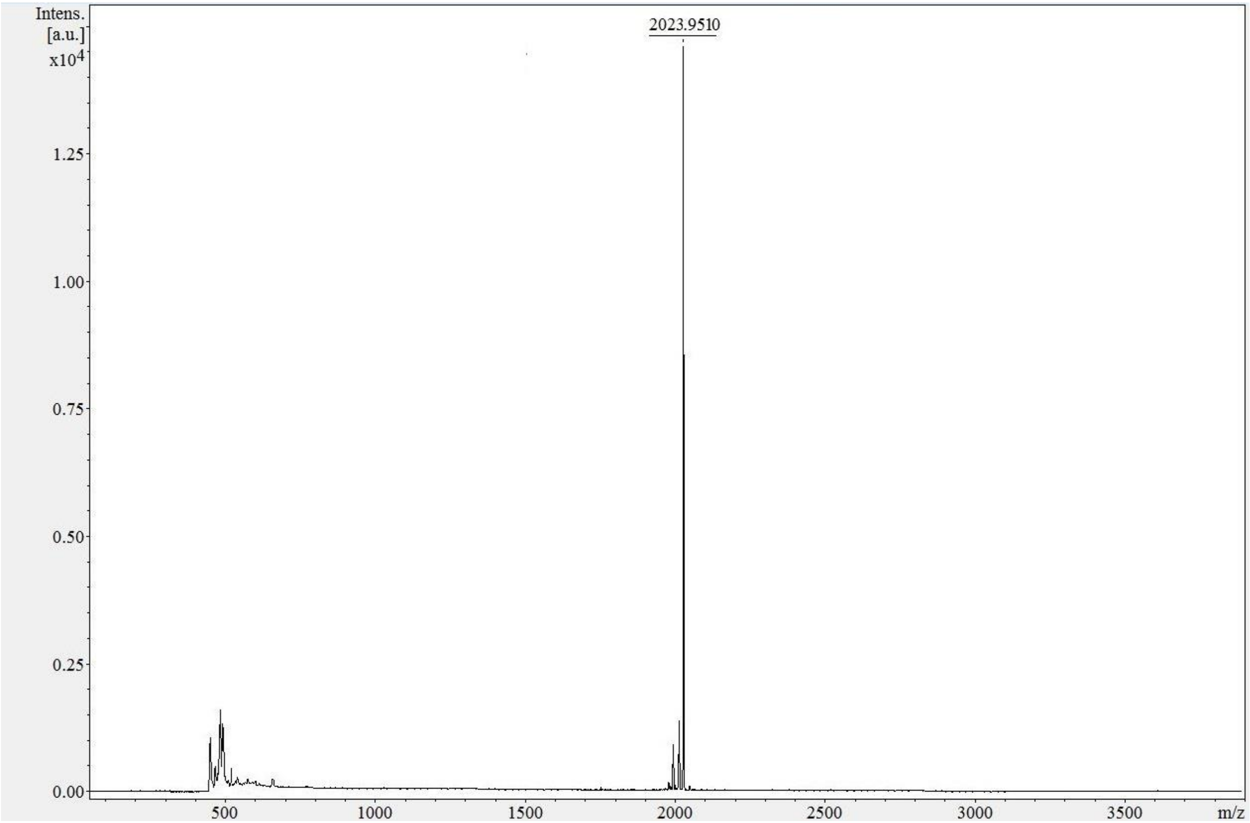


**Figure S22.** MALDI-TOF (m/z) for [$R_7W_5$] (**7b**).

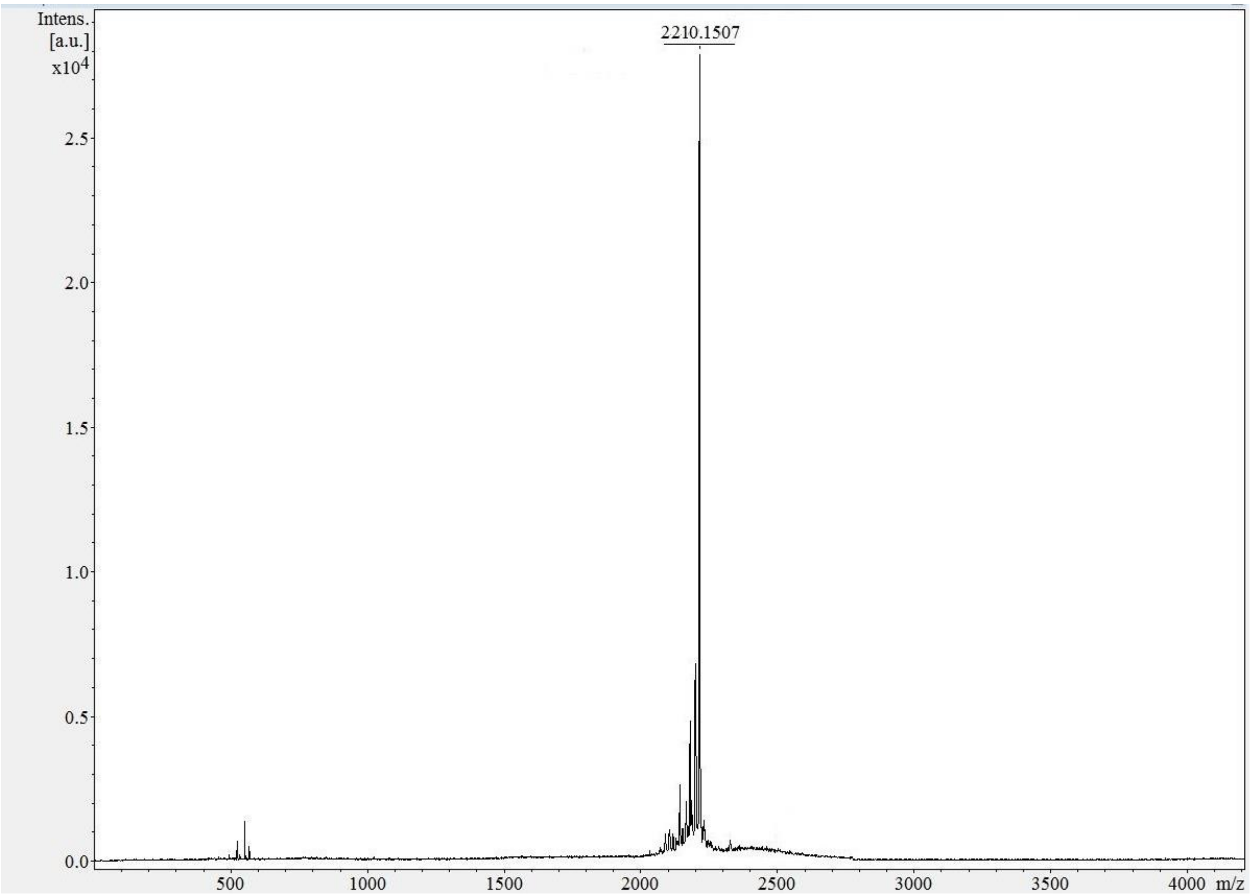


**Figure S23.** MALDI-TOF (m/z) for [$R_7W_6$] **(7c)**.

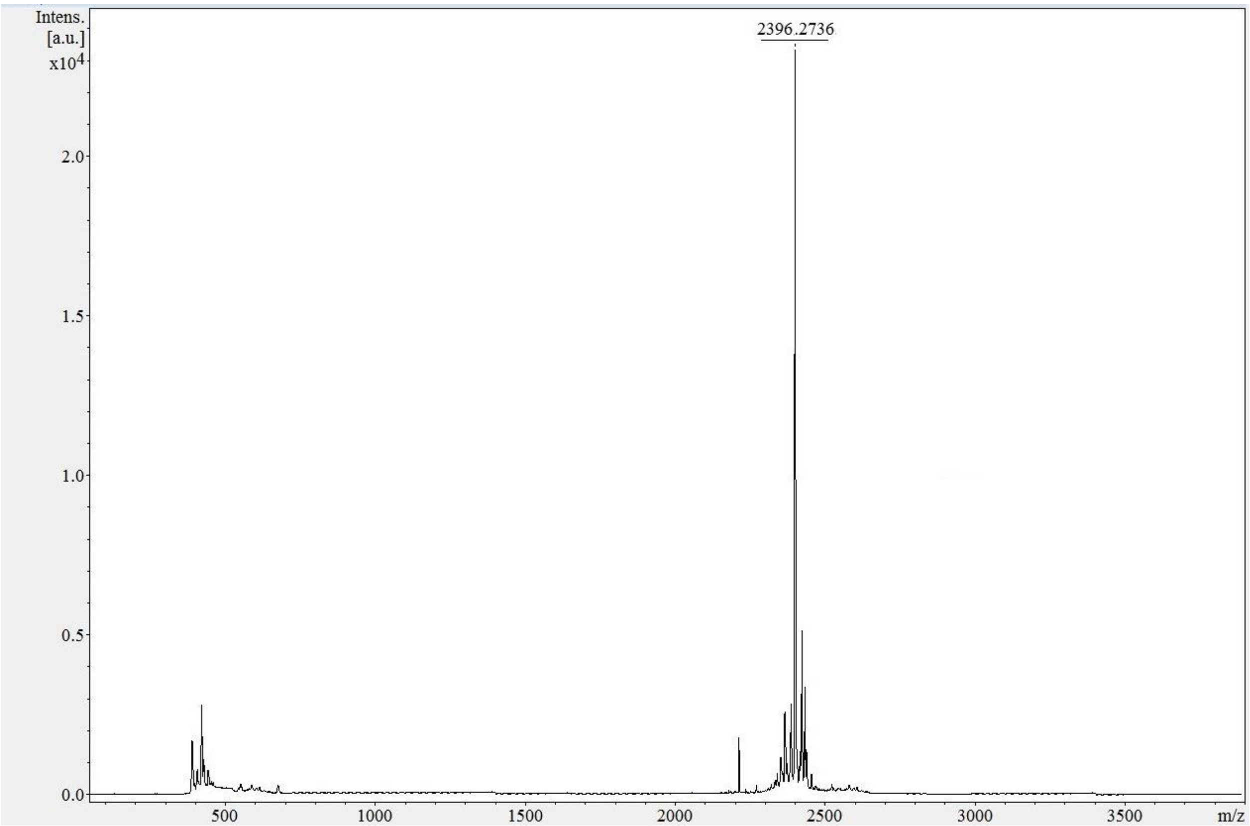

**Figure S24.** MALDI-TOF (m/z) for [$R_7W_7$] **(7d)**.

**HPLC Analysis of Peptides**

The purity of cyclic peptides was determined using reverse-phase analytical HPLC (Shimadzu; LC-20ADXR). The conditions used to conduct the purity analysis are provided below:

**Method**

**Column:** Phenomenex (Luna), 4 µm, C18, 150 × 4.6 mm
**Flow rate:** 0.5 mL/min
**Mobile Phase**: Buffer A-Water containing 0.1% TFA,
Buffer B-Acetonitrile containing 0.1% TFA
**Gradient**

| Time (min) | Buffer B (%) |
|---|---|
| 0.00 | 2 |
| 2 | 2 |
| 5 | 30 |
| 14 | 75 |
| 15 | 95 |
| 17 | 95 |
| 20 | 2 |

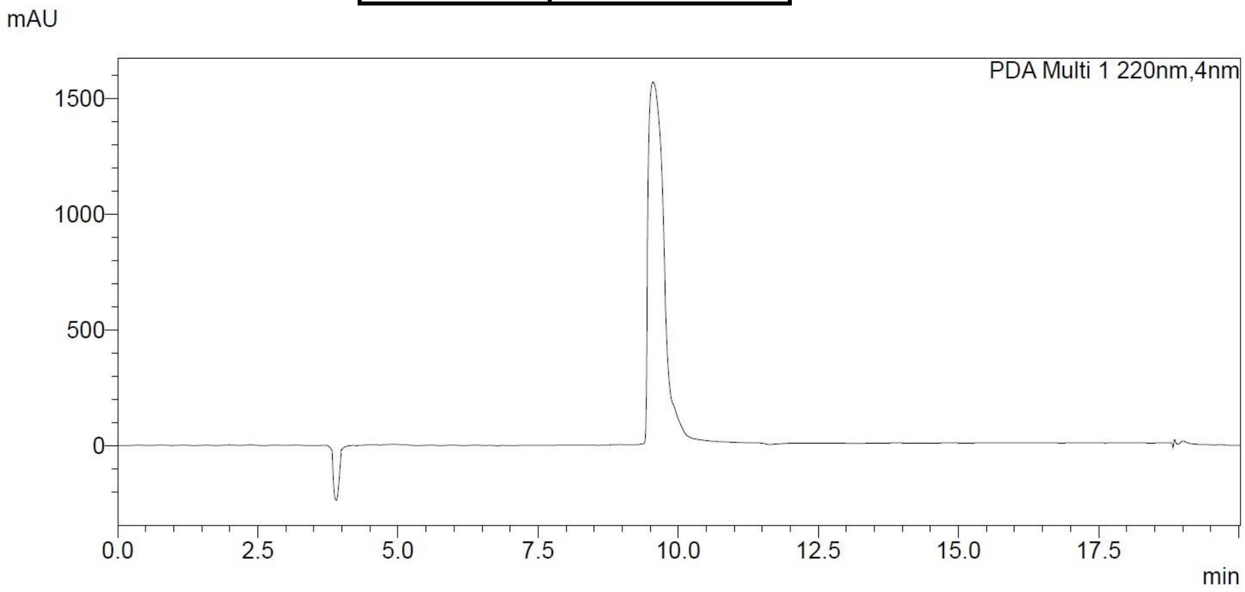


**Figure S25.** HPLC analysis of compound **2a**.

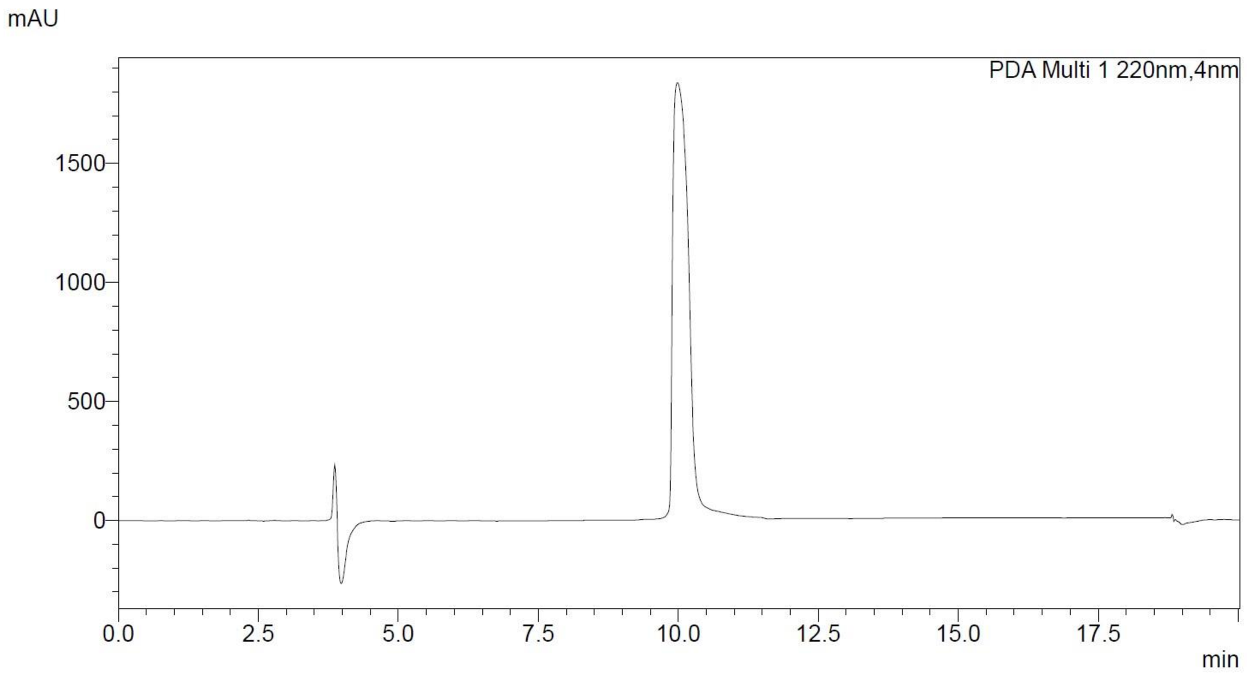


**Figure S26.** HPLC analysis of compound **2b**.

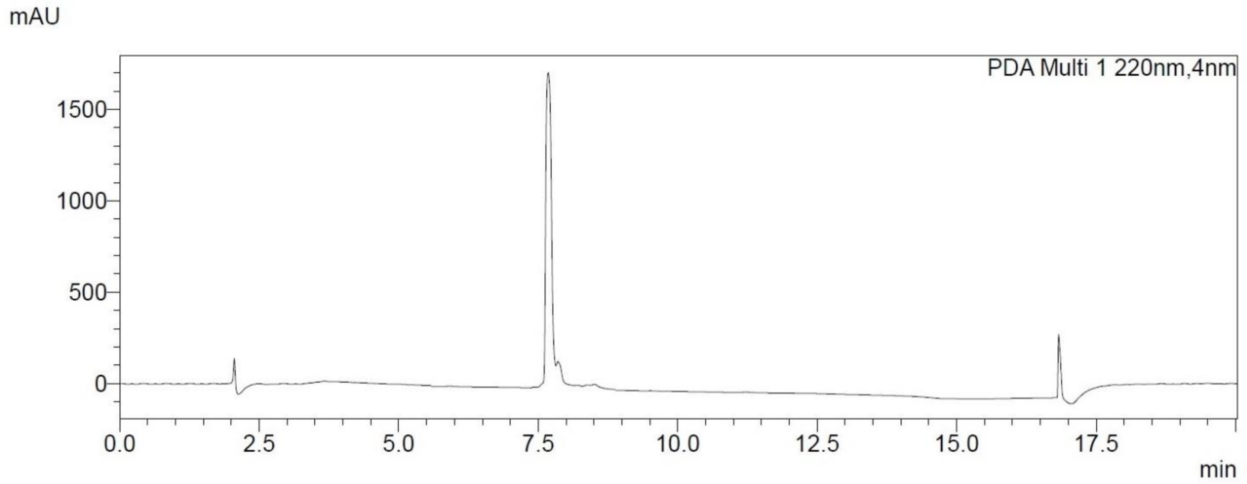


**Figure S27.** HPLC analysis of compound **3a**.

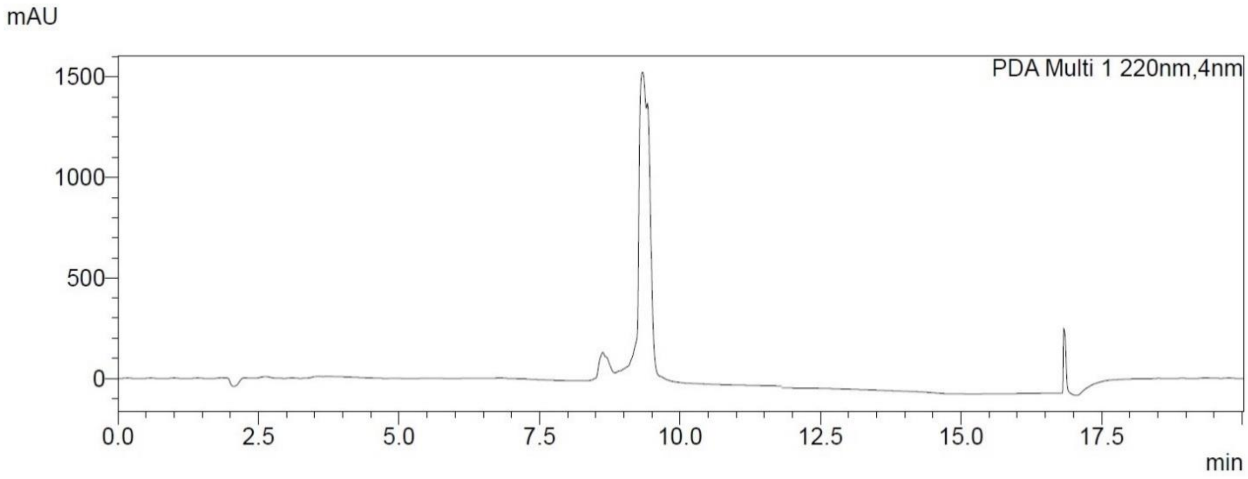


**Figure S28.** HPLC analysis of compound **3b**.

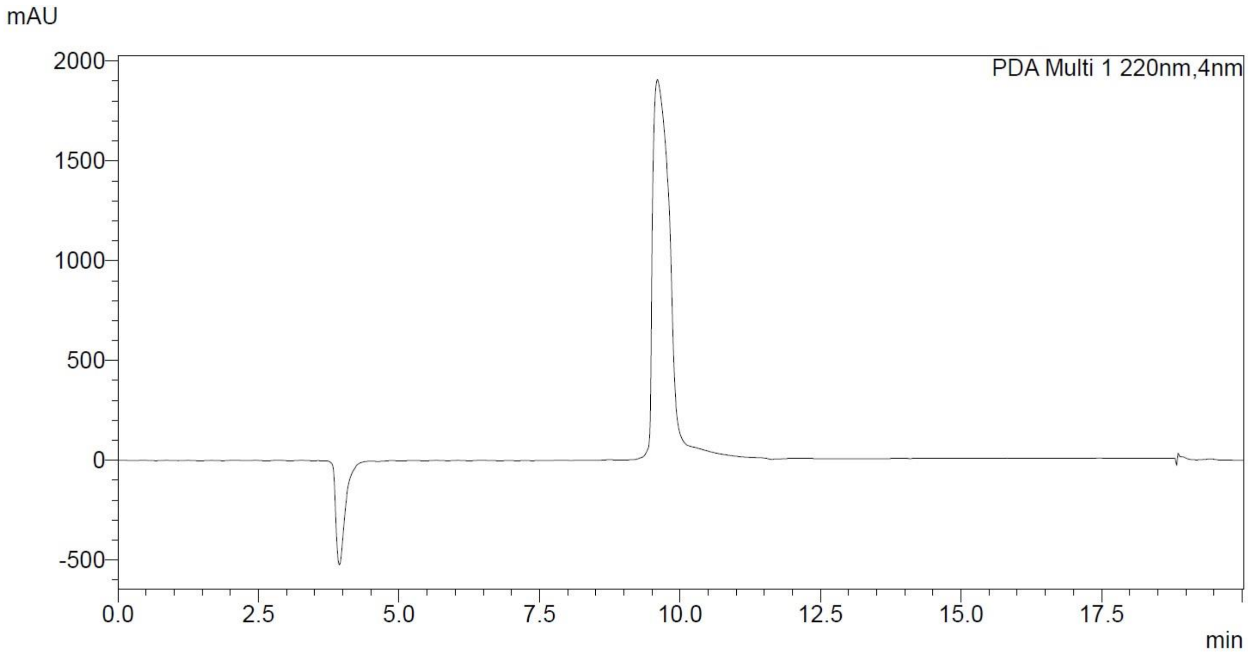


**Figure S29.** HPLC analysis of compound **3c**.

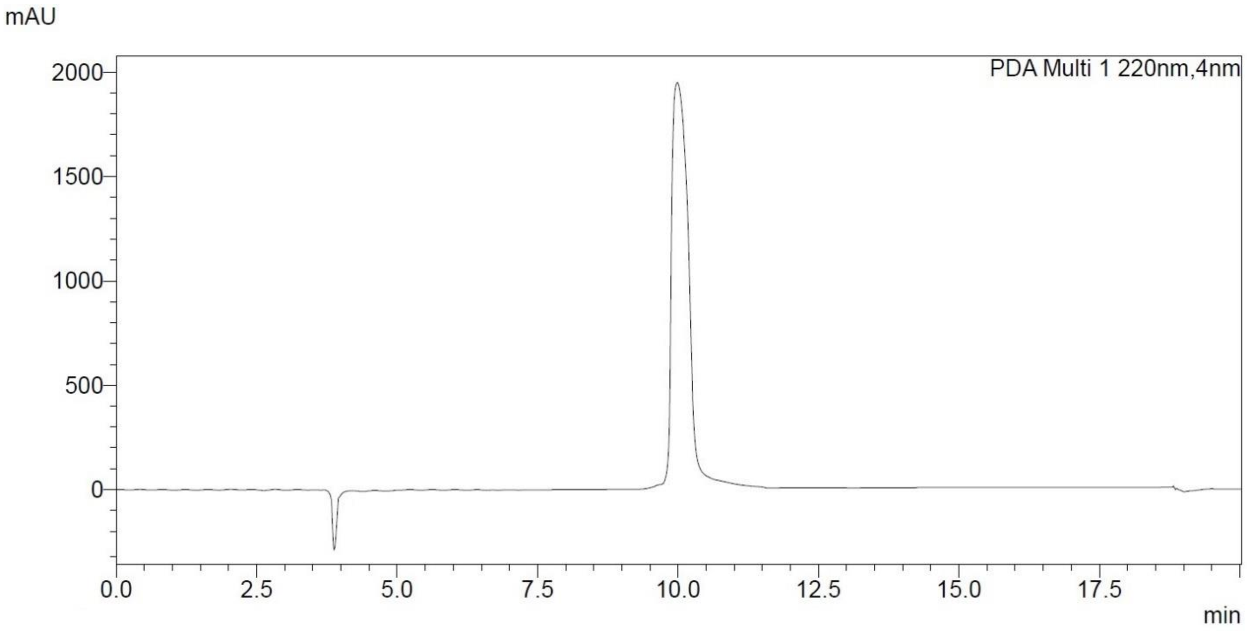


**Figure S30.** HPLC analysis of compound **3d**.

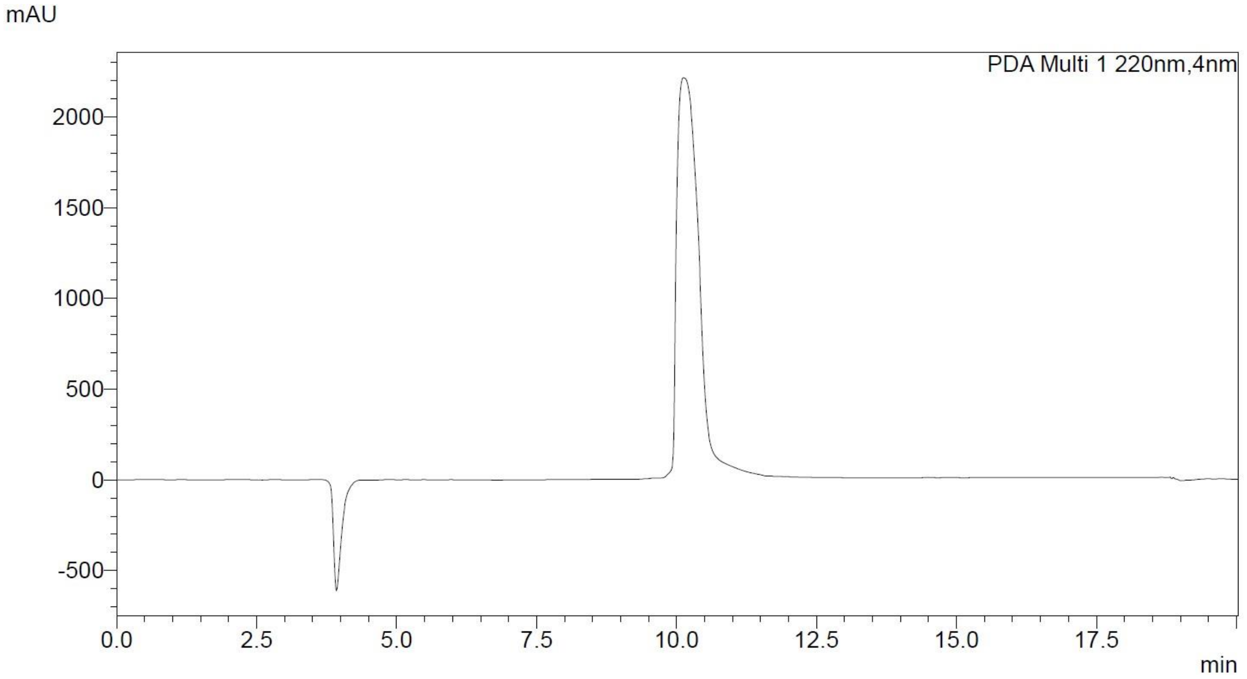


**Figure S31.** HPLC analysis of compound **3e**.

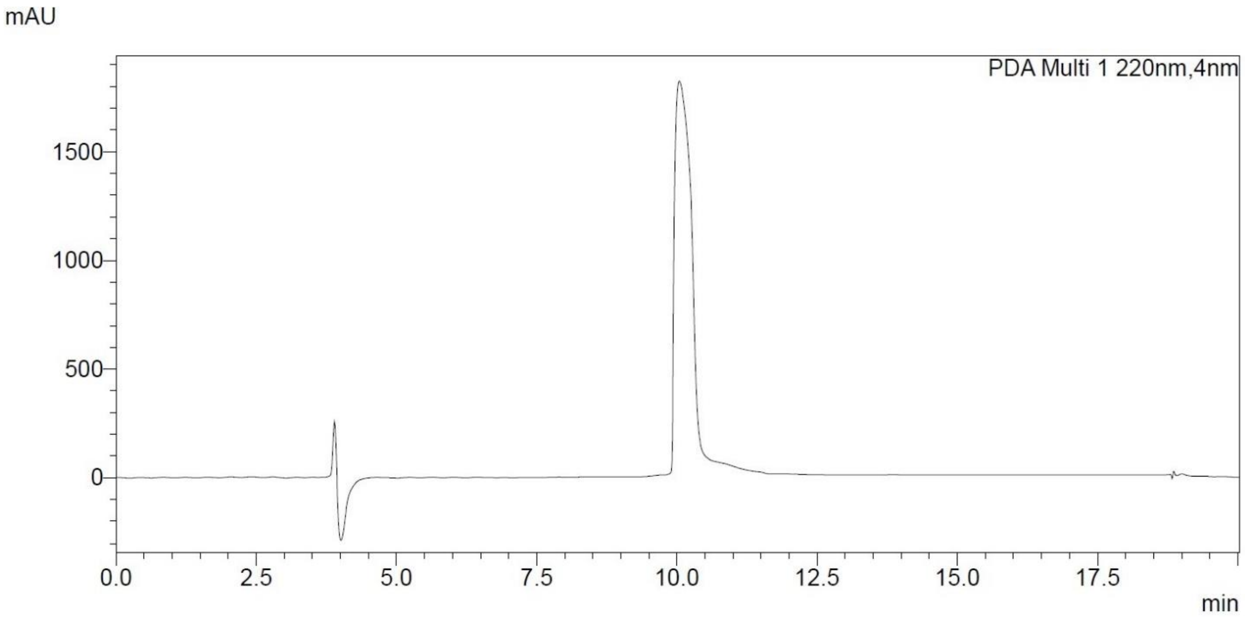


**Figure S32.** HPLC analysis of compound **4a**.

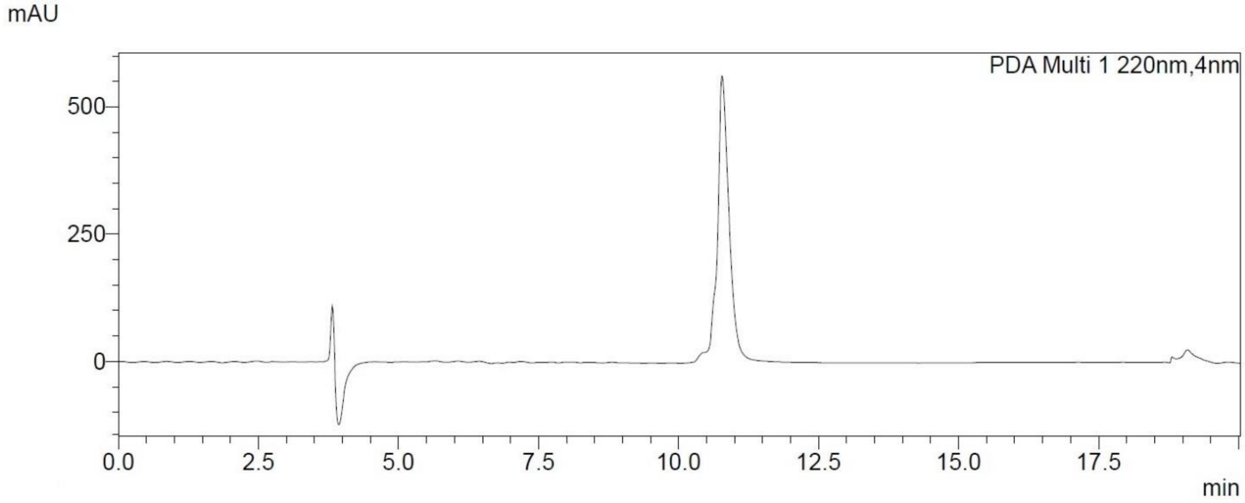


**Figure S33.** HPLC analysis of compound **4b**.

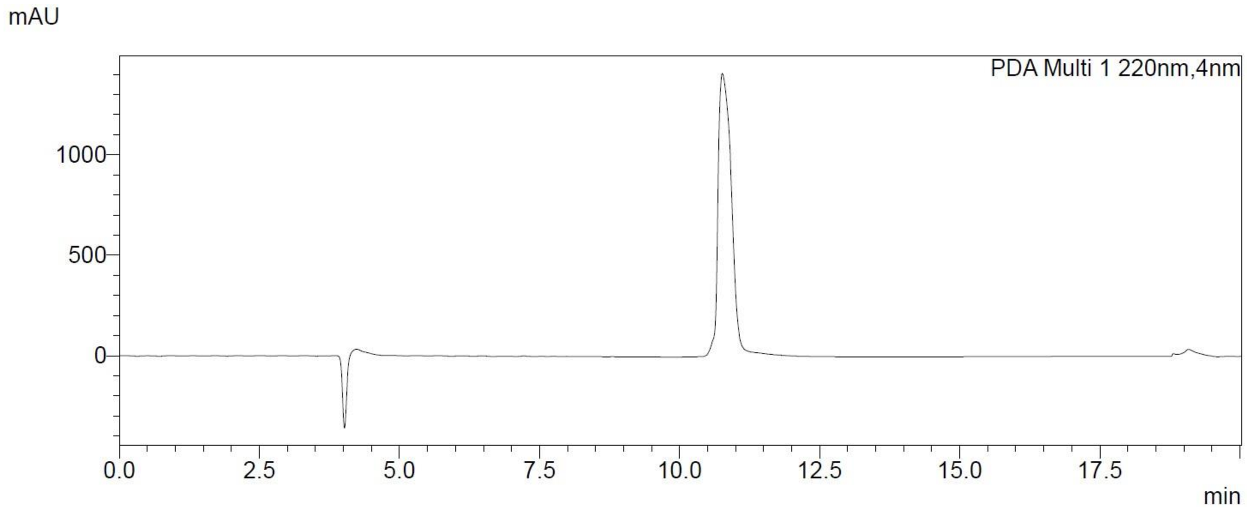


**Figure S34.** HPLC analysis of compound **4c**.

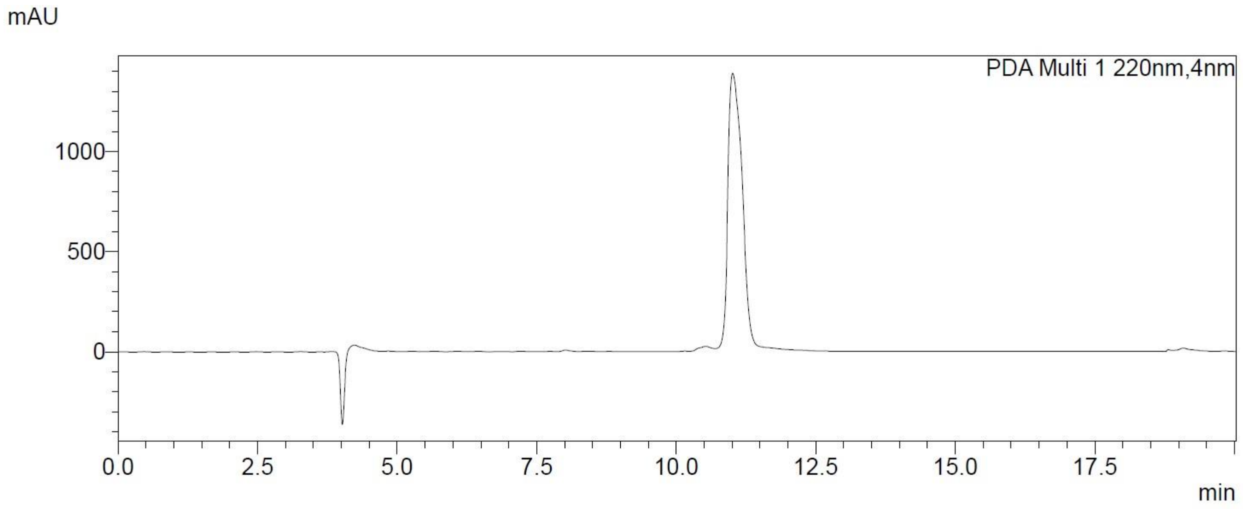


**Figure S35.** HPLC analysis of compound **4d**.

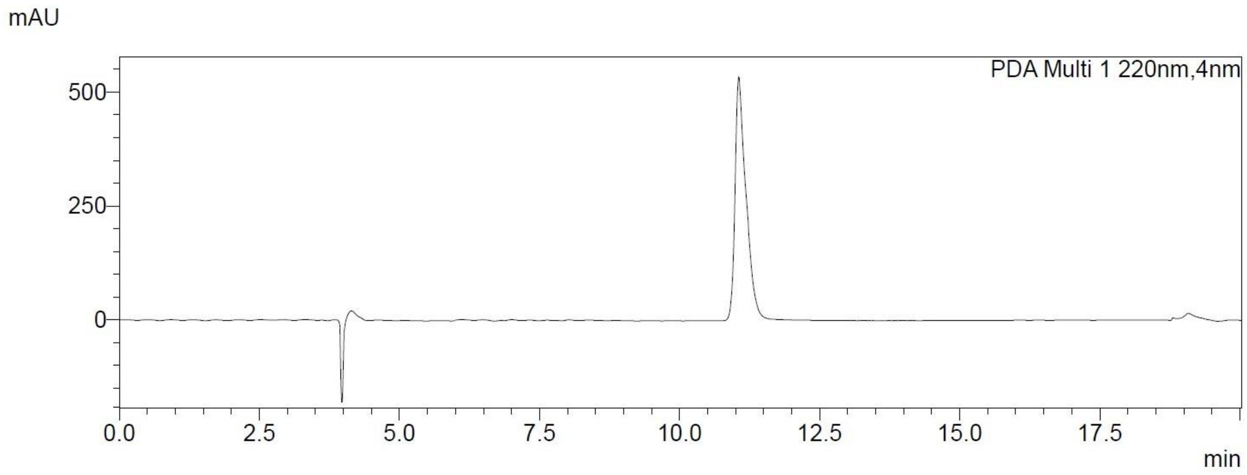


**Figure S36.** HPLC analysis of compound **5d**.

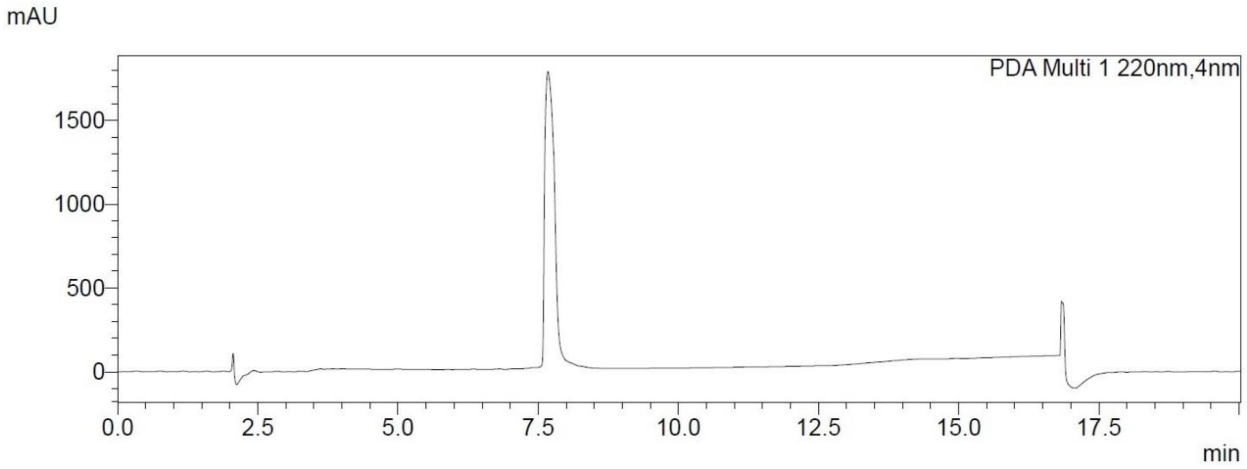


**Figure S37.** HPLC analysis of compound **6a**.

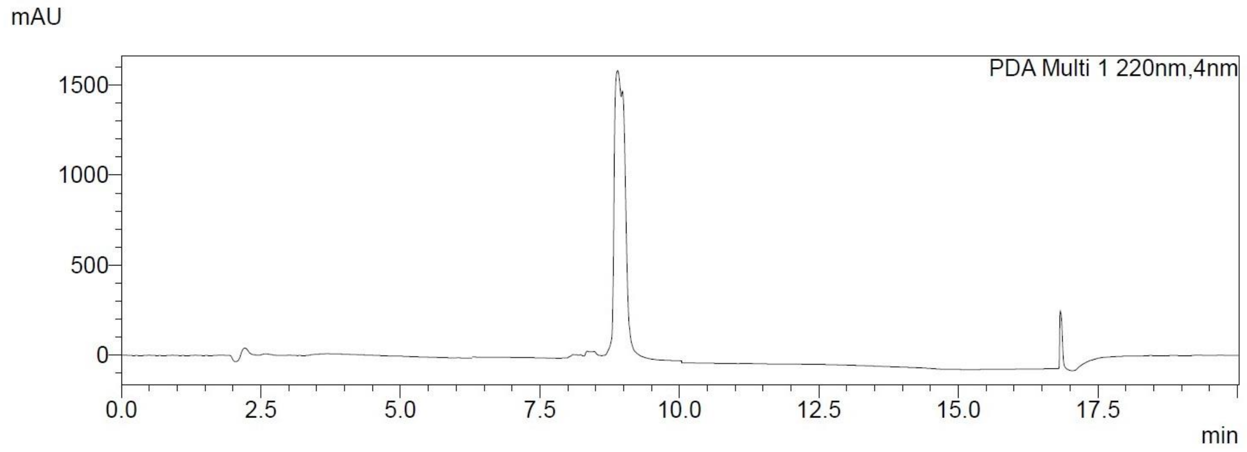


**Figure S38.** HPLC analysis of compound **6b**.

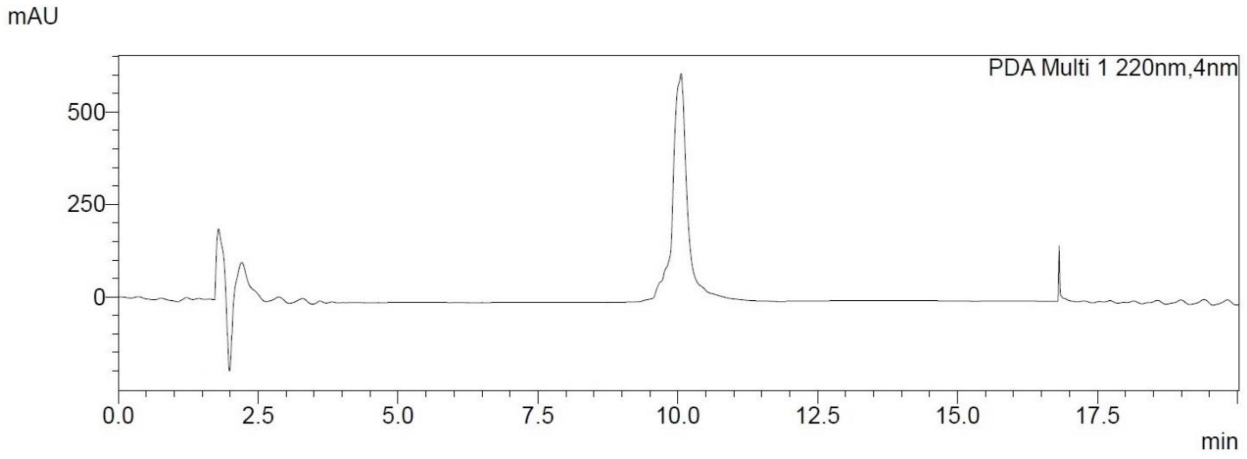


**Figure S39.** HPLC analysis of compound **6c**.

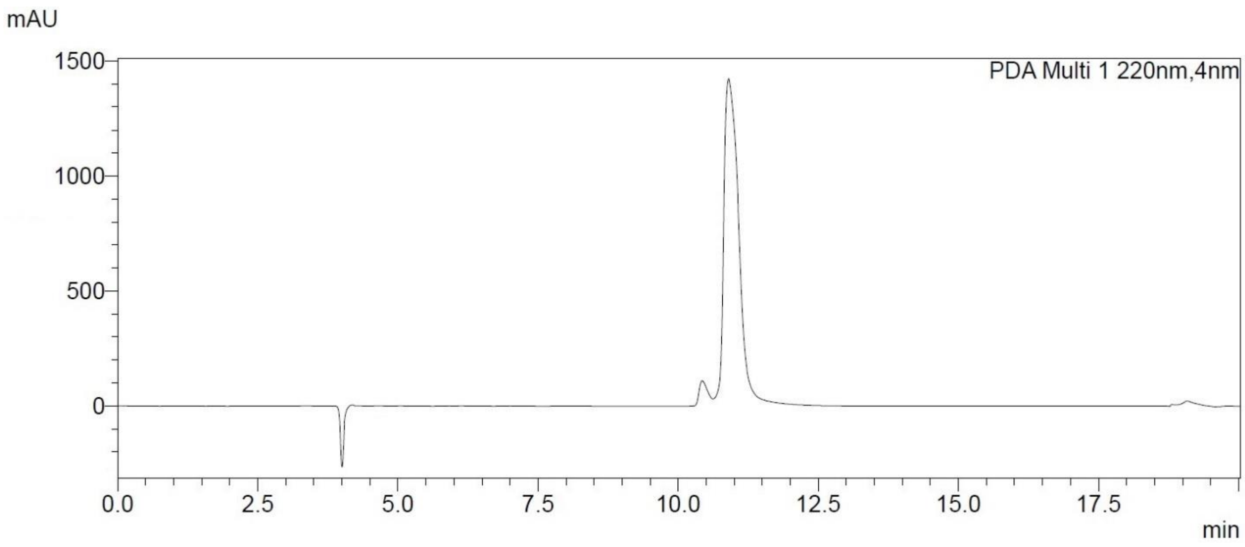


**Figure S40.** HPLC analysis of compound **6d**.

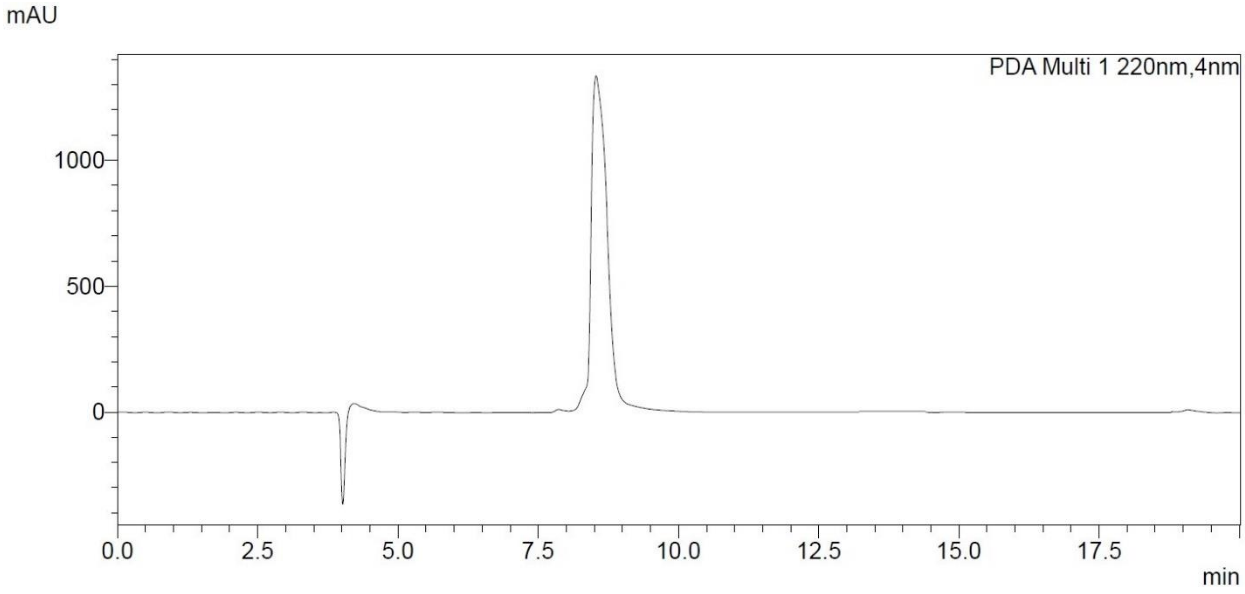


**Figure S41.** HPLC analysis of compound **7a**.

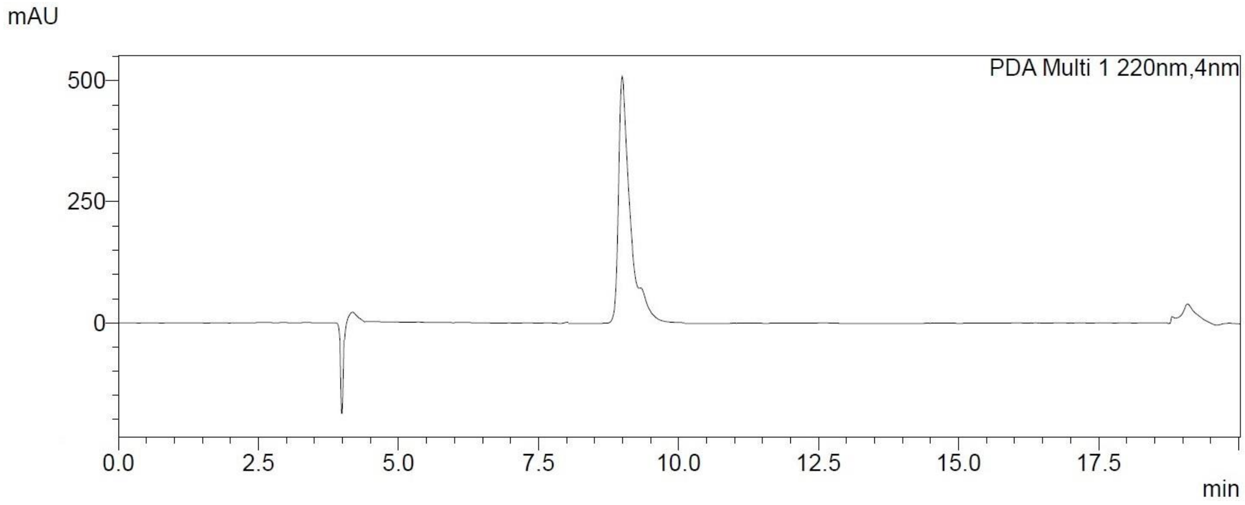


**Figure S42.** HPLC analysis of compound **7b**.

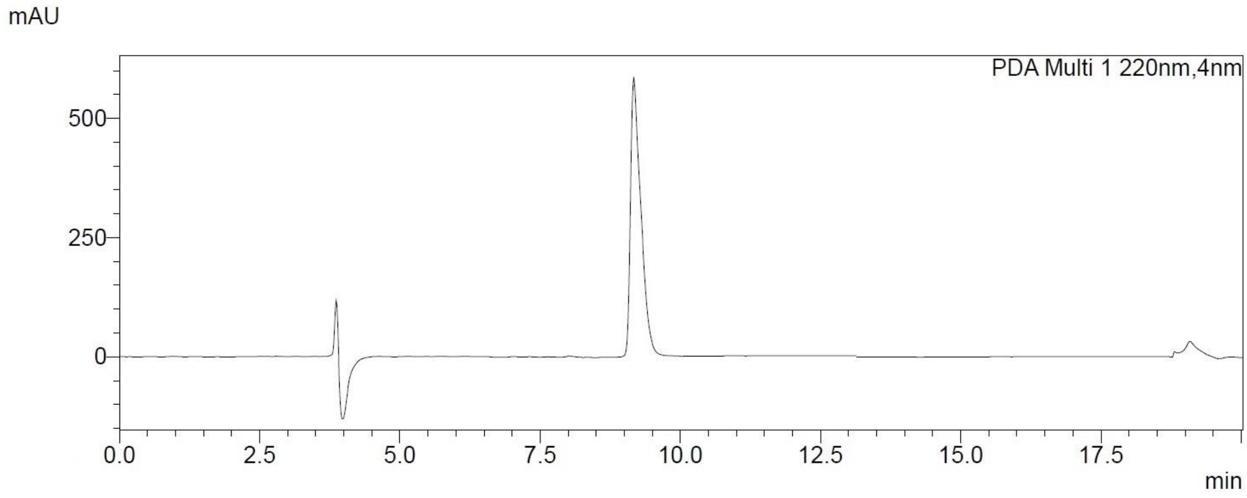


**Figure S43.** HPLC analysis of compound **7c**.

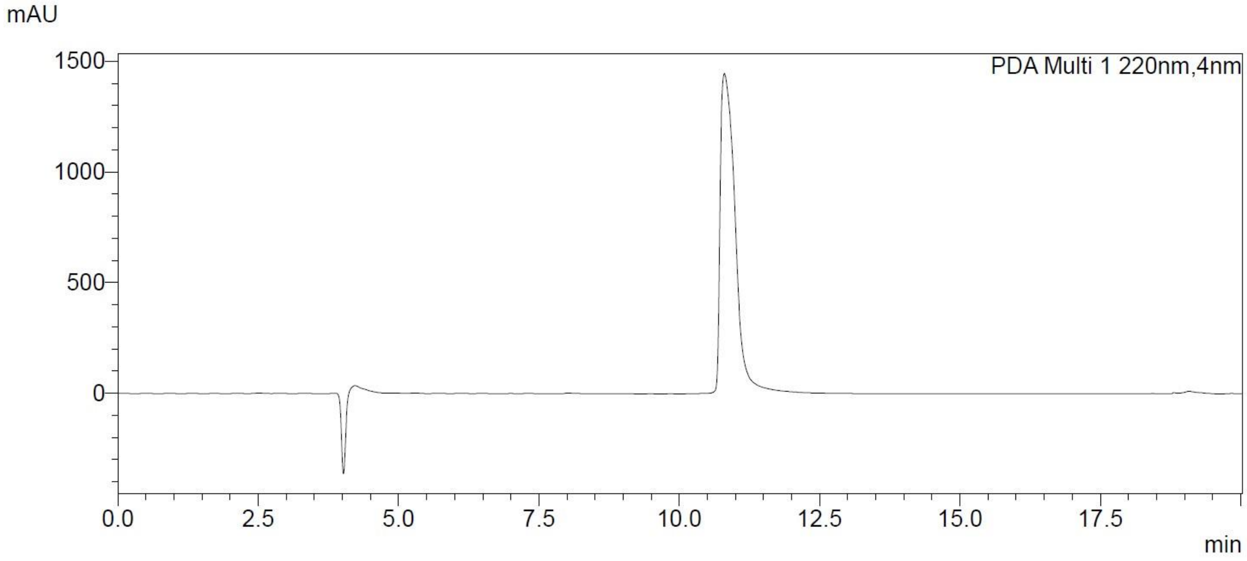


**Figure S44.** HPLC analysis of compound **7d**.

## Additional HPLC Analysis of Lead Peptides

The purity of synthesized cyclic peptides was determined using reverse-phase analytical HPLC (Shimadzu; LC-20ADXR). The conditions used to conduct the purity analysis is provided below:

**<u>Method</u>**

**Column:** Phenomenex (Luna), 4 µm, C18, 150 × 4.6 mm
**Flow rate:** 1 ml/min
**Mobile Phase**: Buffer A-Water containing 0.1% TFA,
Buffer B-Acetonitrile containing 0.1% TFA
**Gradient**

| Time (min) | Buffer B (%) |
|---|---|
| 0.00 | 5 |
| 5 | 20 |
| 10 | 35 |
| 20 | 60 |
| 23 | 95 |
| 25 | 5 |

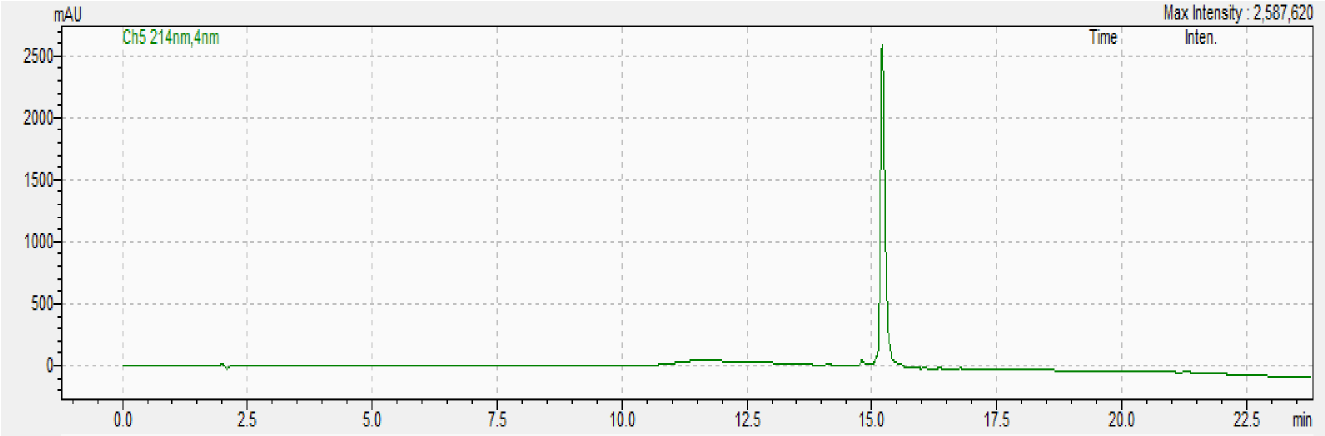


**Figure S45.** HPLC Chromatogram of cyclic peptide [$R_5W_4$] **(5a)**.

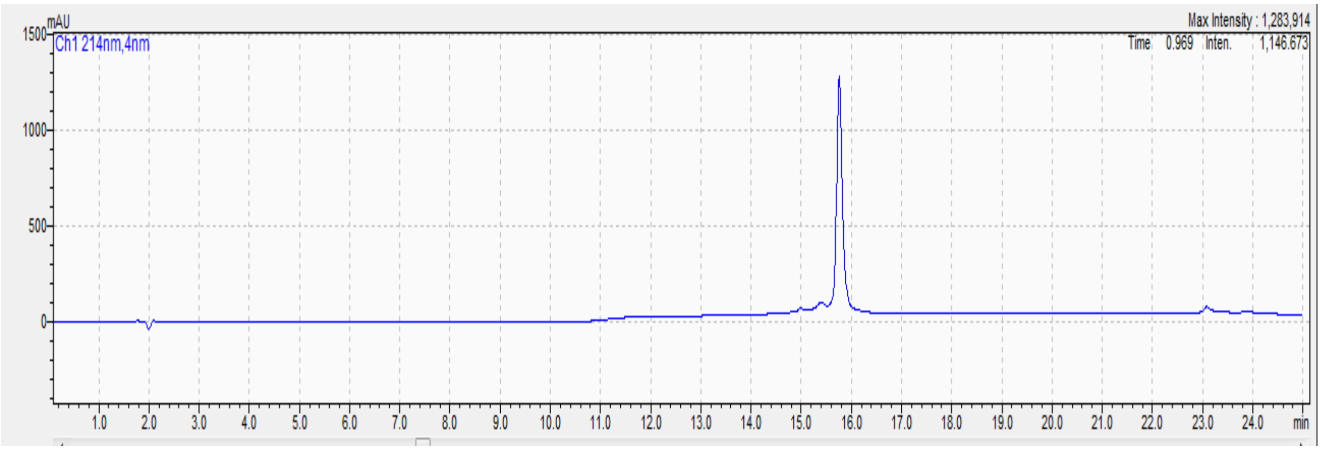


**Figure S46.** HPLC Chromatogram of cyclic peptide [$R_5W_5$] **(5b)**.

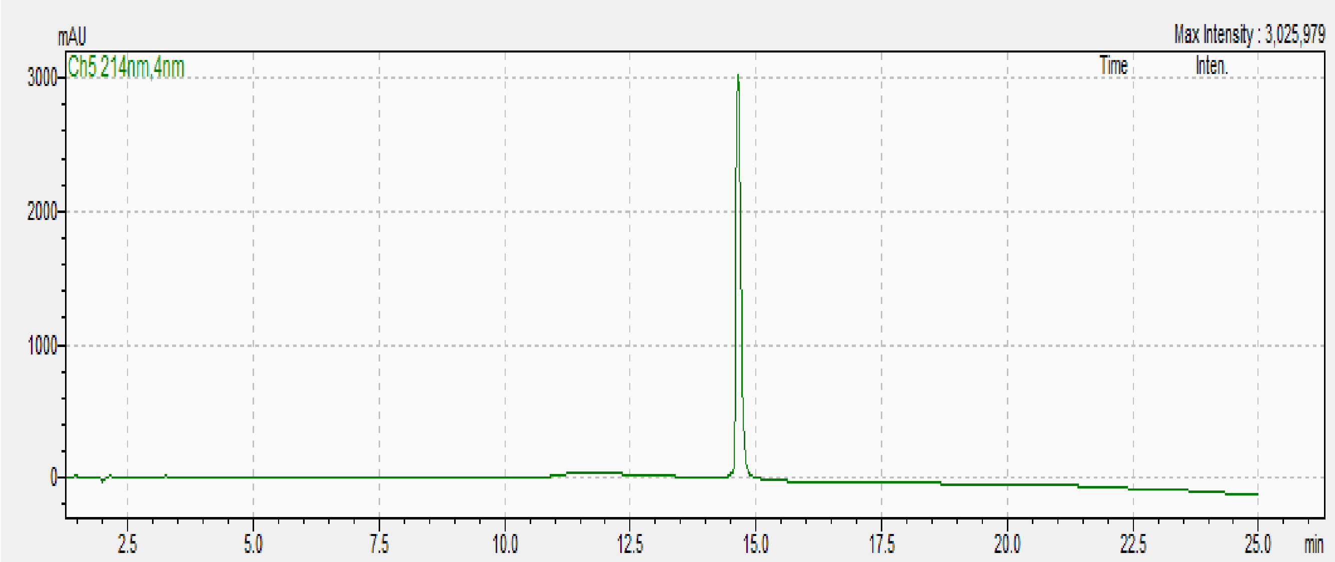


**Figure S47.** HPLC Chromatogram of cyclic peptide [$R_6W_4$] **(6a)**.

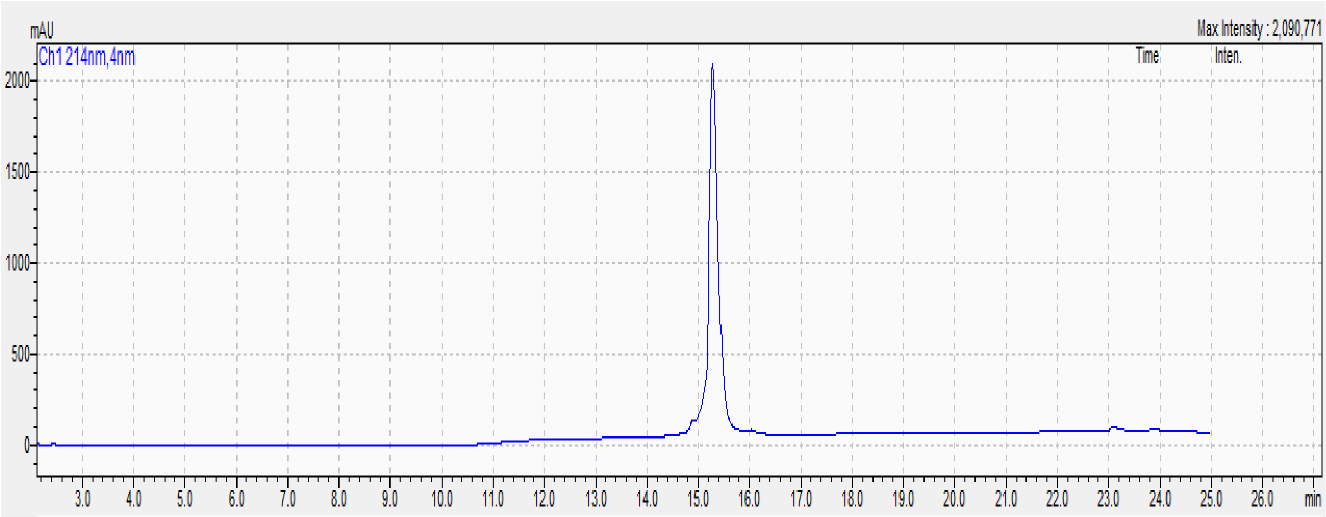


**Figure S48.** HPLC Chromatogram of cyclic peptide [$R_6W_5$] **(6b)**.

| Bacterial strain | Media required for growth | Gram Positive/Negative | Resistant/ Nonresistant |
|---|---|---|---|
| ***Staphylococcus aureus* (ATCC 29213)** | Tryptic Soya Broth (TSB) | Gram-positive | Non-resistant strain |
| ***Staphylococcus aureus (ATCC BAA-1556)*** | Luria Broth (LB) | Gram-positive | Methicillin-resistant bacterial strain |
| ***Enterococcus faecium* (ATCC 27270)** | Brain Heart Infusion Broth (BHIB) | Gram-positive | Non-resistant strain |
| ***Enterococcus faecium* (ATCC 700221)** | Brain Heart Infusion Broth (BHIB) | Gram-positive | vancomycin and teicoplanin resistant bacterial strain |
| ***Enterococcus faecalis* (ATCC 29212)** | Brain Heart Infusion Broth (BHIB) | Gram-positive | Non-resistant strain |
| ***Enterococcus faecalis* (ATCC 51575)** | Brain Heart Infusion Broth (BHIB) | Gram-positive | gentamicin, streptomycin, and vancomycin resistant bacterial strain |
| ***Staphylococcus pneumoniae* (ATCC 49619)** | Tryptic Soya Broth (TSB) | Gram-positive | Non-resistant strain |
| ***Staphylococcus pneumoniae* (ATCC 51938)** | Tryptic Soya Broth (TSB) | Gram-positive | penicillin, clindamycin, cotrimoxazole and erythromycin resistant bacterial strain |
| ***Bacillus subtilis* (ATCC-6633)** | Luria Broth (LB) | Gram-positive | Non-resistant strain |
| ***Bacillus cereus* (ATCC-13061)** | Luria Broth (LB) | Gram-positive | Non-resistant strain |
| ***Escherichia coli (ATCC BAA-2452)*** | Nutrient Broth (NB) | Gram-negative | New Delhi metallo-beta-lactamase (NDM-1) positive resistant bacterial |

| | | | |
|---|---|---|---|
| | | | strain; Carbapenem-resistant bacterial strain |
| ***Pseudomonas aeruginosa (ATCC BAA-1744)*** | Luria Broth (LB) | Gram-negative | Carbapenem-resistant bacterial strain |
| ***Klebsiella pneumonia(ATCC BAA-1705)*** | Luria Broth (LB) | Gram-negative | Imipenem-resistant bacterial strain |
| ***Acinetobacter baumannii (ATCC BAA-1605)*** | Nutrient Broth (NB) | Gram-negative | Resistant to Ceftazidime, Gentamicin, Ticarcillin, Piperacillin, Aztreonam, Cefepime, Ciprofloxacin, Imipenem, and Meropenem. |
| ***Escherichia coli (ATCC 25922)*** | Luria Broth (LB) | Gram-negative | Non-resistant strain |
| ***Pseudomonas aeruginosa (ATCC 27883)*** | Luria Broth (LB) | Gram-negative | Non-resistant strain |
| ***Klebsiella pneumoniae (ATCC 13883)*** | Nutrient Broth (NB) | Gram-negative | Non-resistant strain |
| ***Pseudomonas aeruginosa (ATCC 10145)b*** | Luria Broth (LB) | Gram-negative | Non-resistant strain |

**Table S1.** Media required for growth of the bacterial strains.

| Collection No. | Species | Category | Year | Country | State | Infection type | Age | Gender |
|---|---|---|---|---|---|---|---|---|
| 1188767 | ACB | MDR | 2021 | USA | IL | bloodstream infection | 65 | M |
| 1189854 | ACB | MDR | 2021 | USA | UT | other sites | 43 | M |
| NCTC 13304 | ACB | CRAB (OXA-27) | NR | NR | NR | NR | NR | NR |
| 1191008 | EC | MDR | 2021 | USA | NY | bloodstream infection | 74 | M |
| ATCC 25922 | EC | wild type | NR | NR | NR | NR | NR | NR |
| ATCC BAA-2452 | EC | CRE (NDM-1) | NR | NR | NR | NR | NR | NR |
| 1188718 | KPN | MDR | 2021 | USA | IL | urinary tract infection | 71 | F |
| ATCC 700603 | KPN | ESBL (SHV-18) | NR | NR | NR | NR | NR | NR |
| ATCC BAA 1705 | KPN | CRE (KPC-2) | NR | NR | NR | NR | NR | NR |
| 1188712 | PSA | MDR | 2021 | USA | IL | urinary tract infection | 72 | F |
| 1191191 | PSA | MDR | 2021 | USA | NJ | pneumonia in hospitalized patients | 47 | M |
| ATCC 27853 | PSA | wild type | NR | NR | NR | NR | NR | NR |
| 1193193 | SA | MRSA | 2021 | USA | CO | bloodstream infection | 59 | F |
| 1195201 | SA | MDR | 2021 | Belgium | | pneumonia in hospitalized patients | 26 | F |
| ATCC 29213 | SA | MSSA | NR | NR | NR | NR | NR | NR |

**Table S2.** Bacterial strains and clinical isolates tested in the study. ACB, *Acinetobacter baumannii-calcoaceticus* species complex; ATCC, American Type Culture Collection; CRAB, carbapenem-resistant *Acinetobacter baumannii*; CRE, carbapenem-resistant *Enterobacterales*; EC, *Escherichia coli*; ESBL, extended-spectrum β-lactamase; KPN, *Klebsiella pneumoniae*; MDR, multidrug resistant; MRSA, methicillin-resistant *Staphylococcus aureus*; MSSA, methicillin-susceptible *Staphylococcus aureus*; NCTC, National Collection of Type Cultures; NR, not relevant; PSA, *Pseudomonas aeruginosa*; SA, *Staphylococcus aureus.*

| | | MIC[a] μg/mL | | | | | | | |
|---|---|---|---|---|---|---|---|---|---|
| | | **MRSA (ATCC BAA-1556)** | | ***K. pneumoniae*** **(ATCC BAA-1705)** | | ***P. aeruginosa*** **(ATCC 27883)** | | ***E. coli*** **(ATCC 25922)** | |
| | | **5a** | **6a** | **5a** | **6a** | **5a** | **6a** | **5a** | **6a** |
| **NaCl** | (150 mM)[b] | 4 | 8 | 32 | 64 | 32 | 16 | 16 | 32 |
| **KCl** | (4.5 mM)[b] | 4 | 8 | 32 | 64 | 32 | 16 | 16 | 32 |
| **$MgCl_2$** | (1 mM)[b] | 2 | 4 | 16 | 32 | 16 | 8 | 8 | 16 |
| **$CaCl_2$** | (2 mM)[b] | 4 | 8 | 16 | 64 | 32 | 16 | 16 | 32 |
| **$NH_4Cl$** | (6 mM)[b] | 2 | 4 | 16 | 32 | 16 | 8 | 8 | 16 |
| **$FeCl_3$** | (8 μM)[b] | 4 | 8 | 32 | 64 | 32 | 16 | 16 | 32 |
| **FBS[c]** | (25%)[b] | 4 | 8 | 32 | 64 | 32 | 16 | 16 | 32 |
| **MH[d]** | | 4 | 8 | 32 | 64 | 32 | 16 | 16 | 32 |

**Table S3.** MIC values of peptides **5a** and **6a** in the presence of salts and serum. [a]Minimum inhibitory concentration (MIC) is the lowest concentration of the peptides that inhibited bacterial growth; [b]Final concentration of salts in the tested conditions; [c]Fetal bovine serum; [d](MH): Mueller-Hinton broth media, MIC tested in absences of salts and serum. The Table represents the data of three independent experiments performed in triplicate.

| Tested components | MIC (µg/mL)[a] | | | |
|---|---|---|---|---|
| | *S. aureus* (ATCC BAA-1556) | *K. pneumoniae* (ATCC BAA-1705) | *P. aeruginosa* (ATCC 27883) | *E. coli* (ATCC 25922) |
| Tetracycline | 0.250 | 16 | 32 | 8 |
| Tetracycline + **5a** | 0.065 | 2 | 2 | 1 |
| Tobramycin | 0.5 | 16 | 0.5 | 8 |
| Tobramycin + **5a** | 0.125 | 4 | 0.125 | 2 |
| Levofloxacin | 4 | 64 | 1 | 64 |
| Levofloxacin + **5a** | 1 | 8 | 0.250 | 8 |
| Ciprofloxacin | 16 | 256 | 0.5 | 64 |
| Ciprofloxacin + **5a** | 2 | 8 | 0.125 | 8 |
| Metronidazole | 32 | 256 | 32 | 128 |
| Metronidazole + **5a** | 2 | 8 | 8 | 16 |
| Clindamycin | 0.125 | 512 | 512 | 64 |
| Clindamycin + **5a** | 0.033 | 2 | 4 | 4 |
| Daptomycin | 2 | 512 | 512 | 256 |
| Daptomycin + **5a** | 1 | 16 | 4 | 8 |
| Polymyxin B | 64 | 1 | 1 | 2 |
| Polymyxin + **5a** | 2 | 0.5 | 0.250 | 0.5 |
| Kanamycin | 256 | 64 | 256 | 32 |
| Kanamycin + **5a** | 4 | 8 | 8 | 4 |
| Meropenem | 2 | 16 | 1 | 1 |
| Meropenem + **5a** | 1 | 2 | 0.125 | 0.5 |
| Vancomycin | 1 | 512 | 256 | 256 |
| Vancomycin + **5a** | 0.5 | 4 | 8 | 8 |

**Table S4.** MIC values of physical mixture of **5a** with commercially available antibiotics (1:1 w/w).
[a]All experiments were performed in triplicate.

| Tested components | MIC (µg/mL) [a] | | | |
|---|---|---|---|---|
| | ***S. aureus*** **(ATCC BAA-1556)** | ***K. pneumoniae*** **(ATCC BAA-1705)** | ***P. aeruginosa*** **(ATCC 27883)** | ***E. coli*** **(ATCC 25922)** |
| Tetracycline | 0.250 | 16 | 32 | 8 |
| Tetracycline + **6a** | 0.065 | 4 | 4 | 2 |
| Tobramycin | 0.5 | 16 | 0.5 | 8 |
| Tobramycin + **6a** | 0.125 | 4 | 0.25 | 4 |
| Levofloxacin | 4 | 64 | 1 | 64 |
| Levofloxacin + **6a** | 2 | 16 | 0.5 | 8 |
| Ciprofloxacin | 16 | 256 | 0.5 | 64 |
| Ciprofloxacin + **6a** | 4 | 16 | 0.125 | 8 |
| Metronidazole | 32 | 256 | 32 | 128 |
| Metronidazole + **6a** | 2 | 16 | 4 | 16 |
| Clindamycin | 0.125 | 512 | 512 | 64 |
| Clindamycin + **6a** | 0.033 | 8 | 8 | 8 |
| Daptomycin | 2 | 512 | 512 | 256 |
| Daptomycin + **6a** | 1 | 16 | 8 | 8 |
| Polymyxin B | 64 | 1 | 1 | 2 |
| Polymyxin + **6a** | 4 | 0.25 | 0.5 | 0.5 |
| Kanamycin | 256 | 64 | 256 | 32 |
| Kanamycin + **6a** | 4 | 8 | 4 | 4 |
| Meropenem | 2 | 16 | 1 | 1 |
| Meropenem + **6a** | 1 | 4 | 0.125 | 0.065 |
| Vancomycin | 1 | 512 | 256 | 256 |
| Vancomycin + **6a** | 0.25 | 16 | 8 | 16 |

**Table S5.** MIC values of physical mixture of **6a** with commercially available antibiotics (1:1 w/w).
[a]All experiments were performed in triplicate.

| | 1 | 2 | 3 | 4 | 5 | 6 | 7 | 8 | 9 | 10 | 11 | 12 |
|---|---|---|---|---|---|---|---|---|---|---|---|---|
| A | 512/32 | 256/32 | 128/32 | 64/32 | 32/32 | 16/32 | 8/32 | 4/32 | 2/32 | 1/32 | 0.5/32 | 0/32 |
| B | 512/16 | 256/16 | 128/16 | 64/16 | 32/16 | 16/16 | 8/16 | 4/16 | 2/16 | 1/16 | 0.5/16 | 0/16 |
| C | 512/8 | 256/8 | 128/8 | 64/8 | 32/8 | 16/8 | 8/8 | 4/8 | 2/8 | 1/8 | 0.5/8 | 0/8 |
| D | 512/8 | 256/8 | 128/8 | 64/8 | 32/8 | 16/8 | 8/8 | 4/8 | 2/8 | 1/8 | 0.5/8 | 0/8 |
| E | 512/8 | 256/8 | 128/8 | 64/8 | 32/8 | 16/8 | 8/8 | 4/8 | 2/8 | 1/8 | 0.5/8 | 0/8 |
| F | 512/4 | 256/4 | 128/4 | 64/4 | 32/4 | 16/4 | 8/4 | 4/4 | 2/4 | 1/4 | 0.5/4 | 0/4 |
| G | 512/2 | 256/2 | 128/2 | 64/2 | 32/2 | 16/2 | 8/2 | 4/2 | 2/2 | 1/2 | 0.5/2 | 0/2 |
| H | 512/0 | 256/0 | 128/0 | 64/0 | 32/0 | 16/0 | 8/0 | 4/0 | 2/0 | 1/0 | 0.5/0 | 0/0 |

**Table S6.** Checkerboard Assay of $[R_5W_4]$ + Clindamycin using *K. pneumoniae* (ATCC BAA-1705). The Table shows the concentration profile in a 96 well plate).

| | 1 | 2 | 3 | 4 | 5 | 6 | 7 | 8 | 9 | 10 | 11 | 12 |
|---|---|---|---|---|---|---|---|---|---|---|---|---|
| A | - | - | - | - | - | - | - | - | - | - | - | 32 Peptide MIC |
| B | - | - | - | - | - | - | - | - | | 1/16 Additive effect | + | + |
| C | - | - | - | - | - | - | - | - | 2/8 | + | + | + |
| D | - | - | - | - | - | - | - | - | 2/8 | + | + | + |
| E | - | - | - | - | - | - | - | - | 2/8 | + | + | + |
| F | - | - | - | - | - | + | + | + | + | + | + | + |
| G | - | - | - | + | + | + | + | + | + | + | + | + |
| H | 512 Clindamycin MIC | + | + | + | + | + | + | + | + | + | + | + |

**Table S7.** Results and data analysis of checkerboard Assay of $[R_5W_4]$ + Clindamycin using *K. pneumoniae* (ATCC BAA-1705). (1) Row H (1-11) serial dilution of antibiotic [Clindamycin]; (2) Column 12 (A-G) serial dilution of Peptide$[R_5W_4]$; (3) Well H column 12 is positive control; (4) Clindamycin MIC = 512 µg/mL; (5) Peptide MIC = 32 µg/mL; (6) FIC of Clindamycin = 2; (7) FIC of peptide = 8; (8) $A / MIC_A + B / MIC_B = FIC_A + FIC_B$ = FIC Index; (9) **FICI = 2/512 + 8/32 = 0.0039 + 0.25= 0.2539**

**Plasma stability of 5a conducted in triplicate**

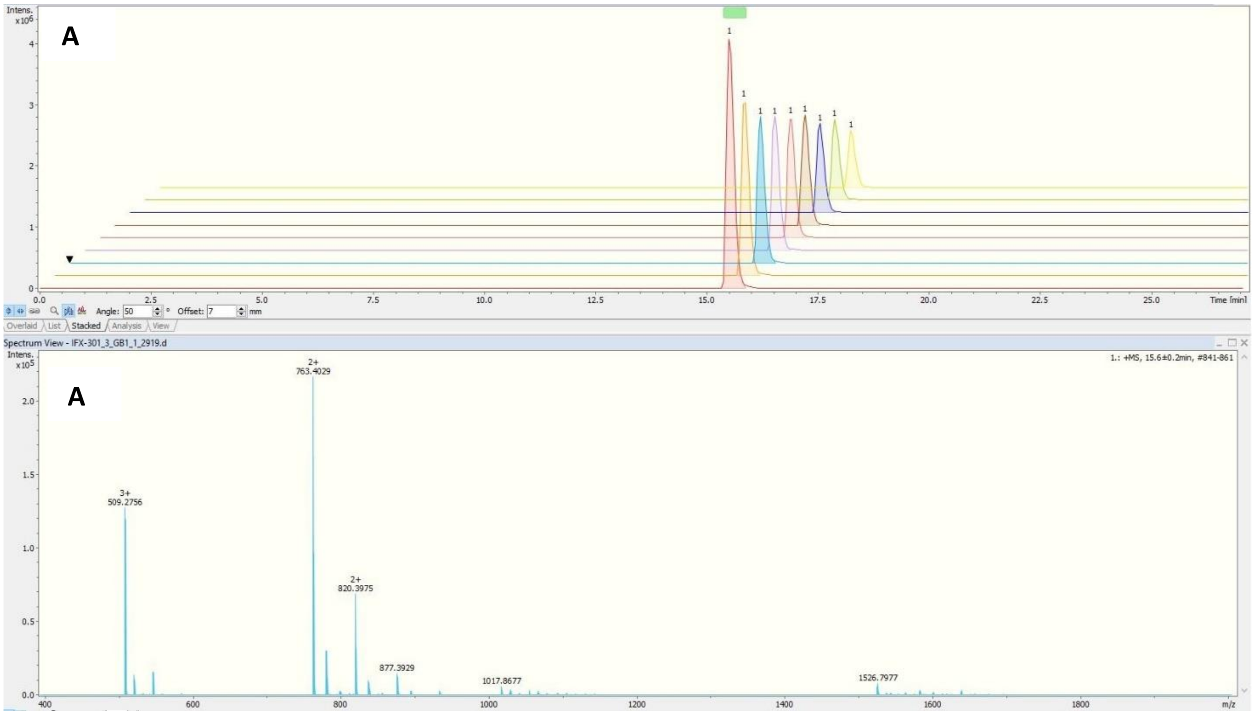


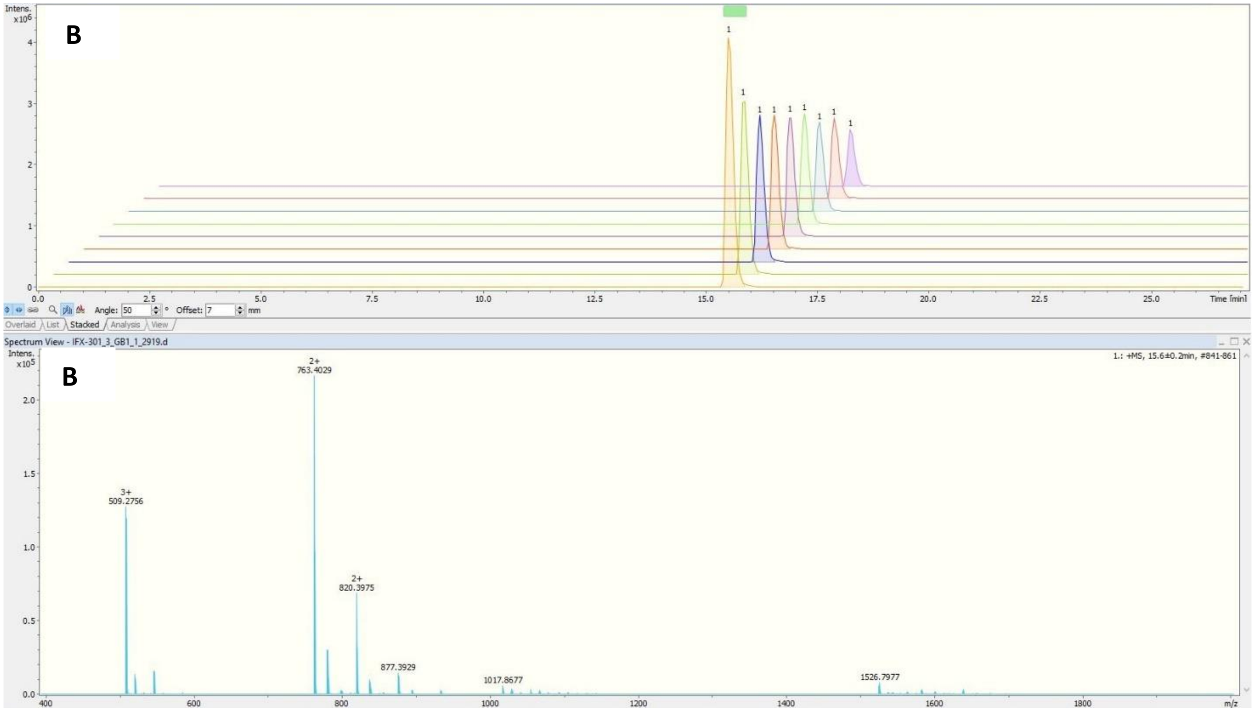

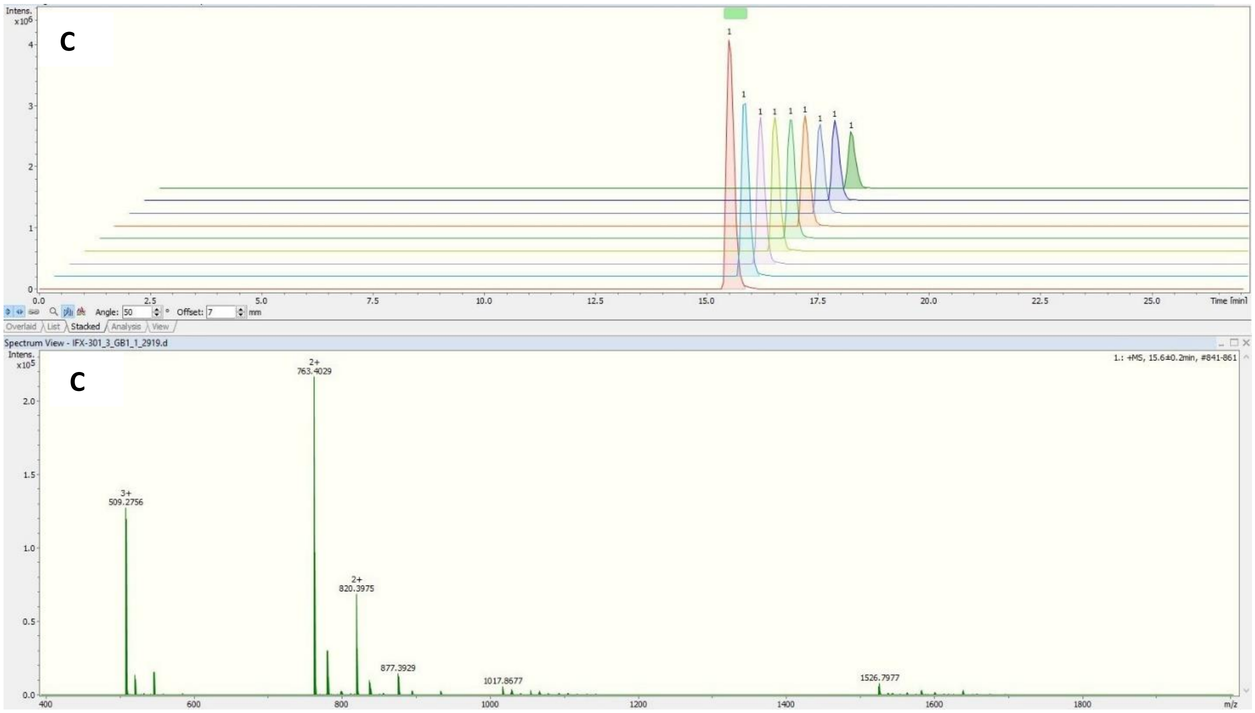


**Figure S49.** Overlay representation of the chromatograms of **5a** conducted in triplicate (A, B, and C).
